# Supplementary material for: Low-temperature hydroformylation of ethylene by phosphorous stabilized Rh sites in a one-pot synthesized Rh-(O)-P-MFI zeolite
Source: Nat Commun. 2023 Nov 7;14:7174. doi: 10.1038/s41467-023-42938-4 (PMC10630368; doi:10.1038/s41467-023-42938-4)
Supplement: Supplementary file 1 — Supplementary Information [file 41467_2023_42938_MOESM1_ESM.pdf]

Supplementary Information

Table of contents

1. Catalyst characterization methods

2. Catalytic tests

3. Experimental section

3.1 Calcined Rh-MFI sample

3.2 Catalytic study of a pre-activated Rh-MFI calcined sample in syngas flow

3.3 Calcined reduced Rh-MFI sample

3.4 Rh impregnated MFI sample

3.5 Rh-(O)-P-MFI sample

4. Kinetic studies in ethylene hydroformylation

5. Propylene hydroformylation with syngas

6. Physico-chemical properties of the catalysts

7. Appendix

1. Catalyst characterization methods

HAADF-STEM and iDPC microscopy. Samples for electron microscopy studies were prepared by dropping a suspension of the solid sample in CH_2Cl_2 directly onto holey carbon-coated copper grids. Electron microscopy measurements were performed using two types of microscopes. Thus, a non-corrected JEOL 2100F microscope operating at 200 kV both in transmission (TEM) and scanning transmission modes (STEM) was used to record the HAADF Z-contrast images at low resolution. The spatial distribution of Rh-P-calred sample was determined using an energy-dispersive X-ray analysis (EDXA) system (Oxford Instruments) attached to a JEOL JEM2100F electronic microscope. HR-HAADF-STEM and iDPC imaging were performed on a double aberration-corrected, monochromated FEI Titan3 Themis 60-300 microscope operating at 300 kV. The latter technique provides atomically resolved images in which the contrasts are related to the atomic number of the elements under the beam instead of the roughly Z^2-dependent contrasts obtained in the HAADF-STEM images. By using a four-segment detector, this technique allows imaging of light elements, such as O, in the presence of heavier ones (Si, Z = 14) under very low electron dose conditions, which is a key aspect of the atomic-scale structural analysis of zeolites, which are very sensitive to electron beams. In particular, 2048 × 2048 STEM-iDPC image pairs were recorded simultaneously using a convergence angle of 18.6 mrad and a camera length of 91 mm. This configuration allowed us to optimize the collection of signals on the HAADF and FEI DF4 detectors. To limit the damage by the electron beam, a fast image recording protocol was used by combining a beam current of 10–30 pA, a 1.25–2.5 μs dwell time and an automated fine-tuning alignment of A1 and C1 using the OptiSTEM software. To obtain images of good quality, the beam current and image acquisition time should be optimized according to the stability of the sample under the beam. All calcined reduced samples were quickly removed to glove box after reduction and the loading of catalysts on Cu grid for TEM measurements were also operated in glove box to avoid air oxidation. After that, the TEM grid was sealed in the vacuum transfer TEM holder and then transferred to the electron microscope for STEM-iDPC measurements.

Inductively Coupled Plasma Mass Spectrometry (ICP). The Rh loading weight was determined from chemical analyses by Inductively Coupled Plasma Mass Spectrometry (ICP), carried out with a Varian 715-ES ICP-Optical Emission spectrometer, after zeolite dissolution in a HNO_3/HCl/HF aqueous solution. The organic content of the as-synthesis materials was measured by elemental analysis performed with a SCHN FISONS elemental analyzer.

Surface area. Textural properties, including BET surface area, micropore volume and external surface area of the samples, were measured by N_2 adsorption/desorption in a Micromeritics ASAP2000.

^{31}P NMR. Solid-state ^{31}P MAS NMR spectra were recorded at room temperature under magic angle spinning in a Brucker AV-400 spectrometer at 161.9 MHz with a spinning rate of 10 kHz and π/2 pulse length of 3.7 μs with spinal proton decoupling and recycle delay of 20 s. The ^{31}P chemical shift was referred to phosphoric acid.

IR-CO

IR characterization using CO as probe molecule were recorded with a Bruker 70V spectrometer using a DTGS detector and acquiring at 4 cm^{-1} resolution. Experiments were done at low temperature (-65 ºC) using an home-made IR cell allowing in situ treatments in controlled atmospheres and temperatures from -176 to 500 ºC and connected to a vacuum system with gas dosing facility. The samples were "ex situ" activated, followed by an "in situ" activation under controlled conditions. Thus, in the case of calcined reduced samples, they were "in situ" treated in H_2 flow (10 mL/min) at 250 ºC for 2.5 h, cooled down to 50 ºC in H_2 flow and followed by vacuum treatment at a final pressure of 10-5 mbar, while in the case of calcined samples they were treated in vacuum at a final pressure of 10-5 mbar at 50 ºC for 1h. After activation, the temperature was decreased to -65 ºC under dynamic vacuum conditions. CO was dosed at -65 °C and at increasing pressure (up to 3 mbar). IR spectra were recorded after each dosage.

Operando IR-MS

Operando IR-MS studies at 9 bar pressure were performed in a commercial IR catalytic cell (Aabspec) connected "online" to a mass spectrometer (MS) (Balzer (QMG 220 M1)). The calcined reduced samples were firstly activated in H_2 flow (10 mL/min) at 250 ºC for 2.5 h, while in the case of calcined sample they were activated in Ar flow at 50 ºC for 1h. After activation, the temperature was set to 50 ºC, and the gas feed switched to reaction conditions (22 mL/min of syngas CO/H_2/Ar (45/45/10, vol%), and 10 mL/min ethylene). After 30 min of stabilization and monitored by MS, the pressure was increased to 9 bar using a back pressure regulator (BPR, Swagelok). After pressurization, the temperature was kept for 30-90 min until stabilization of the mass signal. Then, the temperature was increased to 200 °C at 10 ºC/min and kept there for 1-1.5 h. IR spectra were acquired after all the experimental steps and every 15 min during catalysis. The reaction was monitored by online MS. After the reaction, the cell was depressurized and the reactant gas feed switched to Ar flow 10 mL/min for 1h. Finally, the temperature was decreased to 25 ºC, the cell evacuated until reaching 10-5 mbar and the temperature decreased to -65 ºC for

CO titration experiment. CO titration were performed by following the previous subsection procedure.

Mass analysis was performed using a mass spectrometer Balzer (QMG 220 M1) coupled to IR reaction cells. The m/z values used to monitor each compound were: 28 (CO), 2 (H_2), 26 (ethylene), 30 (ethane), 58 (propanal), 59 (propanol), 18 (H_2O) and 40 (Ar).

In situ XAS spectroscopy.

X-ray Absorption spectra (XAS) of powder pellets were acquired at the Rh K-edge, where the powder material was diluted in boron nitride when necessary to ensure an absorption jump close to 1. Using a Si (311) double crystal monochromator, XAS spectra were collected in transmission mode for the reference samples and in fluorescence mode for the samples by means of a multichannel SDD fluorescence detector available at the CLÆSS beamline of the ALBA synchrotron[1]. Several XAS repeats were collected to ensure reproducibility and statistics for the ex-situ measurements. Spectra processing and analysis was carried out with the Athena software package[2]. The energy scale was calibrated by setting the first inflection point of the Rh metal foil taken as 23220.0 eV. EXAFS were extracted using the AUTOBK algorithm employing a spline in the k range of 0 to 15.9 $Å^{-1}$ for ex situ samples and 0 to 14 $Å^{-1}$ for operando samples, having a R_{bkg} of 1.1. The FEFF6 code[3, 4] was used for scattering path generation, with Rh-O and Rh-Rh paths being generated from the crystal structures of Rh_2O_3 and Rh^0 and Rh-CO paths from model structure mp-683938 from the Materials Project[5, 6]. Multi (k^1, k^2, k^3)-weighted fits of the data were carried out in r-space over r- (1-3.2 Å) and k- ranges as indicated in the Supporting Information. The S_0^2 value was set to 0.9, and a global E_0 was employed with the initial E_0 value set to the first inflection point of the rising edge and the initial change in energy ΔE set to 0.0. Single scattering paths were fit in terms of a Δreff and σ^2, which represent the deviation from the expected interatomic distances and the structural disorder, respectively with initial values of 0.0 Å and 0.003 $Å^2$. A systematic approach was used in adding paths to exclude possible data overfitting. As additional variables were added, we ensured that the reduced χ^2 was minimized for the best fit. Co-linear Rh-CO paths were fit with a common Δreff and σ^2 and consist of 4 paths Rh-C, Rh-O, Rh-O-C, Rh-C-O-C. Up to 9 variables have been considered in the fits, when, considering the exploited k and R ranges, up to 12 degrees of freedom were available. To assess the goodness of the fits both the R_{factor} (%R) and the reduced χ^2 (χ^2_v) were minimized[7]. Best fit models were determined using a grid search with fixed values for path coordination numbers (N) by employing Larch, the Python implementation of Artemis[8].

X-ray absorption near edge structure (XANES) measurements at the P K-edge were made at beamline 9-BM at the Advanced Photon Source (APS) (Argonne National Laboratory, Argonne, Illinois). A Si (111) double crystal monochromator was employed, and the beam was focused using a Pt/alumina toroidal mirror. A flat uncoated silicon mirror facilitated harmonic rejection at 8.93 mrad (cut off energy of 3.47 keV) in addition to the detuning of the monochromator second crystal by 15% of the beam intensity. All the beam path was under He. Absolute energy calibration was made using a P_2O_5 (at 2149 eV). Data were collected in fluorescence mode using a four elements Vortex Si Drift detector. Details on the beamline optics and instruments can be found elsewhere[9].

The sample was prepared as a pellet and placed in a furnace with Kapton® windows[10]. XANES was recorded for the fresh samples, then 3.5% H_2/He was introduced, and temperature increased (10 °C/min) to 250 °C. After 3h, the sample was cooled to room temperature, and XANES measurements were performed. After that, the temperature was increased to 600 °C for an additional 3h. In the end, the sample was cooled down again, and XANES data was recorded. Data were aligned and normalized using the Athena software[2].

2. Catalytic tests

The catalytic activity for ethylene hydroformylation was evaluated in a fixed-bed reactor with an inner diameter of 6.5 mm and length of 133 mm, in the temperature range 50-200 °C at 10 bar. A synthetic syngas mixture CO/H_2/Ar (45/45/10, vol%), ultra-high purity-grade C_2H_4 and N_2 were mixed in molar ratio 1:1:1:0.5 (CO/H_2/C_2H_4/N_2) to reach a total flow rate 35 mL/min for reaction. The line associated to the CO/H_2/Ar mixture was equipped with a carbonyl trap containing activated carbon upstream of the mass flow controller to retain the metallic carbonyls that might be formed in pressurized gas bottles. Before reaction, Rh-MFI-cal was freshly calcined and directly put for reaction. Syngas pre-activated Rh-MFI-cal was in situ activated at 120 °C for 2h under 22 mL/min CO/H_2/Ar (45/45/10, vol%) at atmospheric pressure with a heating rate of 10 °C/min. Rh-MFI-calred and Rh-P-MFI-calred were in situ reduced at 250 °C for 2h under 20 mL/min H_2 flow at atmospheric pressure with a heating rate of 10 °C/min. Typically, 100 mg catalysts (sieved into 200-400 μm) were diluted with 300 mg SiC granules (Fisher Scientific, 600-800 μm) to achieve an isothermal packed bed with a gas hour space velocity (GSHV) of 8000 h^{-1}. Under these conditions, mass transfer limitations can be excluded as shown in Figure S1. Moreover, additional experiments were done by modifying the GHSV (800-8000 h^{-1}) in order to increase the ethylene conversion in the 50-120 °C temperature range. The products at the outlet of the reactor were on-line analyzed with Agilent 8860 equipped with a TCD (HP-Plot/Q plus HP-Molesieve) and an FID (HP-Plot/U) detector. All product lines were heated at 150 °C to prevent condensation of products in the lines. Product quantification was performed using

chromatographic response factors referenced to N_2 as an internal standard. Ethylene conversion, product selectivity, space time yield (STY), turnover frequencies (TOF), apparent activation energies (Ea) and carbon balance were calculated according to equations 1-6. The elemental balance of carbon was 100 ± 5%. The TOF was calculated using the number of Rh sites assuming that all Rh was atomically dispersed.

$$Conversion\ \left(X_{C_2H_4}\right) = \left(\frac{F_{C_2H_6\,out} + F_{C_3H_6O\,out} + F_{C_3H_7OH\,out}}{F_{C_2H_4\,out} + F_{C_2H_6\,out} + F_{C_3H_6O\,out} + F_{C_3H_7OH\,out}}\right) * 100\ (\%) \quad (1)$$

$$S_i = \left(\frac{F_{i\,out}}{F_{C_2H_6\,out} + F_{C_3H_6O\,out} + F_{C_3H_7OH\,out}}\right) * 100\ (\%) \quad (2)$$

$$STY_i\ (mol \cdot g^{-1} \cdot h^{-1}) = \frac{V_{C_2H_4} * X_{C_2H_4} * S_i * 60}{V_m * m_{cat}} \quad (3)$$

$$TOF = \frac{STY_i * M_{Rh}}{m_{cat} * D_{Rh} * W_{Rh}} \quad (4)$$

$$Ln(STY_i) = -\frac{E_a}{RT} + C \quad (5)$$

$$Carbon\ balance = \frac{Total\ carbon\ in\ products}{Total\ carbon\ in\ feed\ components} \quad (6)$$

Where F_i stands for the molar flow of *i*-compound based on N_2 internal standard using calibrated response factors. V_{C2H4} is the flow of C_2H_4, V_m is the molar volume of ideal gas at standard temperature and pressure, M_{Rh} is the molar mass of Rh, D_{Rh} is the dispersion of Rh, herein is 1, W_{Rh} is the loading weight of Rh, R is molar gas constant.

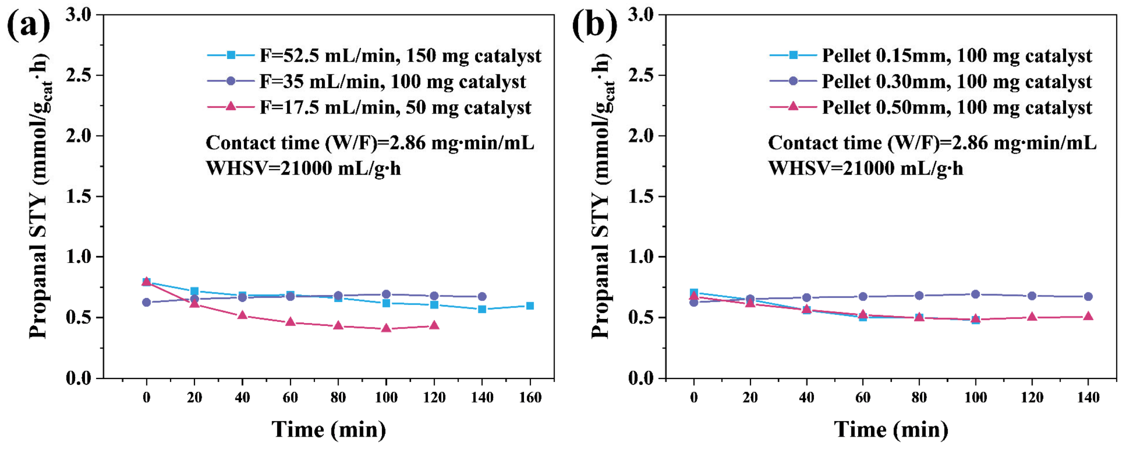

Figure S1. Exclude mass transfer limitations. Propanal STY of Rh-MFI-cal sample under constant contact time with different flow (a) and pellet size (b) to exclude external and internal diffusion limitation.

The reaction orders in ethylene hydroformylation were analysed using a similar procedure as indicated above but operating at low conversion, i.e., high GHSV (18000 h^{-1}). These studies have been done on both Rh-MFI-cal and Rh-MFI-calred, Rh/SiO_2-cal and Rh/SiO_2-calred samples. The reaction orders have been studied at low temperature and under stable conditions (at 50 ºC and 70 ºC respectively) by varying the partial pressure of one reactant from 0.625 to 3.75 bar, while keeping the partial pressure of the other reactant at 2.5 bar.

For experiments with propylene as reactant, 30 mg catalysts (sieved into 200-400 μm) were diluted with 300 mg SiC granules (Fisher Scientific, 600-800 μm) to achieve an isothermal packed bed with a GHSV of 10500 h^{-1}. Experiments were done at 1 bar, in the temperature range 50-120 ºC and using a total flow of 14 mL/min with gas feed of C_3H_8/CO/H_2/N_2 (0.6/0.6/0.6/1). As in ethylene, equations 1-4 were also used for the calculation of propylene conversion, product selectivity, and turnover frequencies (TOF).

3. Experimental section

3.1 Calcined Rh-MFI sample

3.1.1 Synthesis

Rh-MFI is synthetized by conventional hydrothermal synthesis with tetraorthoethylsilicate (TEOS) as silica source and tetrapropylammonium hydroxide (TPAOH) as organic template. The rhodium precursor is a solution of rhodium (III) chloride hydrate and ethylenediamine (Rh-en). In a typical synthesis, the solution of rhodium precursor was prepared by dissolving 0.263 g $RhCl_3$ hydrate (38% Rh) in 90g milliQ water, when the solution became homogeneous, 10g ethylenediamine was added into the solution under stirring. The solution was kept stirring at room temperature for 16 h untill a transparent light yellow solution was formed. Then, 9.5 g TPAOH (43.12%, exchanged from tetrapropylammonium bromide in house using anion exchange resin) and 10g H_2O was mixed in a plastic beaker, followed by adding of 17.36 g TEOS. Then, the beaker was sealed with parafilm and kept under stir for 2 h to allow full hydrolysis of TEOS. Afterward, the parafilm was removed and the solution was allowed to keep under stir for 16 h to evaporate ethanol generated from hydrolysis of TEOS. 5g Rh-en solution was added into the zeolite synthesis gel and was transferred to a Teflon-lined autoclave and heated to 175°C under agitation for 4 days for crystallization. When the crystallization was finished, the solid product was collected by filtration and washed thoroughly with distilled water and dried in air at 100 °C.

The dried sample is calcined in a tubular oven in air (75 mL/min) with a heating rate of 1.5 °C/min to 550 °C and maintain 6 hours. The obtained sample was named Rh-MFI-cal.

3.1.2 Characterization

Electron Microscopy:

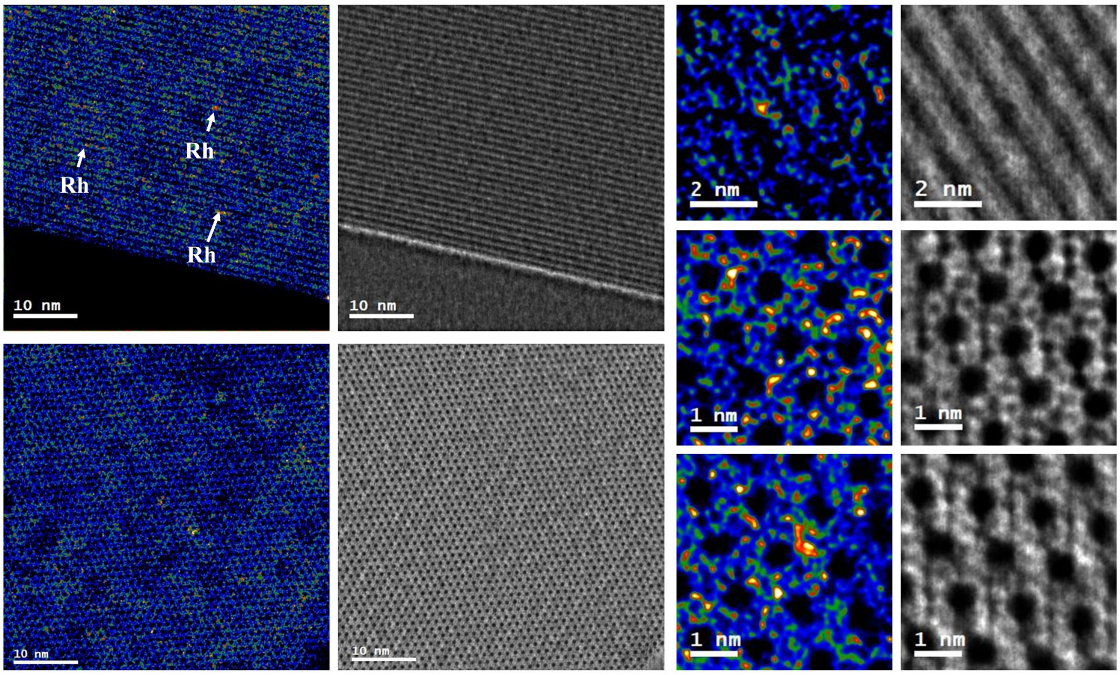

Figure S2. HAADF-STEM and iDPC images of Rh-MFI-cal sample.

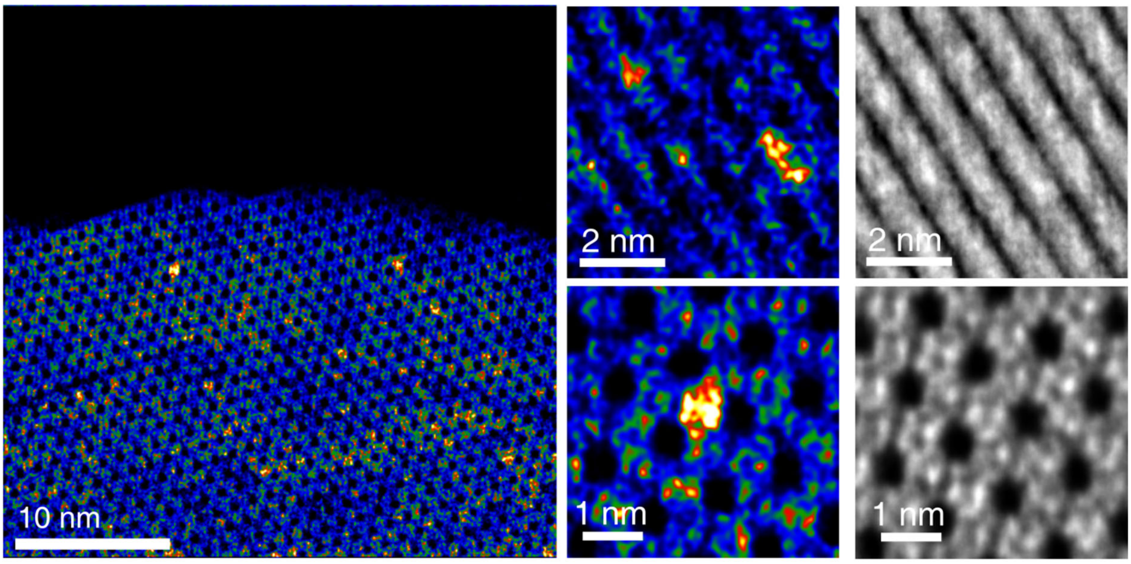

Figure S3. HAADF-STEM and iDPC images of spent Rh-MFI-cal sample at 90 °C

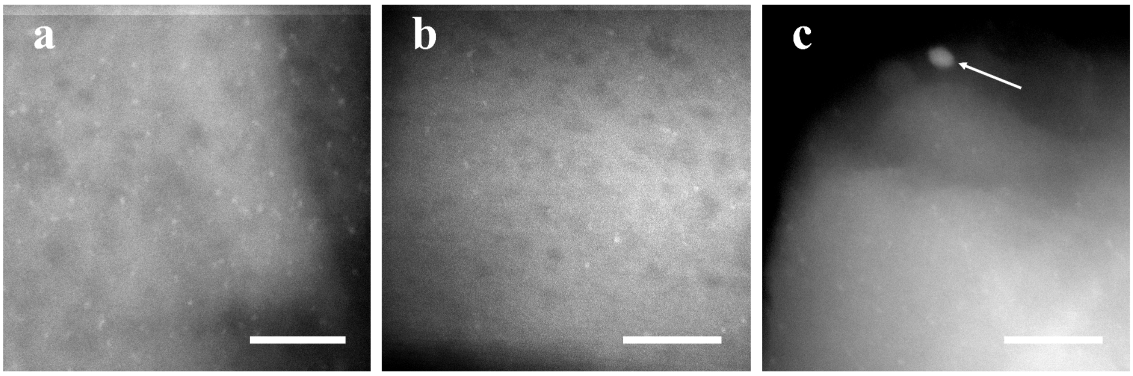

Figure S4. STEM of fresh Rh-MFI-cal sample (a), and spent after hydroformylation conditions at 90 °C (b) and after 200 °C (c). Scale bars corresponding to 20 nm.

3.1.3 Catalytic study in a fixed bed reactor.

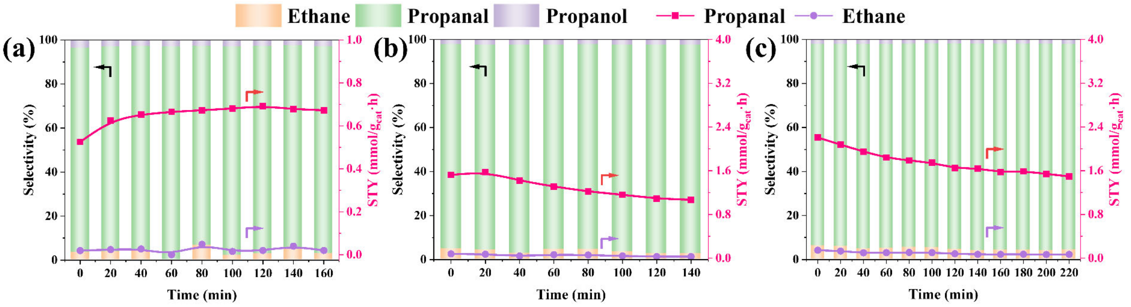

Figure S5. Catalytic performance at (a) 50 °C, (b) 70 °C and (c) 90 °C and 10 bar. Right axe: space time yield (STY in mmol/g_{cat}.h) to propanal (red line) and ethane (violet line); Left axe: selectivity to products (%): propanal (marked as green column), ethane (brown) and propanol (violet). *Reaction conditions*: 100 mg catalyst diluted in 300 mg SiC. 35mL/min total flow. GHSV 8000 h^{-1} (Ethylene: CO: H_2: N_2= 1: 1: 1: 0.5).

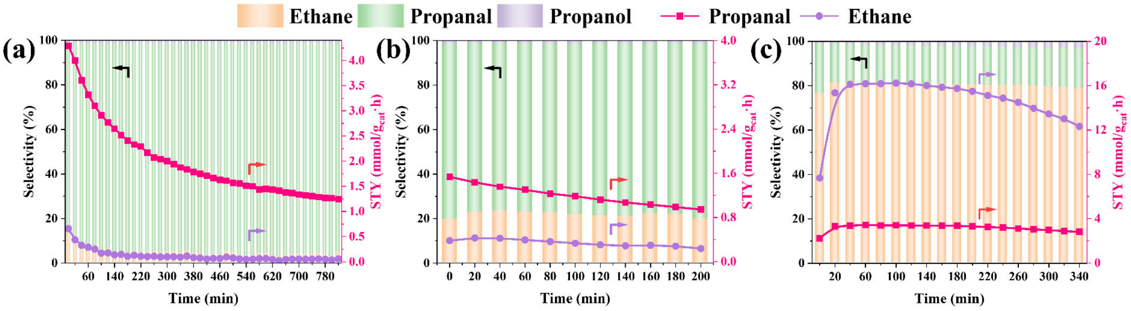

Figure S6. Catalytic performance at (a) 120 °C, (b) 150 °C and (c) 200 °C and 10 bar. Right axe: space time yield (STY in mmol/g_{cat}.h) to propanal (red line) and ethane (violet line); Left axe:

selectivity to products (%): propanal (marked as green column), ethane (brown) and propanol (violet). *Reaction conditions*: 100 mg catalyst diluted in 300 mg SiC. 35 mL/min total flow. GHSV 8000 h^{-1} (Ethylene: CO: H_2: N_2= 1: 1: 1: 0.5).

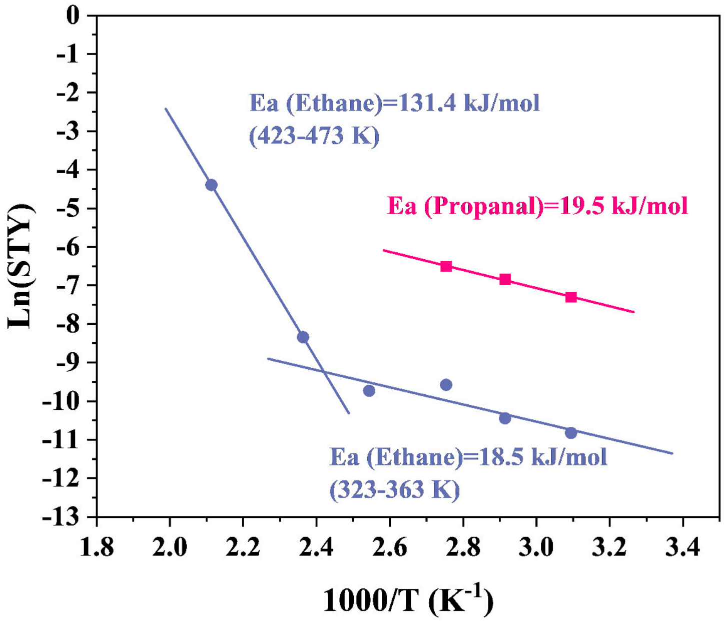

Figure S7. Apparent activation energy of Rh-MFI-cal sample. Arrhenius plot based on reaction rates of ethylene hydroformylation to propanal (red) and ethylene hydrogenation to ethane (violet) in the temperature range 50-90 °C for propanal and 50-200 °C for ethane in the Rh-MFI-cal sample. In the case of ethane, a change in the apparent activation energy is observed when moving from low (50-120 °C) to high (120-200 °C) temperature. This is because of a change in the nature of active sites as demonstrated from operando IR and XAS studies and supported by STEM, where initially single sites predominate whereas at higher temperature, they tend to aggregate into metal clusters.

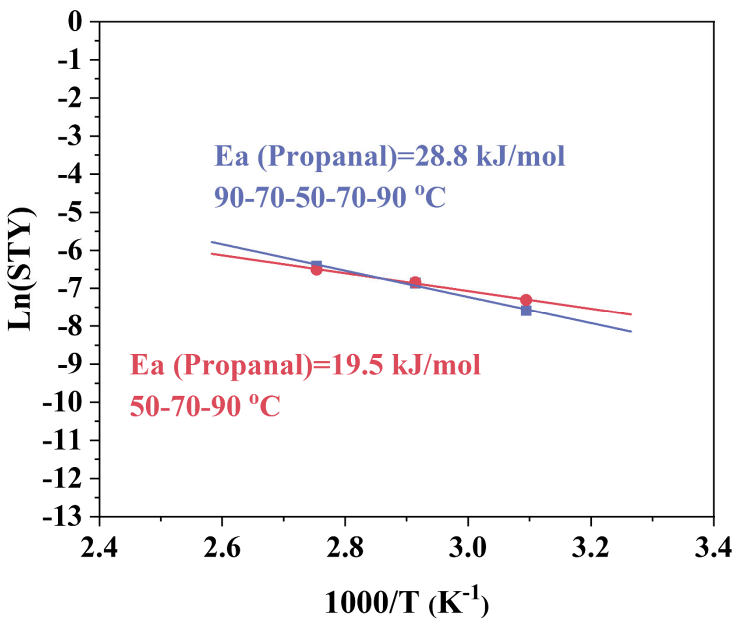

Figure S8. Comparison of apparent activation energy of Rh-MFI-cal sample under different heating mode. Arrhenius plot based on reaction rates of ethylene hydroformylation to propanal in the temperature range 50-90 °C, starting from 50 ºC and then increasing to 70 and 90 ºC (red) and starting at 90 ºC, then decreasing to 70 and 50 ºC and finally back to 70 and 90 ºC in order to compensate for deactivation effects (violet). In both cases the values are very close in line with the stability of the catalyst (Ea of 50-70-90 ºC is based on final stabilized STY values, Ea of 90-70-50-70-90 ºC is based on average STY values at different temperatures).

Table S1. Ethylene conversion and selectivity to propanal on the Rh-MFI calcined sample.

WHSV (mL/g·h)	GHSV (h^{-1})	T (ºC)	Conversion(%)	Selectivity to propanal (%)	STY-Propanal (mmol/g_{cat}·h)	TOF（h^{-1}）
21000 [a]	8000	50	0.23	93.10	0.53	23.50
			0.29	94.33	0.67	30.10
21000 [a]	8000	70	0.67	92.92	1.58	68.22
			0.46	95.04	1.06	47.84
21000 [a]	8000	90	0.98	91.84	2.21	98.68
			0.65	93.59	1.50	66.90
21000 [a]	8000	120 [b]	2.06	85.32	4.29	192.03

			0.54	94.01	1.24	55.51
21000 [a]	8000	150	0.80	79.11	1.54	68.77
			0.49	78.70	0.94	42.28
21000 [a]	8000	200	8.18	16.99	3.39	151.90
			6.40	18.04	2.82	126.13

[a] Without colour are initial values, while those with a green background correspond to final values

[b] Reaction duration was 13h at 120 ºC, while at the others temperatures, 2-6h

Additional experiments are done decreasing the gas hour space velocity (GHSV) from 8000 h^{-1} to 800 h^{-1} in order to increase the conversion of ethylene. As shown in Figure S9, the selectivity to propanal remains above 80% in the 50-120 ºC temperature range at least at conversions up to 7%.

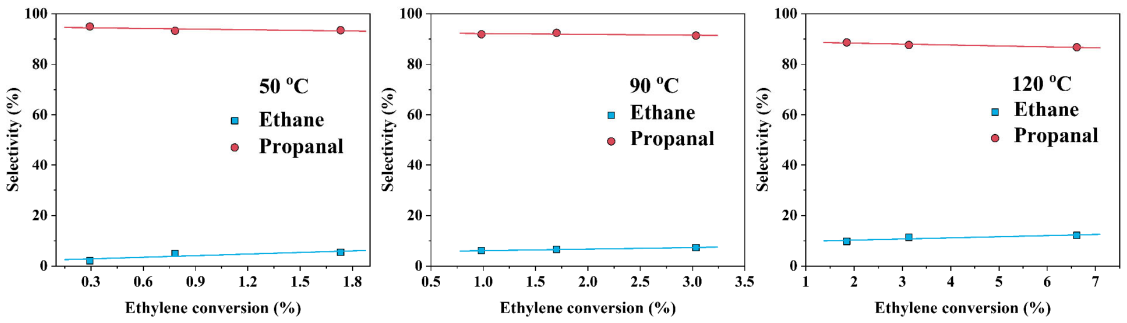

Figure S9. Variation of the selectivity to propanal with the conversion of ethylene on the Rh-MFI-cal sample at 50 °C, 90 °C and 120 °C. Reaction conditions: Ethylene: CO: H_2: N_2= 1: 1: 1: 0.5, 10 bar, GSHV from 8000 to 800 h^{-1}.

Table S2. Comparative performance of the here reported catalysts with state-of-the-art-catalysts used in ethylene hydroformylation with syngas. All the catalysts are included in Figure 3b of the manuscript.

Label on Fig. 3b, c	Catalyst	T (ºC)	P (bar)	Ethane Ea (kJ/mol)	Propanal Ea (kJ/mol)	Propanal TOF (h^{-1}) [a]	Selectivity to propanal [a]	Deactivation constant (K_D)	Reference
1	Rh-MFI-cal	50	10			31.0	95		This work
2	Rh-MFI-cal	70	10	131.4 ± 0.2	19.5 ± 1.5	70.6	95	0.00472[b]	This work
3	Rh-MFI-cal	90	10			98.7	92		This work
4	Rh-MFI-calred	50	10			30.5	91		This work
5	Rh-MFI-calred	70	10	95.5 ± 0.5	31.6 ± 2.1	41.4	95	0.00382[b]	This work
6	Rh-MFI-calred	90	10			58.8	96		This work
7	Rh-(O)-P-MFI-calred	50	10			45.9	95		This work
8	Rh-(O)-P-MFI-calred	70	10	89.2 ± 0.6	26.5 ± 2.2	62.6	95	0.00311[b]	This work
9	Rh-(O)-P-MFI-calred	90	10			70.0	95		This work
10	Rh_2P/SiO_2	180	50	N/A	N/A	190	75	N/A	11
11	$0.6Rh0.23Co/SiO_2$	180	10	74	38	42	52	~0	12
		50	10			<1[c]			
12	Rh/Al_2O_3	150	1	110 ± 2	48 ± 6	~0.1	20	N/A	13
		50	1			<0.1[c]			
13	$Rh-2.5ReO_x/Al_2O_3$	150	1	101 ± 5	47 ± 0	~2.3	45	N/A	13
		50	1			<1[c]			
14	$Rh-V/SiO_2$	115	1	N/A	N/A	0.24	7	N/A	14

	Rh-Zn-SiO_2	180	1	137-167	59 ± 8	~1	N/A	N/A	15
15	Rh_1/SnO_2	130	20	N/A	N/A	101[d]/~20[e]	99	N/A	16
16	Rh_1/SnO_2	150	20	N/A	N/A	225[d]/~10[e]	96	1.22	16
17	Rh_1-0.7W/Al_2O_3	130	10	~70[f]	20[f]	21.6	91	~0	17
		50	10			~5[c]			
18	$Rh_{12}(CO)_{30}/SiO_2$	120	1	N/A	N/A	~1.5	35.9	N/A	18
19	$Li_2[Rh_{12}(CO)_{30}]/SiO_2$	120	1	N/A	N/A	~1.5	53.7	N/A	18
20	$Na_2[Rh_{12}(CO)_{30}]/SiO_2$	120	1	N/A	N/A	~3	52.6	N/A	18
21	$K_2[Rh_{12}(CO)_{30}]/SiO_2$	120	1	N/A	N/A	~1.5	54.3	N/A	18
22	$Zn[Rh_{12}(CO)_{30}]/SiO_2$	120	1	N/A	N/A	~3.6	57.9	N/A	18
23	$Rh_4(CO)_{12}/LiCl/SiO_2$	180	20	100.8	84.5	26.5[g]	21	N/A	19
24	Rh-Mo/SiO_2	115	1	N/A	N/A	9.2	3.3	N/A	20
25	Rh/SiO_2	240	1	21.1 ± 0.8	13.7 ± 0.2	3.4	11.9	N/A	21
		50	1			<0.6[c]			
26	Rh dimer/SiO_2	140	0.4	N/A	N/A	0.0186	26.1	N/A	22
27	RhZn/DeAlBEA	130	1	68.2 ± 9	41.1 ± 3.8	12	79	~0	23
		50	1			<1[c]			
28	RhCo/DeAlBEA	100	1	104.2 ± 3.6	90 ± 4	~20	~75	~0	23
		50	1			<1[c]			
29	RhCo/DeAlBEA	130	1	104.2 ± 3.6	90 ± 4	153	69	~0	23
		50	1			<1[c]			

30	$RhCo_3$/MCM-41	160	1	91 ± 2	55 ± 2	~130	60	~0	24
		50	1			<1[c]			
31	Rh/Al_2O_3	100	10	100 ± 7	51 ± 5	0.018	42 ± 2	~0	25
		50	10			<0.1[c]			
32	HDPA-Rh/Al_2O_3	100	10	89 ± 6	60 ± 4	19 ± 5	89 ± 4	~0	25
		50	10			~1[c]			

[a] TOF and selectivity values for this work are based on the stabilized maximum points.

[b] Deactivation constant (K_D) determined at 120 °C. The procedure is indicated below.

[c] TOF values at 50 °C. This value is determined by extrapolating literature data using the Arrhenius equation of propanal activation energy in the calculation.

[d] Initial ethylene hydroformylation TOF determined by extrapolating activity data to zero time-on-stream and expressed per unit total Rh atom.

[e] Propanal TOF value in the pseudo-steady state.

[f] Apparent activation energy (Ea) for ethane and propanal formation measured in the temperature range 120-160 °C at a total pressure of 1bar.

[g] yield, unit: 10^{-6} mol/min·g_{cat}

Table S3. Conversion of ethylene and selectivity to propanal on the most active state-of-the-art phosphine free and phosphine based catalysts in ethylene hydroformylation.

Label on Fig. 3c	Catalyst	WHSV (mL/g·h)	GHSV (h^{-1})	T (ºC)	P (bar)	Conversion (%)	Selectivity (%)	STY (mmol$_{C2H4\ converted}$/g$_{cat}$·h)	STY (mmol$_{propanal\ formed}$/g$_{cat}$·h)	TOF (h^{-1})	Ref
33	Rh/MCM-41	3600	-	200	1	24.5	9.9	9.0	0.9	156	26
34	Rh_1Co_1/MCM-41	3600	-	200	1	41.7	23.8	15.4	3.7	210	26
35	Rh_1Co_3/MCM-41	3600	-	200	1	52.4	16.5	19.3	3.2	192	26
36	Rh-Co/Al_2O_3	50000	-	200	20	~6.0	19.6	-	-	94.5	27
37	Rh-(O)-P-MFI-calred	21000	8000	200	10	13.7	21.0	33.3	7.0	266.3	This work
38	Rh-MFI-cal	2100	800	120	10	6.6	86.7	-	-	-	This work
14	Rh-V/SiO_2	3600	3600	115	1	0.2	7	0.098	0.0069	0.24	14
39	Rh/SiO_2	6000	-	180	10	4	45	2.5	1.1	42	12
11	0.6Rh0.23Co/SiO_2	6000	-	180	10	~9	52	5.5	2.9	42	12
40	Rh-0.7W/Al_2O_3	6428	-	100	10	12	94	10.5	9.9	-	17
41		2142				27	96	7.9	7.6		
30	$RhCo_3$/MCM-41	3600	-	160	1	17.7	60	6.5	3.9	~130	24
	Rh/2.9ReO_x-Al_2O_3	46000	-	150	1	-	45	-	-	~3.6	13
	DPPPTS-$RhAl_1$/SiO_2 [b]	-	2000	120	10	-	-	-	-	134	28
	Rh-Dpppe/SiO_2 [b]	6000	-	140	10	-	97	-	-	1172	29

	Rh-Xantphos-SILP/NC [b]	7400	-	120	8	~70	95	-	-	600	30
	Rh/POL-PPh_3 [b]	-	2000	120	10	96.2	96.1	-	-	4530	31
	Rh/POL-PPh_3 [b]	-	5000	120	10	88.8	95.4	-	-	10373	31
	Rh_2P/SiO_2 [b]	-	2773	200	50	53	75	-	-	~190	11

[a] With a green background correspond to reactions at 200 °C.

[b] With a brown background correspond to phosphine based catalysts.

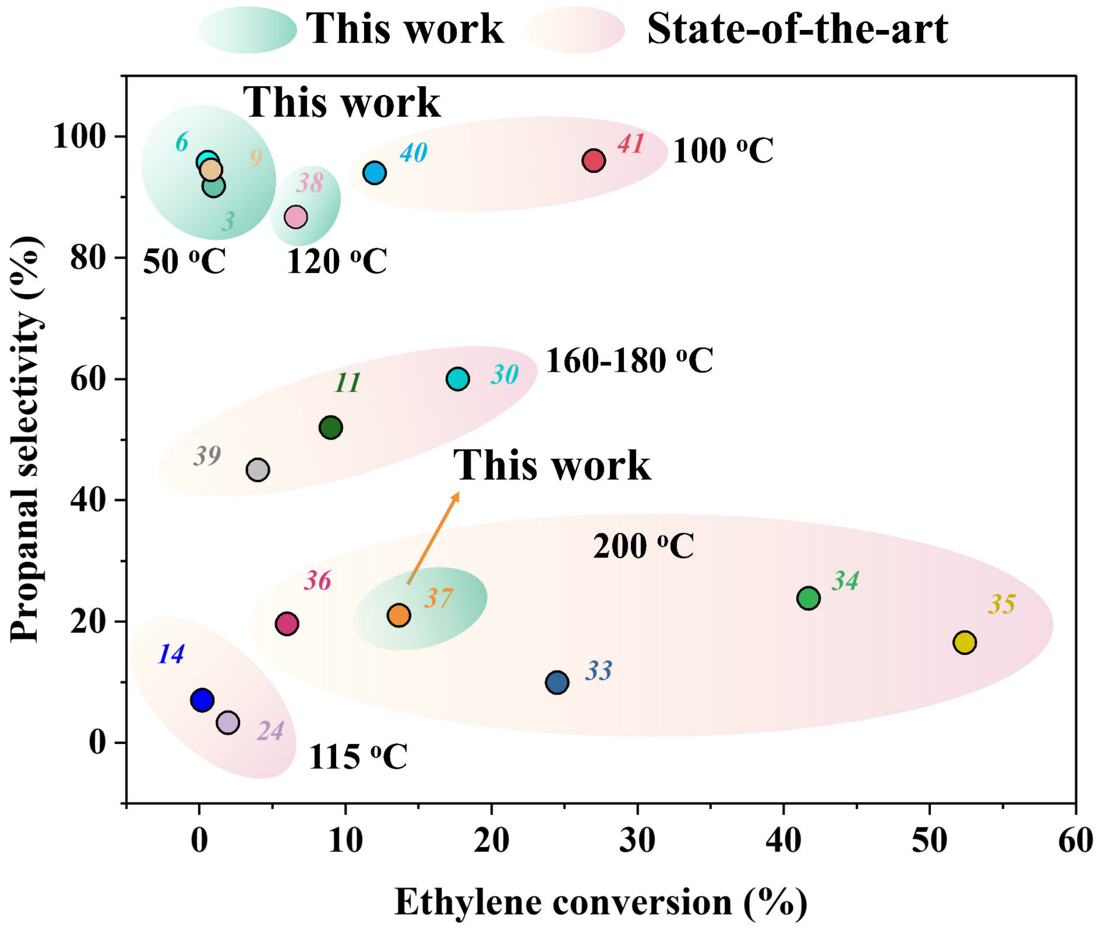

Figure S10. Ethylene conversion and propanal selectivity relationship of all state-of-the-art-phosphine free solid catalysts. References and reaction conditions in Table S2 and S3.

3.1.4 Deactivation constant

Deactivation constant (K_D) is normally used to evaluate catalyst stability, herein, we calculated K_D of Rh-MFI-cal, Rh-MFI-calred and Rh-P-MFI-calred catalysts at 120 °C. In order to assess deactivation quantitatively, the corresponding activity curves were fitted to a second-order kinetics deactivation model (Eq. 7, 8 and 9)[16].

$$a = \frac{TOF_{t=t}}{TOF_{t=max}} \qquad \text{Eq.7}$$

$$-\frac{da}{dt} = K_D a^2 \qquad \text{Eq.8}$$

$$\frac{1}{a} = \frac{1}{a_0} + K_D t \quad \text{Eq.9}$$

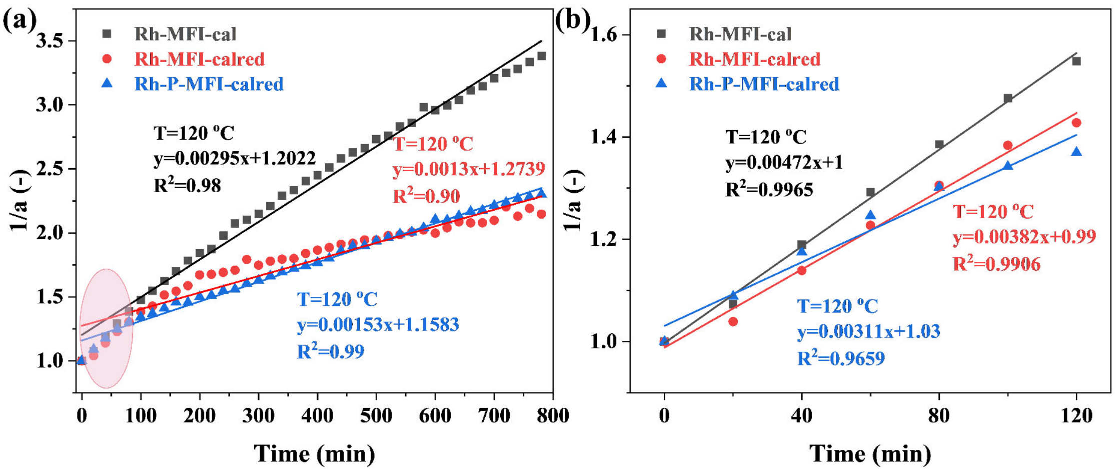

Figure S11. (a) Deactivation constant values of ethylene hydroformylation data recorded with Rh-MFI-cal (black), Rh-MFI-calred (red) and Rh-P-MFI-calred (blue) to a second-order deactivation model; (b) The linear relationships were fitted using the data from the first 2h, which were used in above state-of-the-art table.

3.1.5 Operando studies

X-ray adsorption spectroscopic studies

The X-ray adsorption spectroscopic characterization of Rh-MFI-cal sample at the Rh K-edge compared to reference samples is given in Figure S12. The EXAFS analysis is included in Table S4, 5, 6 and displayed in Figure S13, 14, 15, 16.

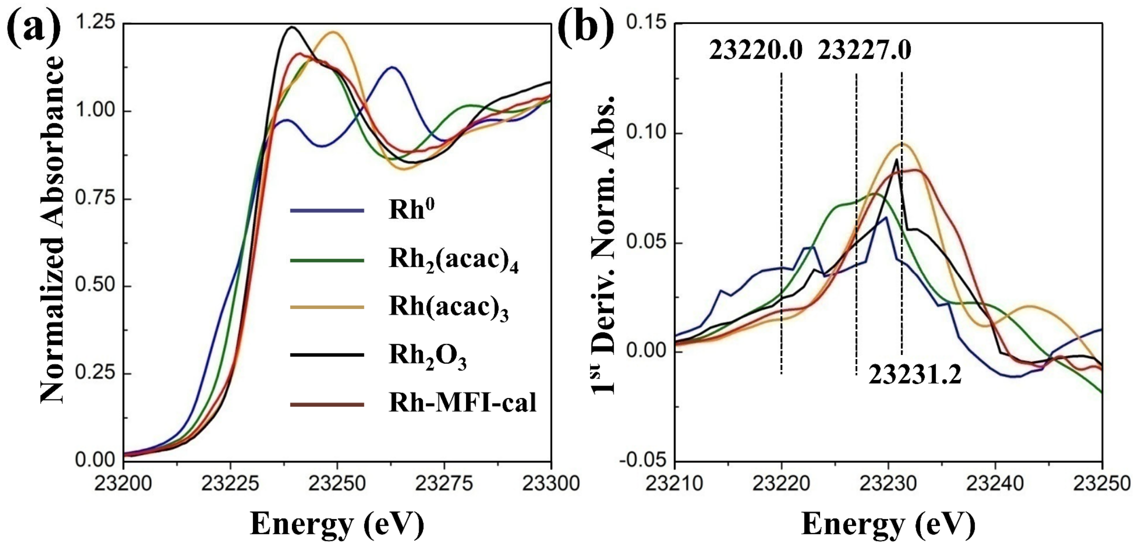

Figure S12. X-ray adsorption spectroscopic characterization of Rh clusters at the Rh K-edge. (a) XANES spectra of Rh-MFI-cal overlaid with references having 0 (Rh^0), +2 ($Rh_2(acac)_4$) and +3 ($Rh(acac)_3$, Rh_2O_3) oxidation states. (b) 1st derivative of the XANES spectra showing E_0' values highlighting the overlap of the Rh-MFI-cal rising edge with that of +3 Rh species at ~23231.2 eV.

Table S4. Multi (k^1, k^2, k^3)-weighted fits carried out in r-space (1-3.2 Å) over a k-range of 3-15 $Å^{-1}$ using a Hannings window (dk 1), and S_0^2= 0.9. Bond distances and disorder parameters (Δr_{eff} and σ^2) were allowed to float having initial values of 0.0 Å and 0.003 $Å^2$ respectively, with a universal E_0 and ΔE_0 = 0 eV.

Rh-MFI-cal

	R_{FACTOR}	X^2_ν	Var. No.	k-range ($Å^{-1}$)	r-range (Å)	ΔE_0 (eV)
	0.025	19.6	7	3-15	1-3.2	2.3 (1.2)

PATHS

	N	r (Å)	σ^2 (x10^3 $Å^2$)
**Rh-C/N/O**	3.8	2.04 (0.01)	3.0 (0.5)
**Rh-Rh**	**N**	**r (Å)**	**σ^2 (x10^3 $Å^2$)**
	0.8	2.71 (0.04)	8.8 (3.7)
	**N**	**r (Å)**	**σ^2 (x10^3 $Å^2$)**
	1.5	3.09 (0.01)	4.6 (1.3)

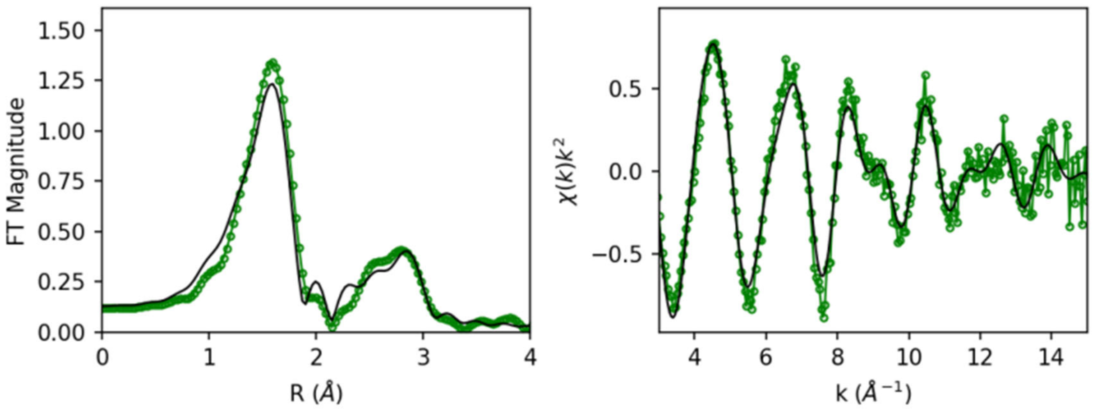

Figure S13. EXAFS fit of Rh-MFI-cal presented in Table S4.

<u>Operando studies: EXAFS analysis of Rh-MFI-cal reacted at 50 °C at 1 and 10 bar.</u>

Table S5. Multi (k^1, k^2, k^3)-weighted fits carried out in r-space (1-3.2 Å) over a k-range of 3-12 $Å^{-1}$ using a Hannings window (dk 1), and S_0^2= 0.9. Bond distances and disorder parameters (Δr_{eff} and σ^2) were allowed to float having initial values of 0.0 Å and 0.003 $Å^2$ respectively, with a universal E_0 and ΔE_0 = 0 eV.

Reaction feed 50 °C 10 bar

	**RFACTOR**	**X^2_ν**	**Var. No.**	**k-range ($Å^{-1}$)**	**r-range (Å)**	**ΔE_0 (eV)**
	0.013	1.6	9	3-12	1-3.2	-6.0 (2.6)

PATHS

**Rh-C/N/O**	**N**	**r (Å)**	**σ^2 (x10^3 $Å^2$)**
	1.8	1.84(0.03)	3.0(0.0)
	**N**	**r (Å)**	**σ^2 (x10^3 $Å^2$)**
	3.2	2.00(0.02)	3.3(0.8)
**Rh-Rh**	**N**	**r (Å)**	**σ^2 (x10^3 $Å^2$)**
	0.5	2.61(0.05)	9.4(3.3)
	**N**	**r (Å)**	**σ^2 (x10^3 $Å^2$)**
	1.0	3.06(0.02)	4.8(2.2)
**Rh-O**	**N**	**r (Å)**	**σ^2 (x10^3 $Å^2$)**
	1.0	2.99(0.03)	3.0(0.0)
**Rh-O-C**	**N**	**r (Å)**	**σ^2 (x10^3 $Å^2$)**
	1.0	2.99(0.03)	3.0(0.0)

Rh-C-O-C	N	r (Å)	σ^2 (x10^3 Å^2)
	0.5	2.99(0.03)	3.0(0.0)

Reaction feed 50 ^{o}C 1 bar

	R$_{FACTOR}$	X^2_ν	Var. No.	k-range (Å^{-1})	r-range (Å)	ΔE_0 (eV)
	0.010	0.6	9	3-12	1-3.2	-3.4(2.1)

PATHS

Rh-C/N/O	N	r (Å)	σ^2 (x10^3 Å^2)
	1.5	1.87(0.02)	3.0(6.5)
	N	r (Å)	σ^2 (x10^3 Å^2)
	2.0	2.03(0.02)	4.1(1.0)
Rh-Rh	N	r (Å)	σ^2 (x10^3 Å^2)
	0.8	2.74(0.01)	4.7(1.3)
	N	r (Å)	σ^2 (x10^3 Å^2)
	1.0	3.11(0.02)	6.4(2.5)
Rh-O	N	r (Å)	σ^2 (x10^3 Å^2)
	3.0	3.02(0.02)	3.0(6.5)
Rh-O-C	N	r (Å)	σ^2 (x10^3 Å^2)
	2.0	3.02(0.02)	3.0(6.5)
Rh-C-O-C	N	r (Å)	σ^2 (x10^3 Å^2)
	1.0	3.02(0.02)	3.0(6.5)

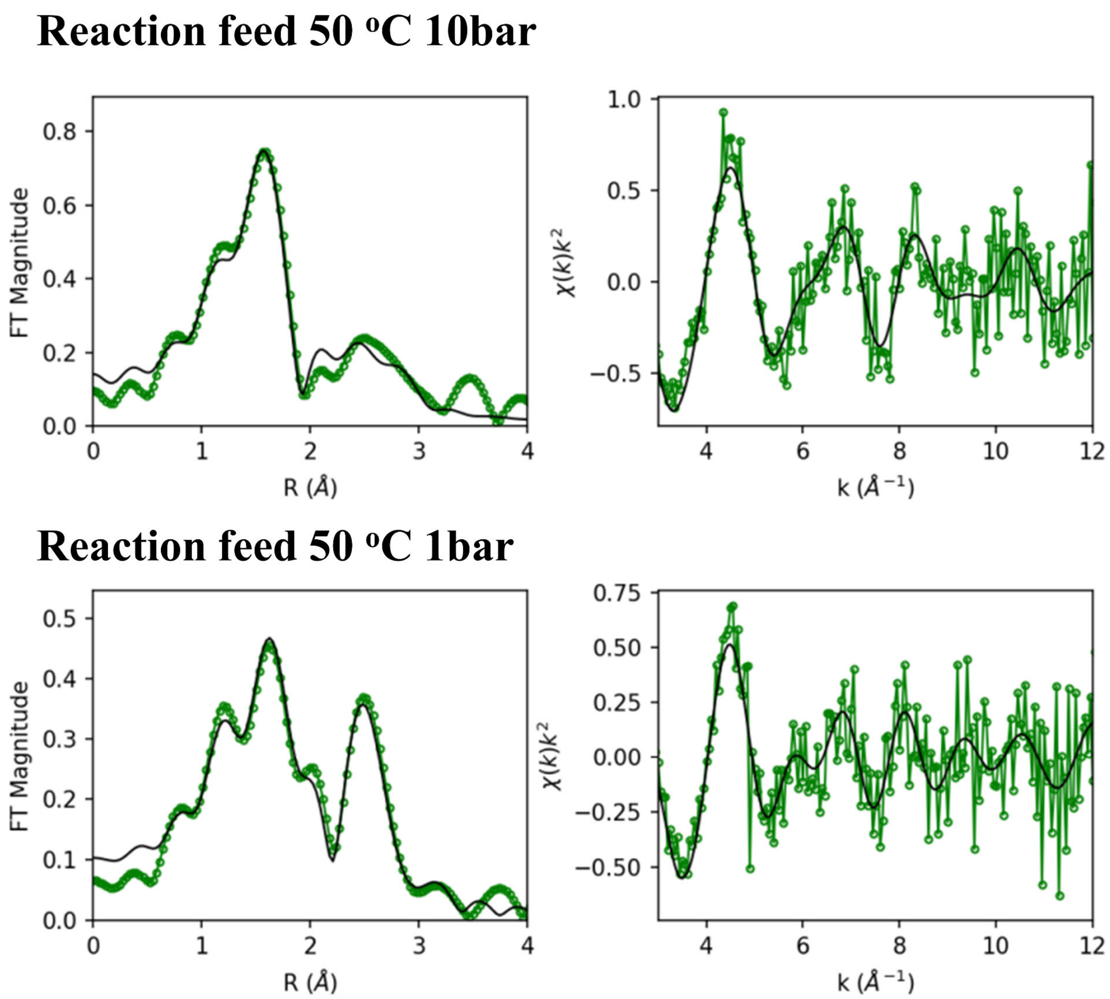

Figure S14. EXAFS fit of Rh-MFI-cal reacted at 50 ºC and 10 and 1 bar presented in Table S5.

Table S6. Multi (k^1, k^2, k^3)-weighted fits carried out in r-space (1-3.2 Å) over a k-range of 3-12 $Å^{-1}$ using a Hannings window (dk 1), and $S_0^2 = 0.9$. Bond distances and disorder parameters (Δr_{eff} and σ^2) were allowed to float having initial values of 0.0 Å and 0.003 $Å^2$ respectively, with a universal E_0 and $\Delta E_0 = 0$ eV.

Rh-MFI-cal reaction temperature 50 ºC 114min

	**R_{FACTOR}**	**X^2_v**	**Var. No.**	**k-range ($Å^{-1}$)**	**r-range (Å)**	**ΔE_0 (eV)**
	0.013	1.6	9	3-12	1-3.2	-6.0(2.9)
**PATHS**						
	**N**		**r (Å)**		**σ^2 (x10^3 $Å^2$)**	
**Rh-C/N/O**	2.0		1.84(0.02)		3.0(0.4)	
	**N**		**r (Å)**		**σ^2 (x10^3 $Å^2$)**	

	3.0	2.00(0.02)	3.4(0.6)
**Rh-Rh**	**N**	**r (Å)**	**σ^2 (x10^3 Å^2)**
	0.8	2.61(0.03)	4.9(2.3)
	**N**	**r (Å)**	**σ^2 (x10^3 Å^2)**
	0.8	2.73(0.03)	4.9(2.3)
	**N**	**r (Å)**	**σ^2 (x10^3 Å^2)**
	1.0	3.06(0.02)	3.8(1.2)
**Rh-O**	**N**	**r (Å)**	**σ^2 (x10^3 Å^2)**
	0.2	2.88(0.02)	3.0(0.4)
**Rh-O-C**	**N**	**r (Å)**	**σ^2 (x10^3 Å^2)**
	0.5	2.88(0.02)	3.0(0.4)
**Rh-C-O-C**	**N**	**r (Å)**	**σ^2 (x10^3 Å^2)**
	0.2	2.88(0.02)	3.0(0.4)

Rh-MFI-cal reaction temperature 90 °C 107min

	**R_{FACTOR}**	**X^2_v**	**Var. No.**	**k-range (Å^{-1})**	**r-range (Å)**	**ΔE_0 (eV)**
	0.018	2.0	9	3-12	1-3.2	-3.6(3.2)
**PATHS**						
**Rh-C/N/O**	**N**		**r (Å)**		**σ^2 (x10^3 Å^2)**	
	2.0		1.88(0.03)		5.4(3.2)	
	**N**		**r (Å)**		**σ^2 (x10^3 Å^2)**	
	2.0		2.03(0.04)		6.7(2.7)	
**Rh-Rh**	**N**		**r (Å)**		**σ^2 (x10^3 Å^2)**	
	2.0		2.71(0.02)		6.1(1.3)	
	**N**		**r (Å)**		**σ^2 (x10^3 Å^2)**	
	0.8		2.82(0.02)		6.1(1.3)	
	**N**		**r (Å)**		**σ^2 (x10^3 Å^2)**	
	1.5		3.06(0.02)		6.5(2.1)	
**Rh-O**	**N**		**r (Å)**		**σ^2 (x10^3 Å^2)**	
	1.0		2.92(0.03)		5.4(3.2)	
**Rh-O-C**	**N**		**r (Å)**		**σ^2 (x10^3 Å^2)**	
	2.0		2.92(0.03)		5.4(3.2)	
**Rh-C-O-C**	**N**		**r (Å)**		**σ^2 (x10^3 Å^2)**	
	1.0		2.92(0.03)		5.4(3.2)	

Rh-MFI-cal reaction temperature 200 °C 238min

	R_{FACTOR}	X^2_v	Var. No.	k-range (Å^{-1})	r-range (Å)	ΔE_0 (eV)
	0.022	0.9	7	3-12	1-3.2	-3.0(1.2)

PATHS			
**Rh-C/N/O**	**N**	**r (Å)**	**σ^2 (x10^3 Å^2)**
	2.0	1.89(0.02)	5.6(2.1)
	**N**	**r (Å)**	**σ^2 (x10^3 Å^2)**
	1.0	2.07(0.04)	10.0(0.2)
**Rh-Rh**	**N**	**r (Å)**	**σ^2 (x10^3 Å^2)**
	2.0	2.70(0.01)	4.6(0.7)
	**N**	**r (Å)**	**σ^2 (x10^3 Å^2)**
	1.0	2.82(0.01)	4.6(0.7)
**Rh-O**	**N**	**r (Å)**	**σ^2 (x10^3 Å^2)**
	2.0	3.04(0.02)	5.6(2.1)
**Rh-O-C**	**N**	**r (Å)**	**σ^2 (x10^3 Å^2)**
	1.0	3.04(0.02)	5.6(2.1)
**Rh-C-O-C**	**N**	**r (Å)**	**σ^2 (x10^3 Å^2)**
	0.5	3.04(0.02)	5.6(2.1)

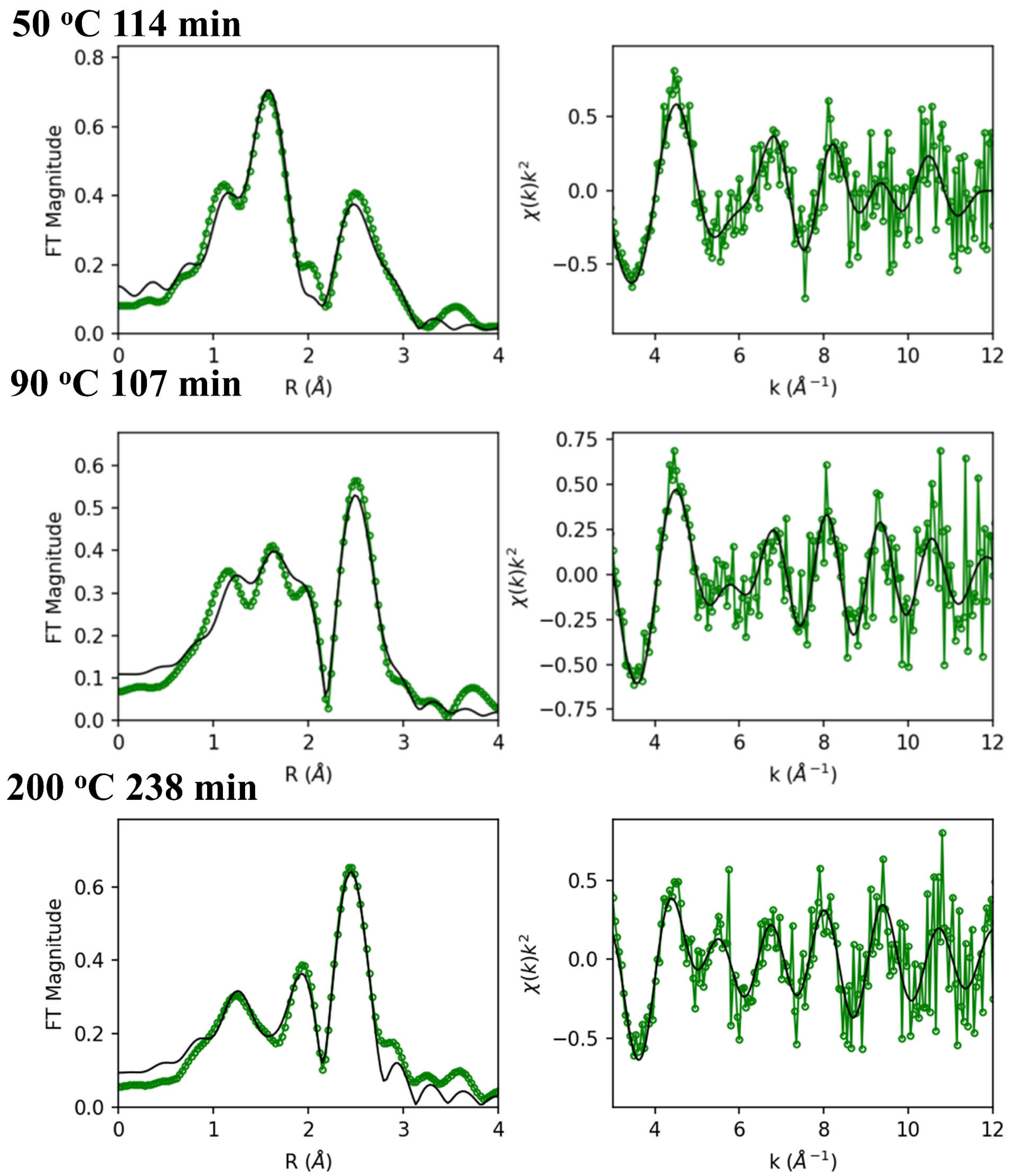

Figure S15. EXAFS fit of Rh-MFI-cal reacted at 50, 90 and 200 °C presented in Table S6.

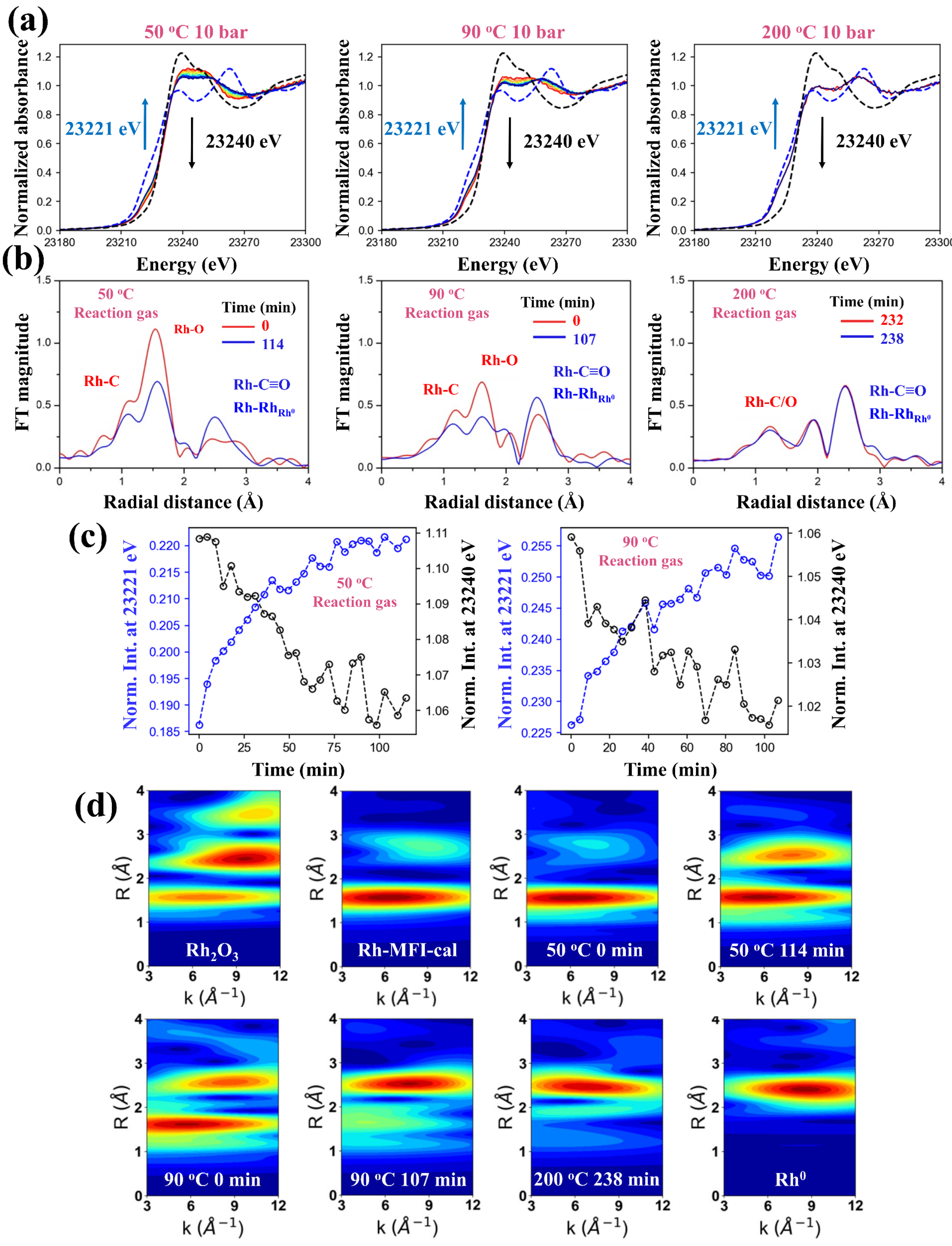

Figure S16. Effect of temperature on the XAS profile under reaction conditions: (a) XANES spectra at the Rh K-edge of samples overlaid with Rh(0) (blue dashes) and to Rh_2O_3 (black dashes); (b) Fourier transformed EXAFS signal (k^2-weigthed, Hanning window, k-range 3-12.0 $Å^{-1}$) of the first and last spectra obtained during the time resolved studies; (c) Evolution of the normalized spectral intensities at 23221 eV corresponding to Rh-CO/Rh(0) (blue) and 23240 eV

corresponding to Rh_2O_3 (black). (d) Cauchy wavelet transforms of spectra in (B) showing interference from newly generated Rh-C/O scattering in the 1-2.2 Å range.

The normalized XANES spectral intensity around 23221 eV and the peak at 1.24 Å in the FT, that becomes resolved during the reaction, are compatible with the formation of Rh-CO species[32]. By increasing the temperature under reaction conditions, the Rh_2O_3 nanoparticles are progressively converted in Rh-CO species. At 200 °C it could be that small Rh(0) clusters coexist with Rh-CO species, while Rh^{3+} sites practically disappeared.

Infrared (IR)- Mass Spectrometry (MS) studies.

The analysis of the evolution of reaction products simultaneous to the determination of the nature of active Rh species under working conditions is done in the operando IR-MS set up indicated in the experimental section. In this type of study, ethylene hydroformylation reaction is done under reaction relevant conditions, i.e. 10 bar, and in the temperature range of 50 up to 200 °C, being the reaction products analysed by online MS.

In addition to the operando studies, and in order to achieve a more detailed analysis of the nature of metal species in the working catalyst, IR-CO titration experiments are done. For that the reaction is stopped at a specific time, the sample quenched to low temperature (-65 °C) and followed by CO titration experiments.

Operando IR-MS study of Rh-MFI-cal sample in ethylene hydroformylation up to a final temperature of 90 °C

The evolution of the reaction products, i.e., propanal (m/z = 58) (in red), ethane (m/z = 30) (in blue) and propanol (m/z= 59) in turquois, with the reaction temperature and operating at 10 bar is given in Figure S17. As can be observed, propanal is the only product detected by MS remaining stable over time, in line with catalytic studies.

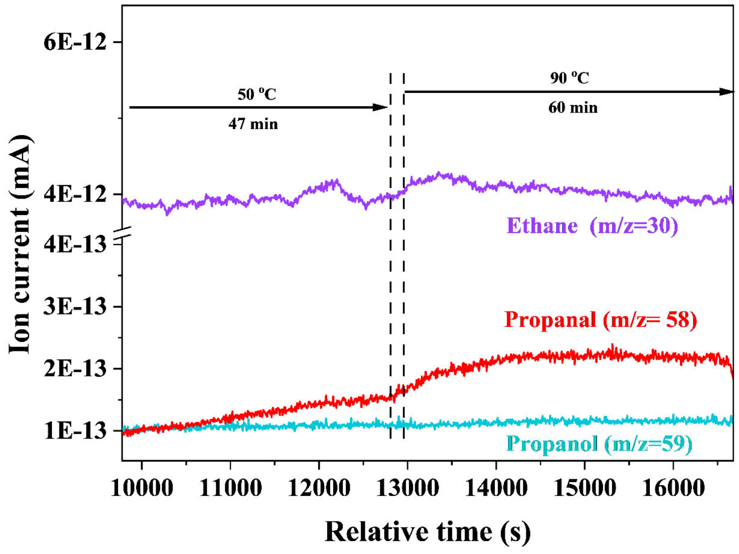

Figure S17. Evolution of reaction products based on the MS profile of the Rh-MFI-cal sample under IR reaction conditions at 50 and 90 °C, and a total pressure of 10 bar.

The IR spectra of the working catalyst is given in Figure S18. Unfortunately, the strong IR signal of the gas phase overwhelmed the region of carbonyl species in the IR spectra, impeding analysis of the Rh active site involved in propanal formation. However, a band at 1700 cm^{-1} is clearly observed from the beginning of the reaction, increasing in intensity at increasing reaction temperature up to 90 °C. This band is due to adsorbed propanal[24].

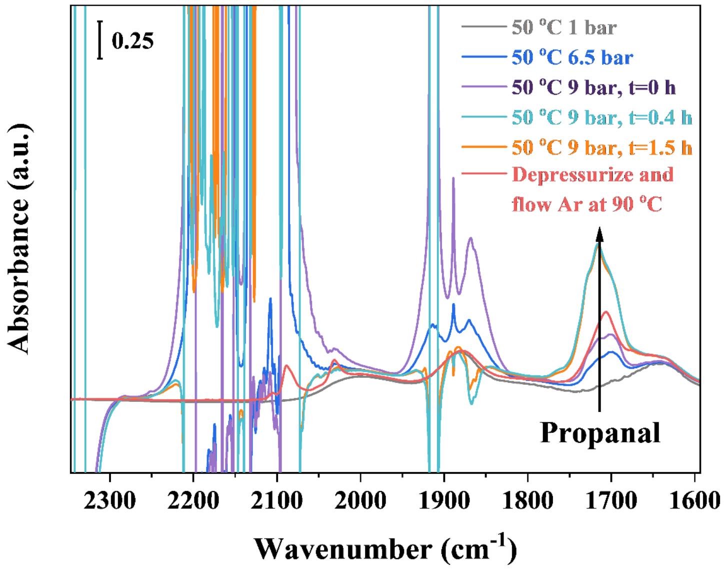

Figure S18. IR spectra of Rh-MFI-cal sample under operando hydroformylation conditions.

In order to capture reaction intermediate species, time resolved IR studies are done collecting IR spectra after fast gas pressure release at 90 °C (i.e., transient conditions) (Figure S19a). An IR band centred at 2080 cm^{-1} with a shoulder at 2087 cm^{-1} together with a small component at 2032 cm^{-1} is clearly detected (red spectra), behaving unstable in the absence of reactants. Thus, in flow of argon the IR band at 2080 cm^{-1} decrease in intensity, and a set of two IR peaks at 2090 and 2032 cm^{-1} are observed (blue spectra). Both bands correspond to the symmetric and asymmetric vibration of $Rh^+(CO)_2$ species[16].

A closer inspection to our data, shows a parallel decrease of the IR band at 2080 cm^{-1} with the one at 1700 cm^{-1} (due to the ν(C=O) of propanal) in addition to a restoring of OH groups (Figure S19b). This may indicate interaction of CO and propanal on the same active site, where the restoring of OH groups could correspond to an initial interaction of hydroxyl groups with the carbonyl group as indicated by other authors[33, 34]. The contribution of trace amounts of water in the Ar flow in the restoring of the OH groups can be discarded as shown in Figure S20. Based on the frequency of the CO mode it can be ascribed to rhodium mono-carbonyl, whose CO frequency should appear between those of the symmetric and antisymmetric modes of di-carbonyls. However, many studies in the literature tried to isolate Rh(I) mono-carbonyls but due to its instability their identification and characterization is complicated, reporting differed values[11, 35, 36]. In this direction, Rösh et al., performed a critical analysis of reported experimental data corroborated by theoretical studies and concluded that IR bands at 2076 and 2096 cm^{-1} are ascribed to mixed mono-carbonyl dihydrogen complexes ($Rh(I)H_2(CO)$ and $Rh(I)(H)_2(CO)$), respectively[37]. Based on it we may assign the IR band at 2080 cm^{-1} to a Rh(CO)L complex (L=propionyl) (see Figure S21), which appears as intermediate specie involved in the reaction path for propanal formation. A change in the oxidation state of Rh from 1+ to 3+ and vice versa has been reported in homogeneous catalysts under reaction conditions[34]. However, the involvement of Rh^{3+} species is difficult to determine experimentally since quantitative analysis of Rh^{3+} by IR-CO is challenge, because Rh^{3+} is unstable in presence of CO even at low temperature (see Appendix 1), being quickly reduced to Rh^+. In fact, after pressure release, when the reactant gas is switched to Ar flow, $Rh^+(CO)_2$ species are clearly formed at expense of the original 2080 cm^{-1} band of the Rh(CO)L intermediate (Figure S19a). That is due to the reducing power of remaining CO present in the catalytic cell. However, after low temperature CO titration (at -65 °C) an IR bands at 2136 cm^{-1} associated to Rh^{3+} is clearly detected together with the initially present IR bands at 2090 and 2032 cm^{-1} due to $Rh(I)(CO)_2$ (Figure S19).

The disruption of Rh_2O_3 to Rh^{3+} species and/or to the intermediate stable Rh(CO)L specie can be followed in the operando IR-MS studies. In fact, the formation of CO_2 (m/z = 44) and H_2O (m/z

= 18) in the initial state of the reaction at 50 °C once the pressure has reached 10 bar, is clearly seen by mass spectrometry in the Rh-MFI-cal sample (Figure S23a and b), while not observed in the Rh-MFI-calred sample (Figure S23c) nor Rh/MFI impregnated calcined reduced sample (Figure S23d). The formation of CO_2 and H_2O may come from the reactant induced fragmentation of Rh_2O_3 clusters into single sites. Notice that propanal is immediately detected by mass spectrometry and is also detected in the IR spectra, indicating a fast fragmentation and interaction of Rh sites with the reactants to generate the in situ Rh(CO)L intermediate species involved in propanal formation.

Additionally, H_2O and CO_2 are also detected in the mass spectra at higher temperature (135 ºC), paralleling the formation of ethane, and being much more intense in the Rh-MFI-cal sample. This may come from the reaction of CO and H_2 with OH or O groups of the zeolite. Because the simultaneous formation of ethane which indicate sintering of single sites into metal clusters, we suggest that those O or OH groups have been participated in the stabilization of the Rh^{3+} site. Hence, from this result we can conclude in a strong interaction and stabilization of Rh^{3+} sites by silanols groups of the zeolite.

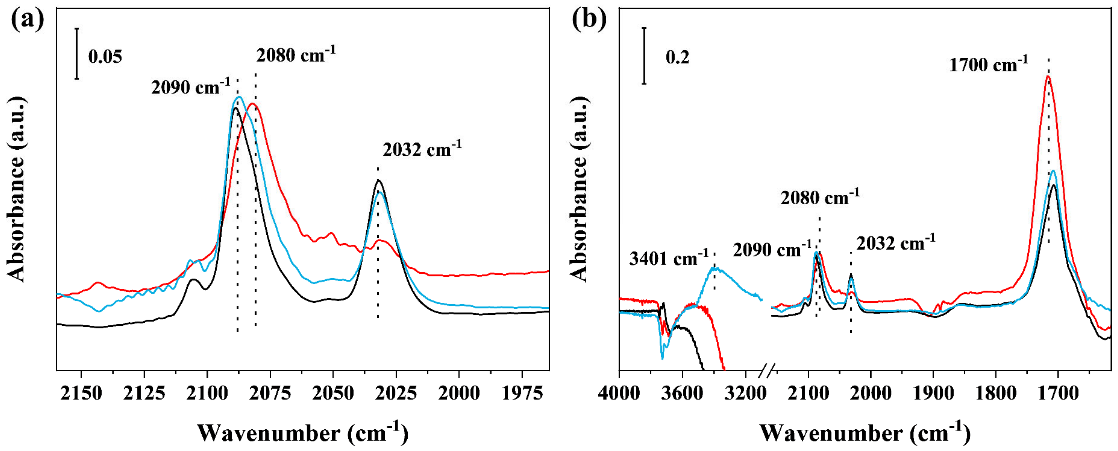

Figure S19. (a) IR spectra after de-pressurization (10 bar→1bar) of the spectroscopic cell at the reaction temperature, 90 ºC, (red line) and further purging in argon flow (blue and black line); (b) extended IR spectra in the 2200-1600 cm^{-1} range.

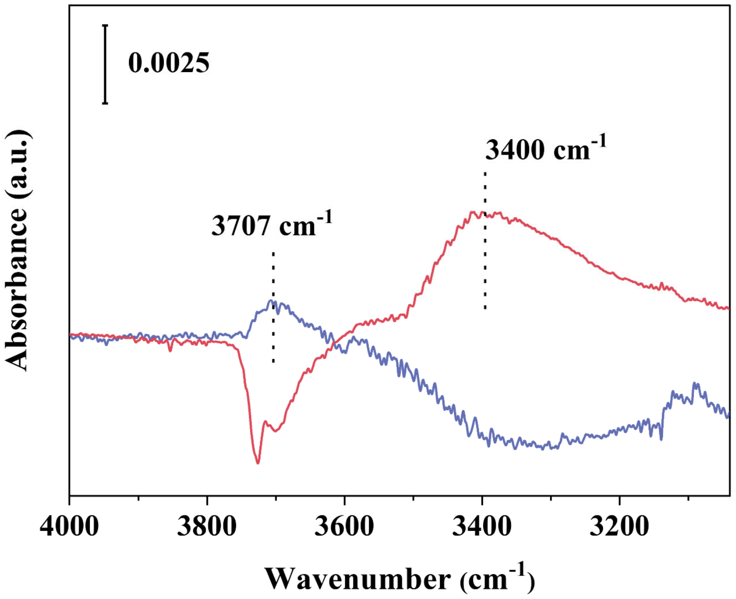

Figure S20. IR spectra of pure MFI zeolite (blue line) compared to that of the Rh-MFI-cal sample (red line) under Argon flow after de-pressurization (10 bar → 1bar) of the spectroscopic cell at the reaction temperature of 90 °C.

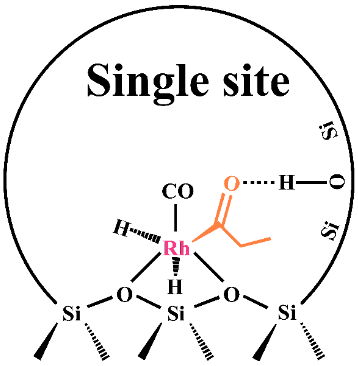

Figure S21. Proposed intermediate Rh(CO)L complex (L=propionyl, i.e., $CH_3CH_2CO^*$) as identified from operando IR spectroscopic study.

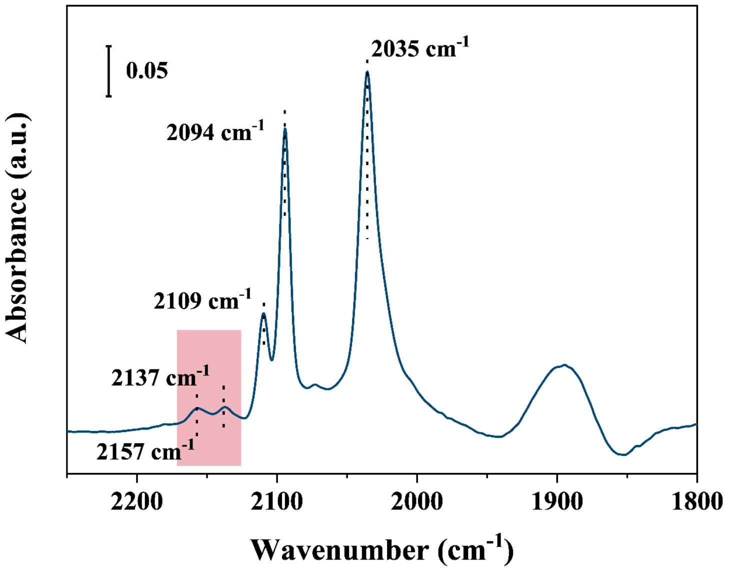

Figure S22. IR-CO at -65 °C of the Rh-MFI-cal sample after in situ hydroformylation reaction at 90 °C and 10 bar.

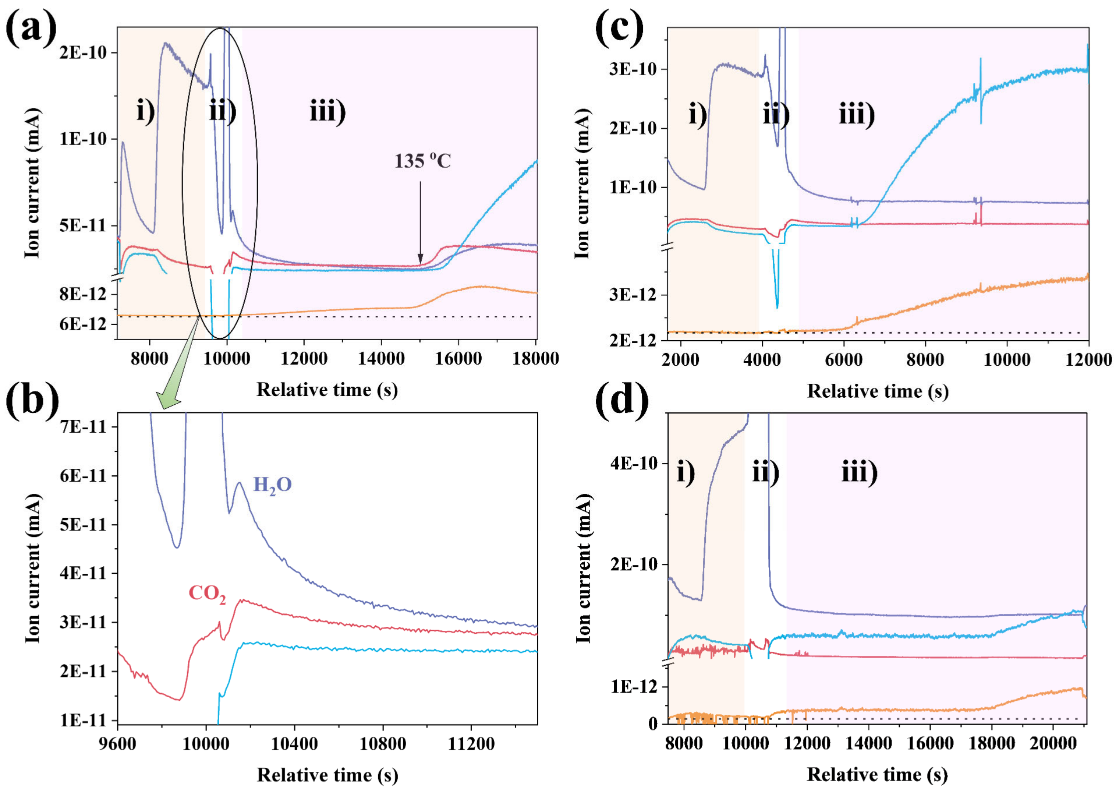

Figure S23. Mass spectra monitoring the evolution of CO_2 (red line) and H_2O (violet line) during operando IR-MS conditions at different stages: i) 50 ºC and 1bar, ii) pressure increase at 50 ºC from 1 to 10 bar, iii) stabilization at 10 bar and 50 ºC and further increasing temperature up to 200 ºC, for (a) Rh-MFI-cal, (b) inset of Rh-MFI-cal market with a circle, (c) Rh-MFI-calred

and (d) Rh/MFI impregnated samples. Other products displayed in the mass spectra are ethane, shown as a blue line and propanal as a brown line. Notice that H_2O is detected in all samples at 50 ºC and 1bar which may come from the zeolite.

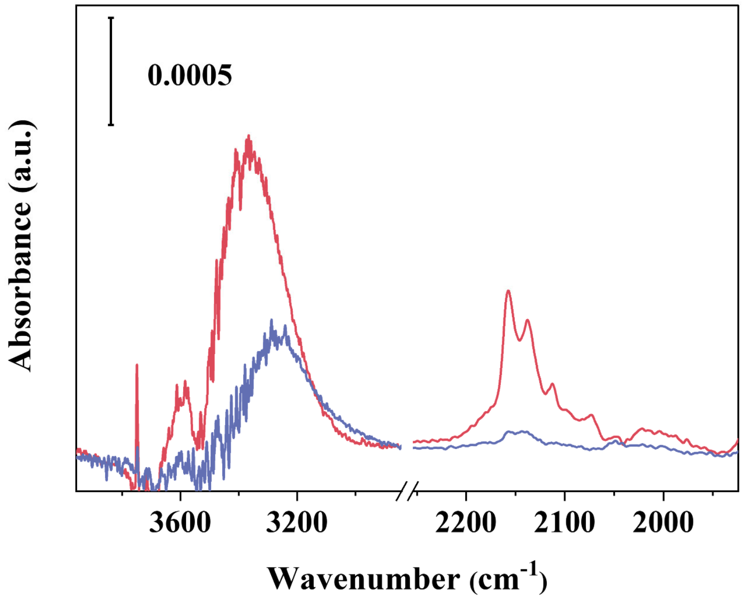

Figure S24. IR-CO at -65 ºC and at saturation CO coverage (2 mbar) for Rh-MFI-cal (red line) and pure MFI (violet line) samples, in order to present the contribution of CO coordinated to silanol groups (2156 cm^{-1}) and physisorbed CO (2135 cm^{-1}) overlapped by the Rh^{3+}-CO band.

Operando IR-MS study of Rh-MFI-cal sample in ethylene hydroformylation up to a final temperature of 200 °C

The evolution of the reaction products, i.e., propanal (m/z = 58) (in red), ethane (m/z = 30) (in purple) and propanol (m/z = 59) (in turquois) at increasing the reaction temperature from 50 to 200 °C at a constant pressure of 10 bar is given in Figure S25.

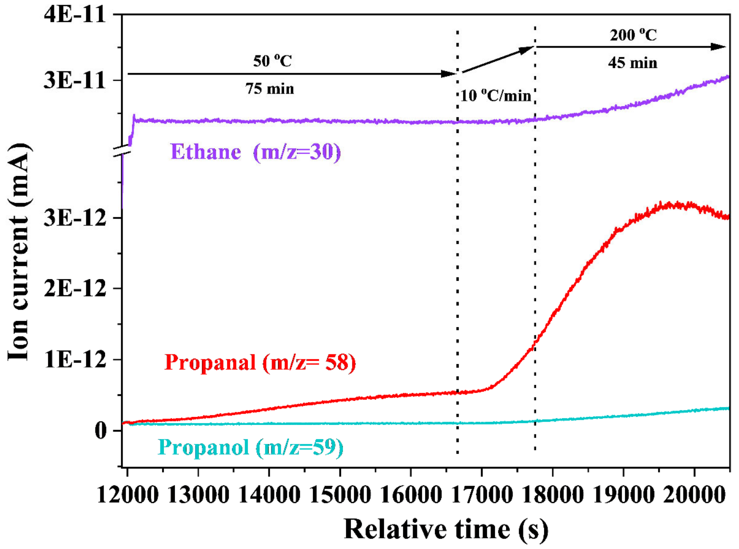

Figure S25. Evolution of reaction products based on the MS profile of the Rh-MFI-cal sample under IR reaction conditions from 50 to 200 °C, and a total pressure of 10 bar.

As can be seen, propanal formation start already at 50 °C while ethane is formed later with an onset temperature of 140 °C.

Next, in order to capture reaction intermediate species, time resolved IR studies are done collecting IR spectra after fast gas pressure release at 200 °C (i.e., transient conditions). After depressurization, an IR band centred at 2080 cm^{-1} with a shoulder at 2087 cm^{-1} together with a small component at 2032 cm^{-1} is clearly detected (Figure S26a, red line), behaving unstable in the absence of reactants. Thus, in flow of argon the IR band at 2080 cm^{-1} decrease in intensity, and only after complete disappearance the previous non detected IR bands of CO becomes visible. A closer inspection to our data, shows a parallel decrease of the IR band at 2080 cm^{-1} with the one at 1700 cm^{-1} (due to the ν(C=O) of propanal) in addition to a restoring of OH groups (Figure S26b). Similar behaviour has been observed previously when stopping the reaction at 90 °C, and, as before, the band at 2080 cm^{-1} is ascribed to a Rh (CO)L complex (L=propanal), which appears as intermediate specie involved in the reaction path for propanal formation.

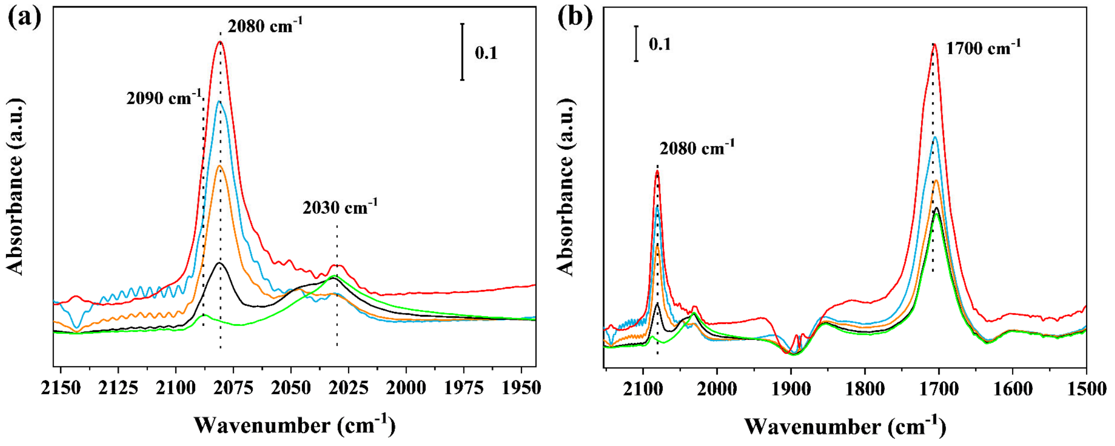

Figure S26. (a) IR spectra after de-pressurization (10bar→1bar) of the spectroscopic cell at the reaction temperature, 200 °C, (red line) and further purging in argon flow (from blue to green lines); (b) extended IR spectra in the 2200-1600 cm^{-1} range.

Next, IR-CO titration of the catalyst after having been submitted to reaction conditions at 200°C, shows a complex pattern including Rh^{3+} (2135cm^{-1}), $Rh(I)(CO)_2$ (2090 and 2031 cm^{-1}), and Rh clusters (2050 and 1891 cm^{-1})[24] (Figure S27).

The coexistence of single sites and metal clusters in the catalyst after having been submitted to 200 °C is supported by STEM analysis (Figure S4c) and XAS data (Figure S16).

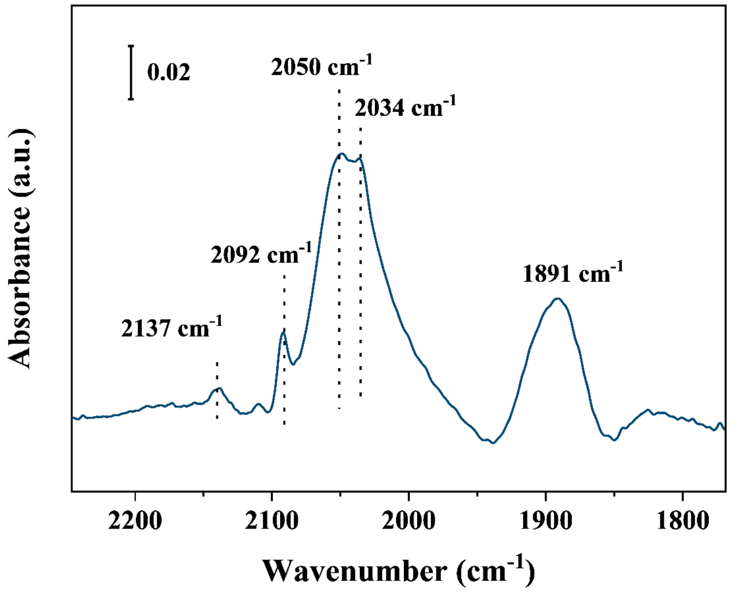

Figure S27. IR-CO at -65 °C of the Rh-MFI-cal sample after in situ hydroformylation reaction at 200 °C and 10 bar.

3.2 Catalytic study of a pre-activated Rh-MFI calcined sample in syngas flow

3.2.1 Spectroscopic IR-MS study

Calcined Rh-MFI catalyst is in situ pre-activated in 22 mL/min flow of CO/H_2 (1:1 molar) at 120 °C for 1h, resulting in the formation of $Rh(I)(CO)_2$ species (Figure S28).

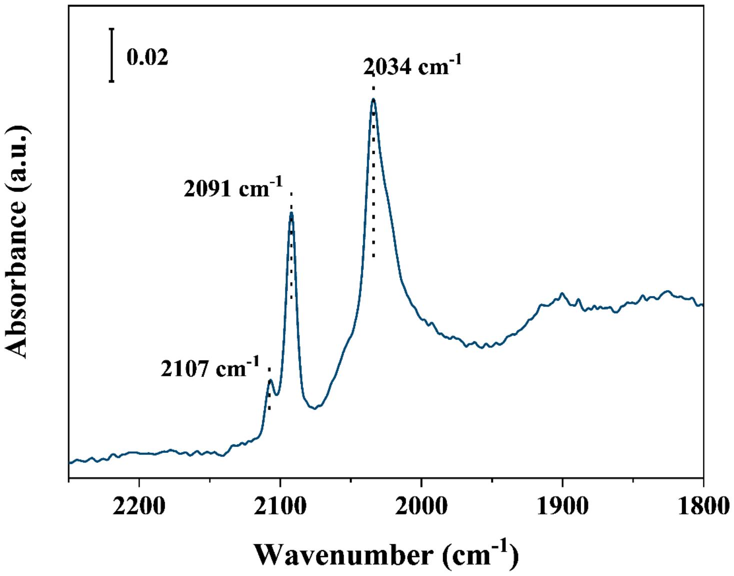

Figure S28. IR spectra of CO adsorption at -65 °C on the pre-activated Rh-MFI-cal sample in syngas flow at 120 °C for 1h.

The evolution of the reaction products, in the operando IR-MS study, is shown in Figure S29. Negligible propanal formation is formed at low temperature, i.e. 50 °C, on the syngas pre-activated sample containing $Rh(I)(CO)_2$ (red line, Figure S29c), compared to the non-activated Rh-MFI-cal sample (black line, Figure S29c). Moreover, the onset temperature of propanal formation is at 104 °C on the pre-activated sample containing $Rh(I)(CO)_2$ (Figure S29b), whereas in the calcined sample it is at 50 °C (Figure S29a). All these point to a lower intrinsic activity of $Rh(I)(CO)_2$ species.

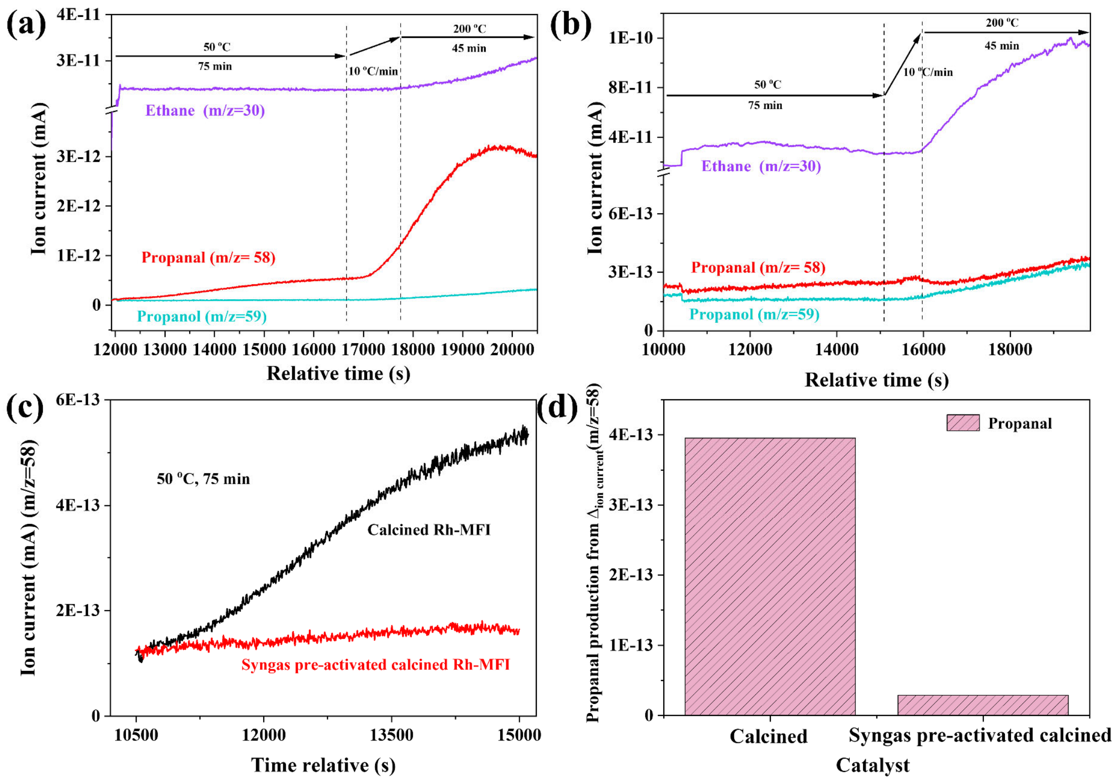

Figure S29. Evolution of reaction products based on the MS profile on Rh-MFI-cal (a) and syngas pre-activated Rh-MFI-cal sample (b) during operando IR-MS hydroformylation reaction at 10 bar and at increasing temperature from 25 to 200 °C. (c) Mass signal associated to propanal formation MS (m/z = 58) at 50 °C on the calcined (black line) and syngas pre-activated (red line) Rh-MFI samples. (d) Comparative values of propanal MS formation at 50 °C on both catalysts.

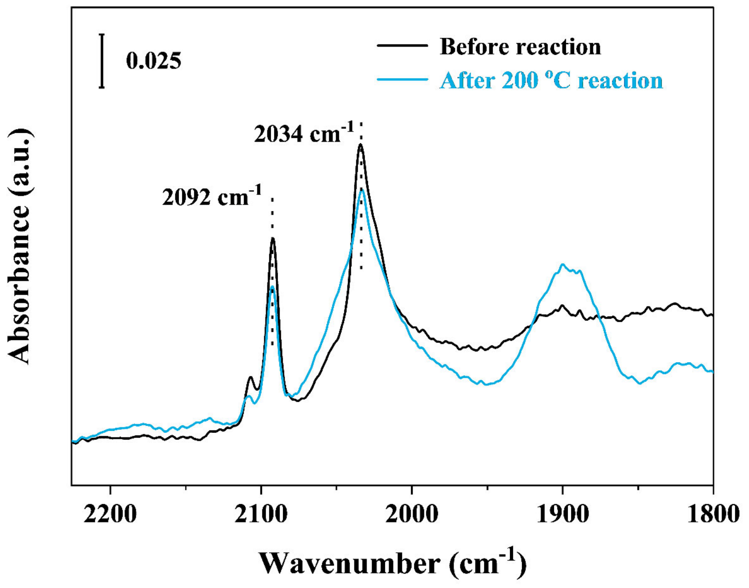

Figure S30. IR spectra of CO adsorption at -65 °C on the pre-activated Rh-MFI-cal sample in syngas flow, prior to reaction (black line) and after hydroformylation reaction at 200 °C and 10 bar (blue line).

3.2.2 Catalytic data in a fixed bed reactor

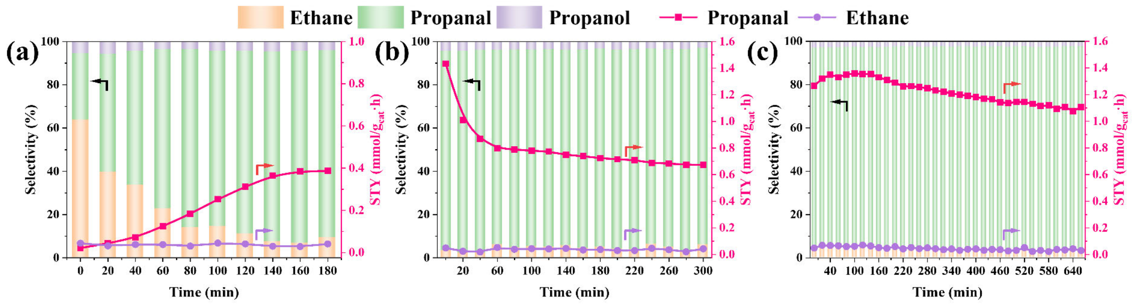

Figure S31. Catalytic performance at 50 °C (a), 70 °C (b) and 90 °C (c) and 10 bar. Right axe: space time yield (STY in mmol/g_{cat}.h) to propanal (red line) and ethane (violet line); Left axe: selectivity to products (%): propanal (marked as green column), ethane (brown) and propanol (violet). *Reaction conditions*: 100 mg catalyst diluted in 300 mg SiC. 35 mL/min total flow. GHSV 8000 h^{-1} (Ethylene: CO: H_2: N_2= 1: 1: 1: 0.5).

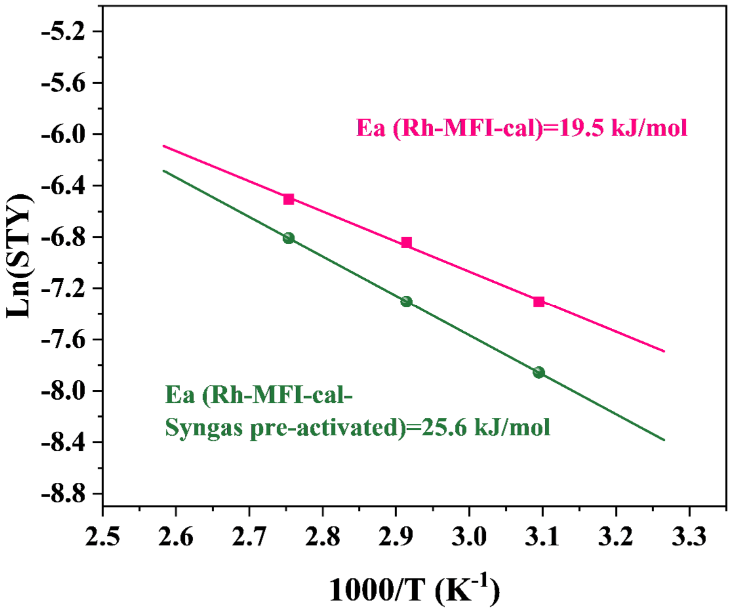

Figure S32. Arrhenius plot based on reaction rates of ethylene hydroformylation to propanal on Rh-MFI-cal (red), and Rh-MFI-cal sample pre-activated in syngas (green line), in the temperature range 50-90 °C.

3.3 Calcined reduced Rh-MFI sample

The calcined Rh-MFI sample is reduced in H_2 with heating rate of 10 °C/min to 600 °C and maintain for 3h. The obtained sample was named Rh-MFI-calred.

3.3.1 Characterization

Electron Microscopy

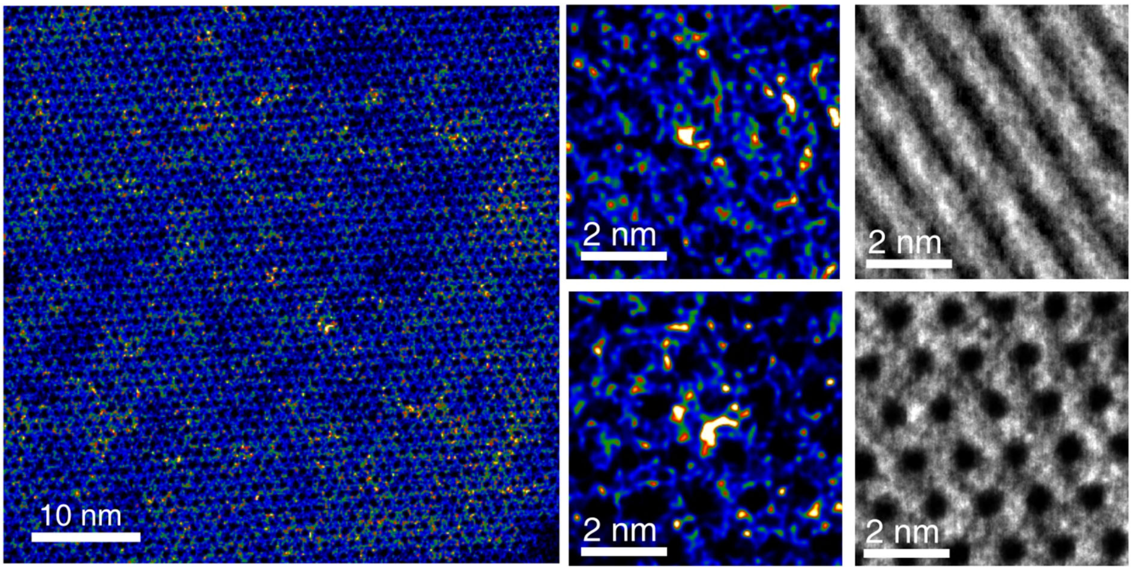

Figure S33. HAADF-STEM and iDPC images of Rh-MFI-calred sample

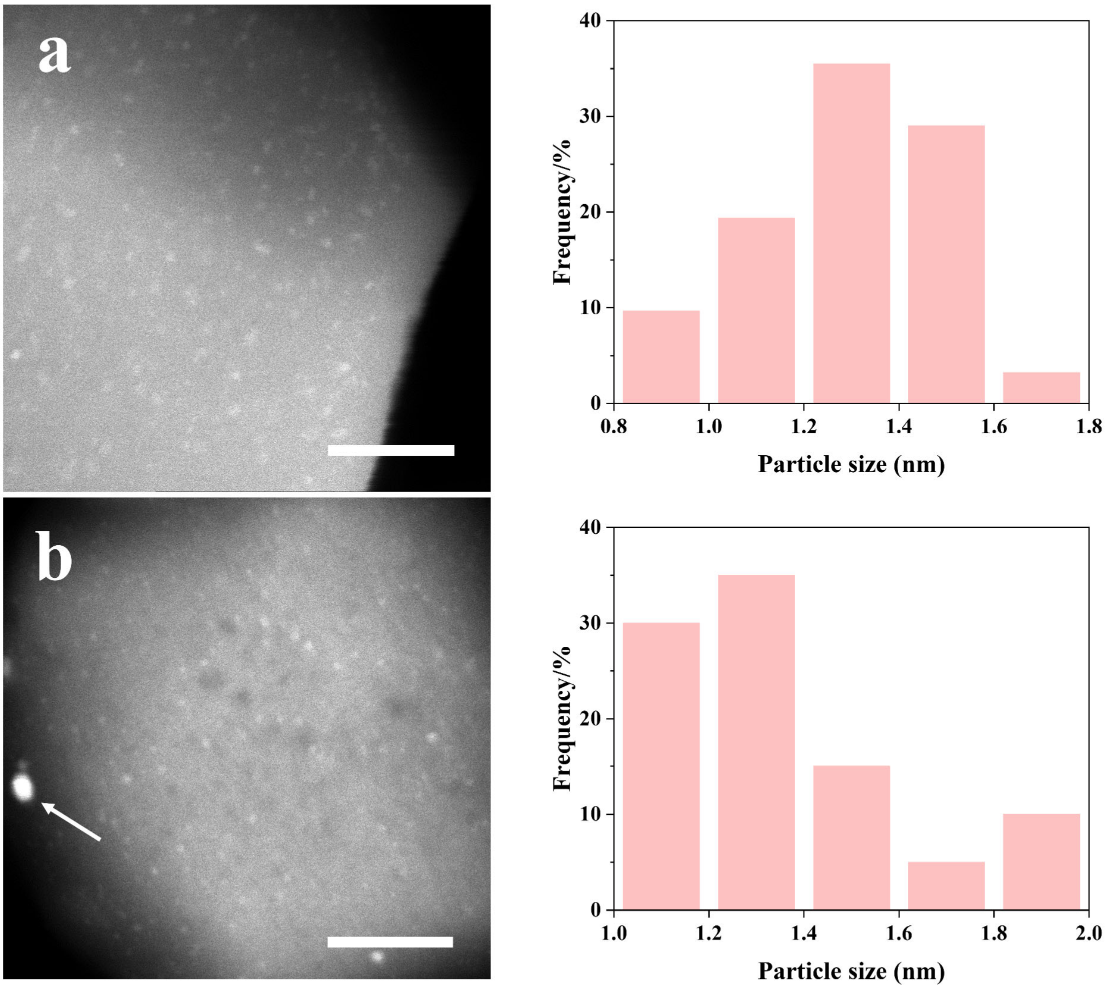

Figure S34. STEM image of (a) Rh-MFI-calred sample before reaction (i.e., fresh), (b) after hydroformylation reaction at 200 °C. Scale bars correspond to 20 nm. Particle size distribution on the right side. The average particle sizes of each sample were calculated by the following equation: $d_{TEM} = \Sigma n_i d_i^3 / \Sigma n_i d_i^2$.

IR-CO titration.

IR spectra of CO as probe molecule is shown in Figure S35. Prior to CO adsorption the sample is activated in situ in H_2 flow at 250 °C followed by evacuation in vacuum at 80 °C. CO titration experiments are done at -65 °C. A careful analysis of the experimental set up ensuring the preservation of the Rh specification in the IR experiments is given in Appendix 1.

Spectra deconvolution (Figure S35b) shows a component at 2064, 1894 and 1837 cm^{-1} corresponding to Rh^0 clusters, together with two bands at 2104 and 2024 cm^{-1} ascribed to isolated $Rh(I)(CO)_2$ species.

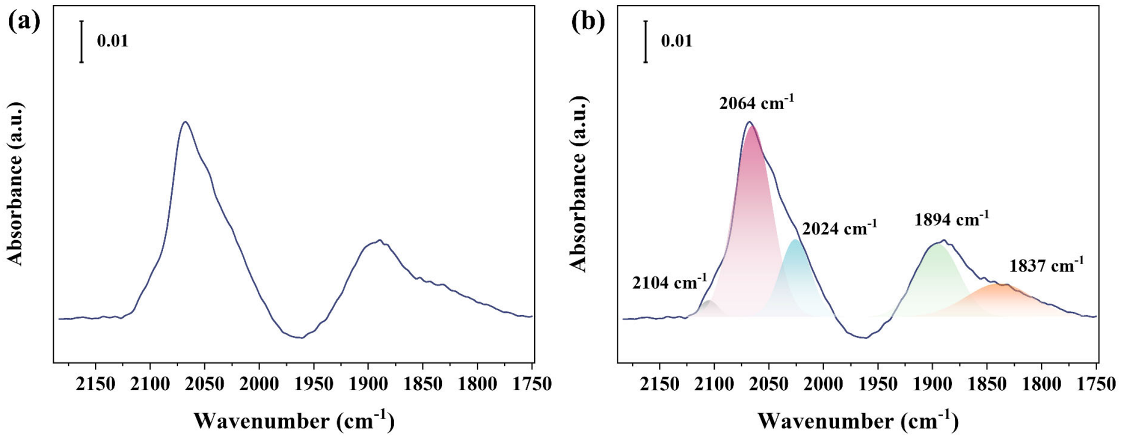

Figure S35. (a) IR of CO adsorption at -65 °C on the Rh-MFI-calred sample. (b) spectra decovolution into components.

3.3.2 Catalytic studies in a fixed bed reactor

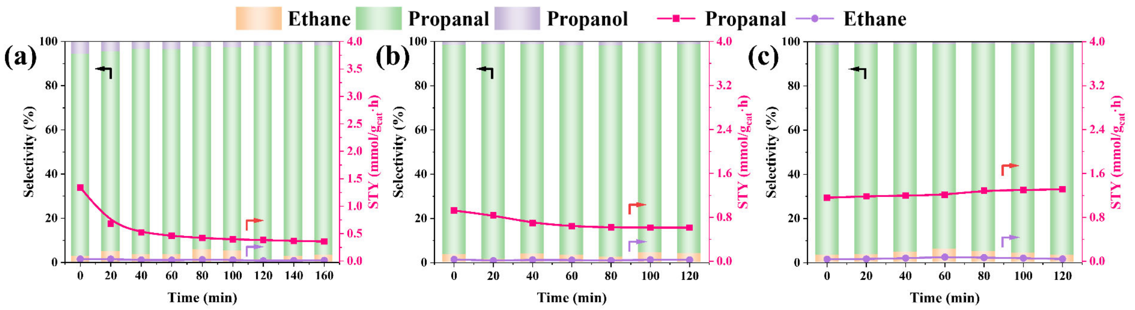

Figure S36. Catalytic performance at (a) 50 °C, (b) 70 °C and (c) 90 °C and 10 bar. Right axe: space time yield (STY in mmol/g_{cat}.h) to propanal (red line) and ethane (violet line); Left axe: selectivity to products (%): propanal (marked as green column), ethane (brown) and propanol (violet). *Reaction conditions*: 100 mg catalyst diluted in 300 mg SiC. 35 mL/min total flow. GHSV 8000 h^{-1} (Ethylene: CO: H_2: N_2= 1: 1: 1: 0.5).

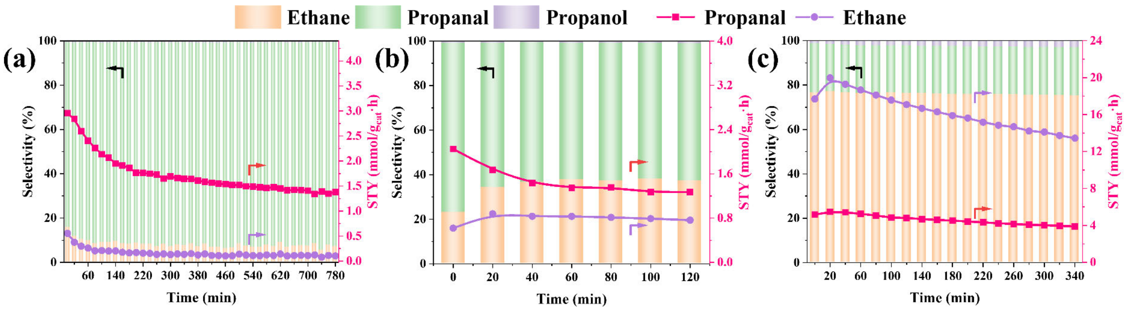

Figure S37. Catalytic performance at (a) 120 °C, (b) 150 °C and (c) 200 °C and 10 bar. Right axe: space time yield (STY in mmol/g_{cat}.h) to propanal (red line) and ethane (violet line); Left axe: selectivity to products (%): propanal (marked as green column), ethane (brown) and propanol (violet). *Reaction conditions*: 100 mg catalyst diluted in 300 mg SiC. 35 mL/min total flow. GHSV 8000 h^{-1} (Ethylene: CO: H_2: N_2= 1: 1: 1: 0.5).

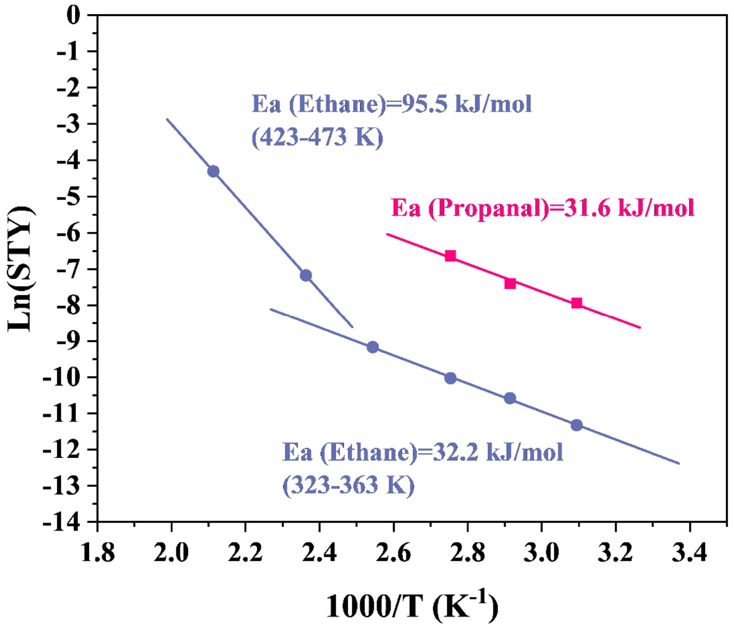

Figure S38. Apparent activation energy of Rh-MFI-calred sample. Arrhenius plot based on reaction rates of ethylene hydroformylation to propanal (red) and ethylene hydrogenation to ethane (violet) in the temperature range 50-90 °C for propanal and 120-200 °C for ethane in the Rh-MFI-calred sample. In the case of ethane, a change in the apparent activation energy is observed when moving from low (50-120 °C) to high (120-200 °C) temperature. This is because of a change in the nature of active sites as demonstrated from operando IR and XAS studies and supported by STEM, where initially single sites predominate whereas at higher temperature they tend to aggregate into metal clusters.

Table S7. Ethylene conversion and selectivity to propanal on the Rh-MFI-calred sample.

WHSV (mL/g·h)	GHSV (h^{-1})	T (°C)	Conversion(%)	Selectivity to propanal (%)	STY-Propanal (mmol/g_{cat}·h)	TOF (h^{-1})
21000 [a]	8000	50	0.32	90.77	0.68	30.46

			0.16	94.90	0.36	15.99
21000 [a]	8000	70	0.41	95.08	0.92	41.35
			0.27	94.74	0.61	27.45
21000 [a]	8000	90	0.51	95.46	1.16	51.92
			0.58	95.77	1.31	58.77
21000 [a]	8000	120 [b]	1.49	83.58	2.95	132.13
			0.63	92.32	1.38	61.54
21000 [a]	8000	150	1.13	76.21	2.05	91.74
			0.86	61.88	1.27	56.84
21000 [a]	8000	200	10.86	21.20	5.48	245.14
			7.51	21.81	3.89	174.27

[a] Without colour are initial values, while those with a green background correspond to final values

[b] Reaction duration was 13h at 120 °C, while at the others temperatures, 2-6h

3.3.3 Operando studies

X-ray adsorption spectroscopy

EXAFS analysis of Rh-MFI-calred under reduction and reacted at 50, 90 and 200 °C at 10 bar.

Table S8. Multi (k^1, k^2, k^3)-weighted fits carried out in r-space (1-3.2 Å) over a k-range of 3-12 $Å^{-1}$ using a Hannings window (dk 1), and $S_0^2 = 0.9$. Bond distances and disorder parameters (Δr_{eff} and σ^2) were allowed to float having initial values of 0.0 Å and 0.003 $Å^2$ respectively, with a universal E_0 and $\Delta E_0 = 0$ eV.

Rh-MFI-calred H_2 158min

	**R_{FACTOR}**	**X^2_v**	**Var. No.**	**k-range ($Å^{-1}$)**	**r-range (Å)**	**ΔE_0 (eV)**
	0.019	1.0	7	3-12	1-3.2	-6.2(1.0)
			**PATHS**			

**Rh-C/N/O**	**N**	**r (Å)**	**σ^2 (x10^3 Å^2)**
	1.0	1.83(0.03)	5.7(3.6)
	**N**	**r (Å)**	**σ^2 (x10^3 Å^2)**
	0.8	2.04(0.03)	3.3(3.4)
**Rh-Rh**	**N**	**r (Å)**	**σ^2 (x10^3 Å^2)**
	4.5	2.65(0.01)	10.0(0.5)

Rh-MFI-calred reaction feed 162min

	**R_{FACTOR}**	**X^2_ν**	**Var. No.**	**k-range (Å^{-1})**	**r-range (Å)**	**ΔE_0 (eV)**
	0.009	0.7	7	3-12	1-3.2	-7.0(0.6)

PATHS

**Rh-C/N/O**	**N**	**r (Å)**	**σ^2 (x10^3 Å^2)**
	0.8	1.80(0.05)	9.4(4.4)
	**N**	**r (Å)**	**σ^2 (x10^3 Å^2)**
	0.8	2.03(0.04)	12.4(5.7)
**Rh-Rh**	**N**	**r (Å)**	**σ^2 (x10^3 Å^2)**
	5.5	2.64(0.01)	11.7(0.4)

Rh-MFI-calred reaction feed 235min

	**R_{FACTOR}**	**X^2_ν**	**Var. No.**	**k-range (Å^{-1})**	**r-range (Å)**	**ΔE_0 (eV)**
	0.013	0.5	7	3-12	1-3.2	-2.7(0.9)

PATHS

**Rh-C/N/O**	**N**	**r (Å)**	**σ^2 (x10^3 Å^2)**
	2.0	1.89(0.02)	8.2(1.9)
	**N**	**r (Å)**	**σ^2 (x10^3 Å^2)**
	0.8	2.03(0.02)	3.5(2.3)
**Rh-Rh**	**N**	**r (Å)**	**σ^2 (x10^3 Å^2)**
	2.1	2.71(0.01)	6.9(0.5)
	**N**	**r (Å)**	**σ^2 (x10^3 Å^2)**
	0.5	2.82(0.01)	6.9(0.5)
**Rh-O**	**N**	**r (Å)**	**σ^2 (x10^3 Å^2)**
	2.0	3.04(0.02)	8.2(1.9)
**Rh-O-C**	**N**	**r (Å)**	**σ^2 (x10^3 Å^2)**

	1.0	3.04(0.02)	8.2(1.9)
**Rh-C-O-C**	**N**	**r (Å)**	**σ² (x10³ Å²)**
	0.5	3.04(0.02)	8.2(1.9)

Rh-MFI-calred reaction feed 339min

	**R_{FACTOR}**	**X^2_v**	**Var. No.**	**k-range ($Å^{-1}$)**	**r-range (Å)**	**ΔE_0 (eV)**
	0.010	1.2	7	3-12	1-3.2	-2.8(0.9)

PATHS

**Rh-C/N/O**	**N**	**r (Å)**	**σ² (x10³ Å²)**
	2.0	1.89(0.02)	5.9(2.2)
	**N**	**r (Å)**	**σ² (x10³ Å²)**
	1.0	2.06(0.02)	3.5(2.8)
**Rh-Rh**	**N**	**r (Å)**	**σ² (x10³ Å²)**
	3.8	2.71(0.00)	4.5(0.3)
**Rh-O**	**N**	**r (Å)**	**σ² (x10³ Å²)**
	1.0	3.04(0.02)	5.9(2.2)
**Rh-O-C**	**N**	**r (Å)**	**σ² (x10³ Å²)**
	2.0	3.04(0.02)	5.9(2.2)
**Rh-C-O-C**	**N**	**r (Å)**	**σ² (x10³ Å²)**
	1.0	3.04(0.02)	5.9(2.2)

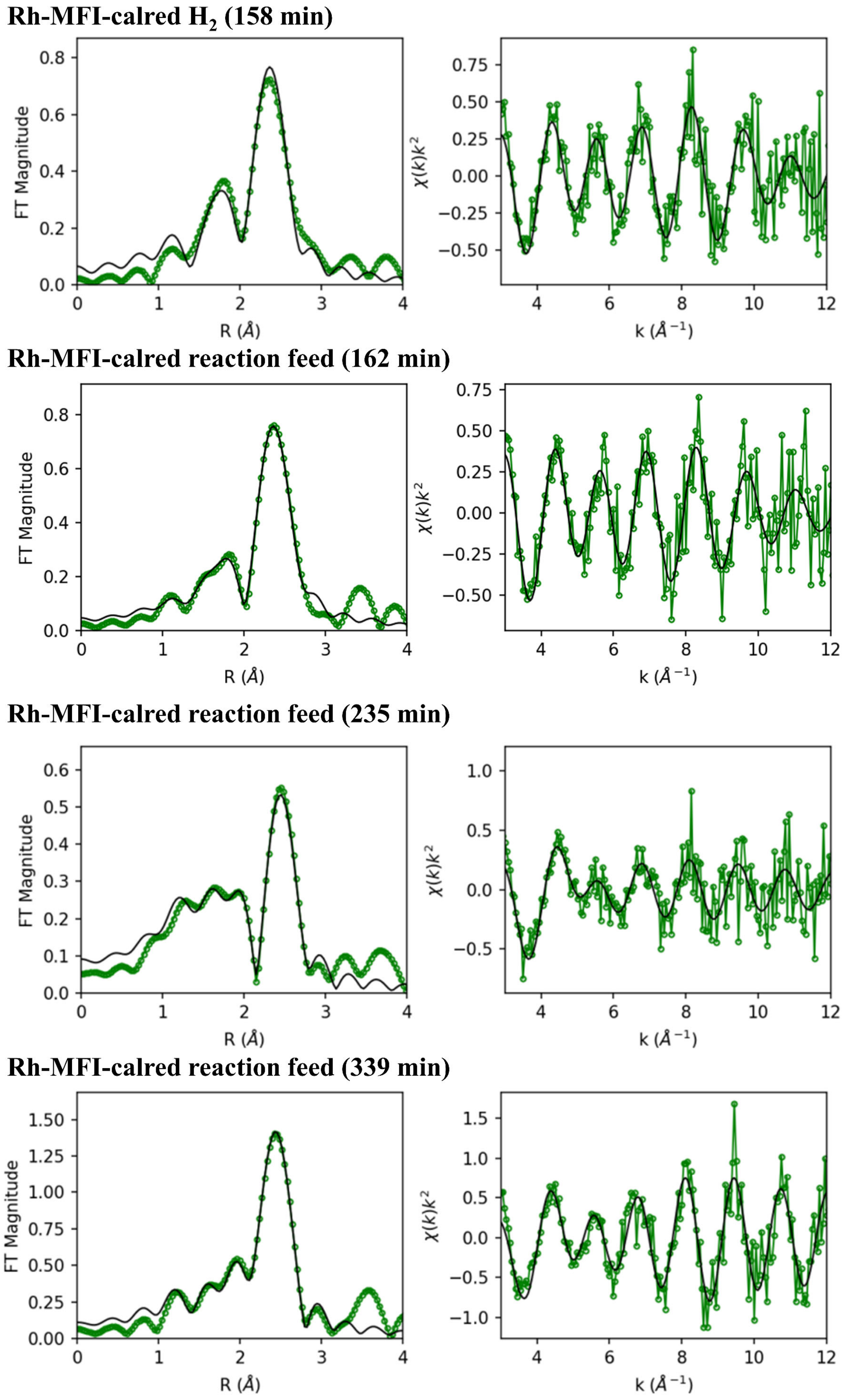

Figure S39. EXAFS fit of Rh-MFI-calred under reduction and reaction conditions at 50, 90 and 200 °C at 10 bar presented in Table S8.

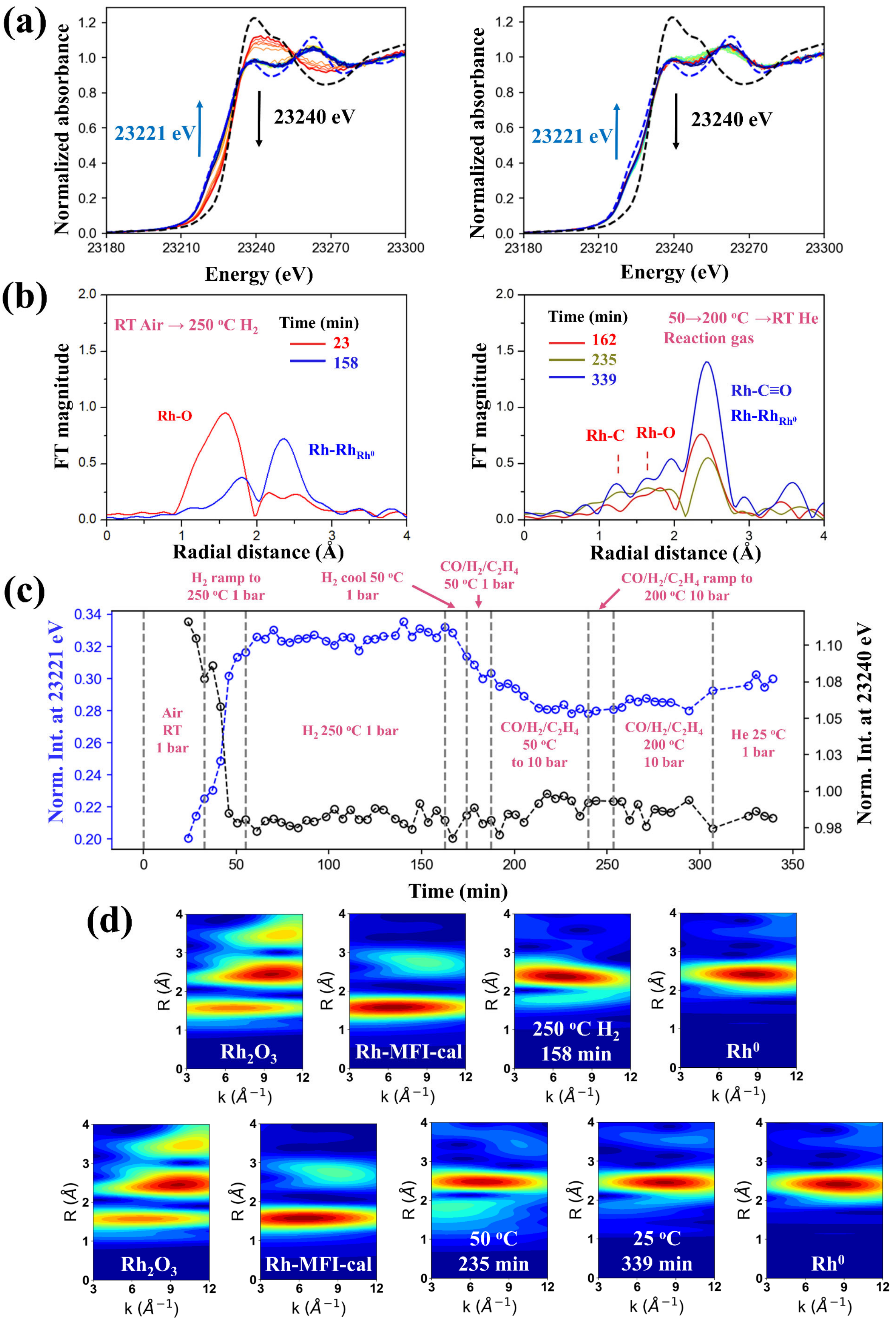

Figure S40. Effect of temperature on the XAS profile under reaction conditions: (a) Rh-MFI XANES spectra at the Rh K-edge (left) under H_2 flow at 250 °C, 1bar and 158 min; (right) under

reaction conditions at 50 °C, 10 bar over 73 min (red to green line) and further increasing temperature to 200 °C over a final period of 100 min (green to blue line). Spectra are overlaid with Rh(0) (blue dashes) and to Rh_2O_3 (black dashes); (b) Fourier transformed EXAFS signal (k^2-weigthed, Hanning window, k-range 3-12.0 Å^{-1}) showing the first and last spectrum under H_2 (left) and at key points in the reaction feed respectively (right); (c) Evolution of normalized spectral intensities at 23221 eV corresponding to Rh-CO/Rh(0) (blue) and 23240 eV corresponding to Rh_2O_3 (black). (d) Cauchy wavelet transforms of EXAFS signals shown in (b) generation of Rh-C/O interactions is clearly observed upon exposure to reaction feed (235 min) after H_2 treatment (158 min).

Initial Rh_2O_3 are converted in Rh(0) clusters by hydrogenation at 250 °C. In reaction conditions ($C_2^{=}$/CO/H_2/N_2(1/1/1/0.5 molar)) at 1 bar and 50 °C part of the Rh(0) is converted in Rh-CO phase. By increasing the pressure to 10 bar and the temperature to 200 °C the Rh(0) particles size seems to increase.

Infrared (IR)-Mass spectrometry (MS)

The Rh-MFI-calred sample has been studied in the IR-MS set up. Prior to reaction, the sample has been in situ activated in H_2 flow at 250 °C for 2h. The MS profile of the evolution of the reaction products is shown in Figure S41. Propanal (m/z = 58) (in red), ethane (m/z = 30) (in purple) and propanol (in turquois) are the predominant products. The onset temperature of propanal formation is ~100 °C, whereas for ethane is ~140 °C.

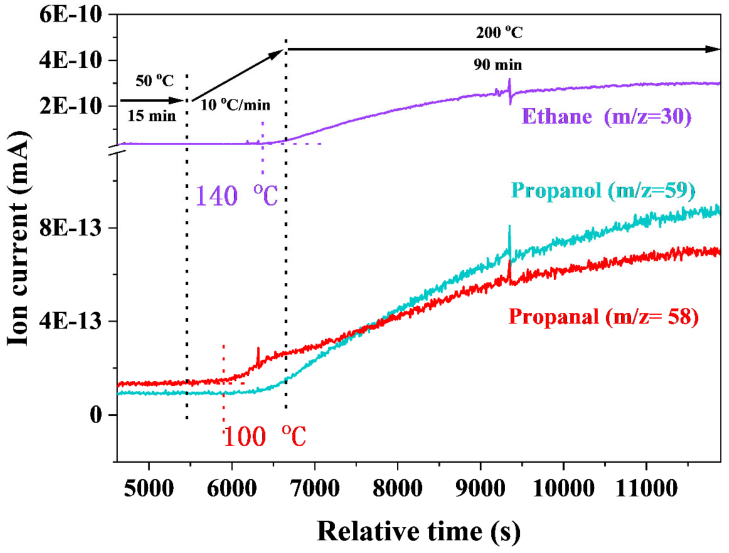

Figure S41. Evolution of reaction products based on the MS profile of the Rh-MFI-calred sample under IR reaction conditions at 50 °C and further increasing up to 200 °C (10 °C/min), and at a total pressure of 10 bar.

The in situ evolution of Rh species under reaction conditions is impossible to determine, due to the strong IR signal of the gas phase overwhelming the region of carbonyl species in the IR spectra. Under reaction conditions, a band at 1700 cm^{-1} due to propanal is clearly observed from the beginning of the reaction (Figure S42a). Next, after depressurization of the catalytic cell, at 200 °C, $Rh(I)(CO)_2$ species (2091 and 2030 cm^{-1}) are clearly observed (Figure S42b). This, together with the above reported XAS data (Figure S40) may indicate an oxidative disruption of the original Rh^0 clusters in the presence of ethylene and syngas resulting in the formation of $Rh(I)(CO)_2$ species.

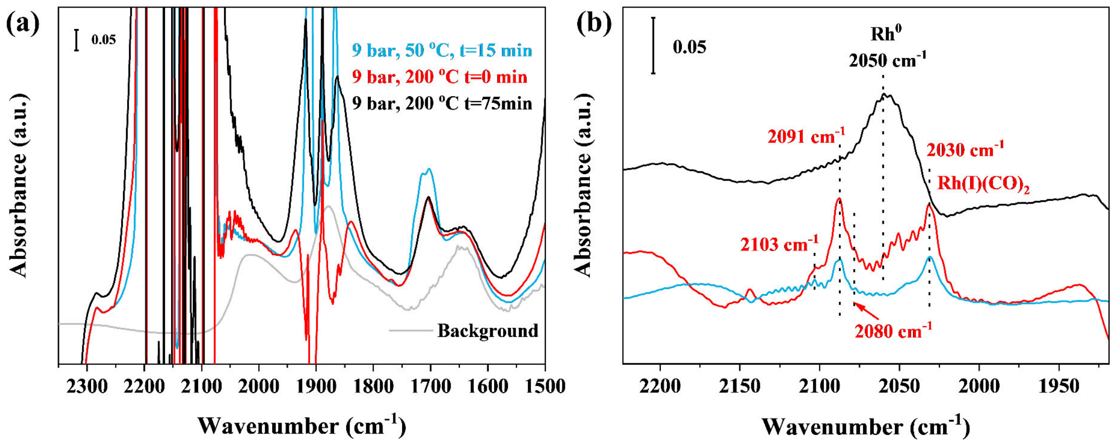

Figure S42. (a) IR spectra under operando conditions, 9 bar and 200 °C on the Rh-MFI-calred sample; (b) IR spectra of sample before reaction (black line), after pressure release at 200 °C (red line) and after argon flow at the same temperature (blue line).

Furthermore sample titration in CO at -65 °C, after having been exposed to reaction conditions at 200 °C for 90 min, shows the co-existence of various species, including $Rh(I)(CO)_2$ (2090 and 2032 cm^{-1}), and Rh clusters (2064 and 2047 cm^{-1}) (Figure S43).

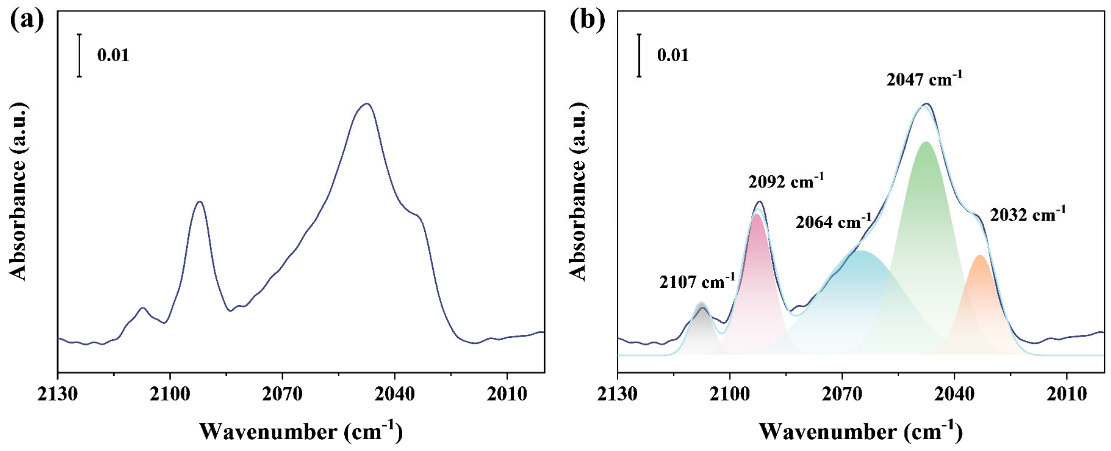

Figure S43. (a) IR of CO adsorption at -65 °C on the Rh-MFI-calred sample after IR-MS hydroformylation reaction at 200 °C and 10 bar reaction. (b) spectra deconvolution into components

3.4 Rh impregnated MFI sample

3.4.1 Synthesis

For reference catalyst, Rh was introduced by impregnation method. First, pure-Si MFI zeolite is synthesized using the same procedure described for the one-pot sample, only excluding the addition of the Rh precursor in the solution. When the synthesis is finished, the samples were calcined in a tubular oven in air (75mL/min) with a heating rate of 1.5 °C/min to 550 °C and maintain 6 hours. Then a solution of $RhCl_3$ containing required amount of Rh for a final loading of 0.3 wt% and incipient wetness amount of water was added and mixed with the solid and dried in air at 100°C to remove the physically adsorbed water. The dried sample is calcined again in a tubular oven in air (75mL/min) with a heating rate of 10 °C/min to 550 °C and maintain 3 hours. The calcined sample is named Rh/MFI-cal. Part of the Rh/MFI-cal sample was reduced in H_2 with heating rate of 1.5 °C/min to 600 °C and maintain for 3h. This prepared sample is named Rh/MFI-calred.

3.4.2 Characterisation

Microscopy

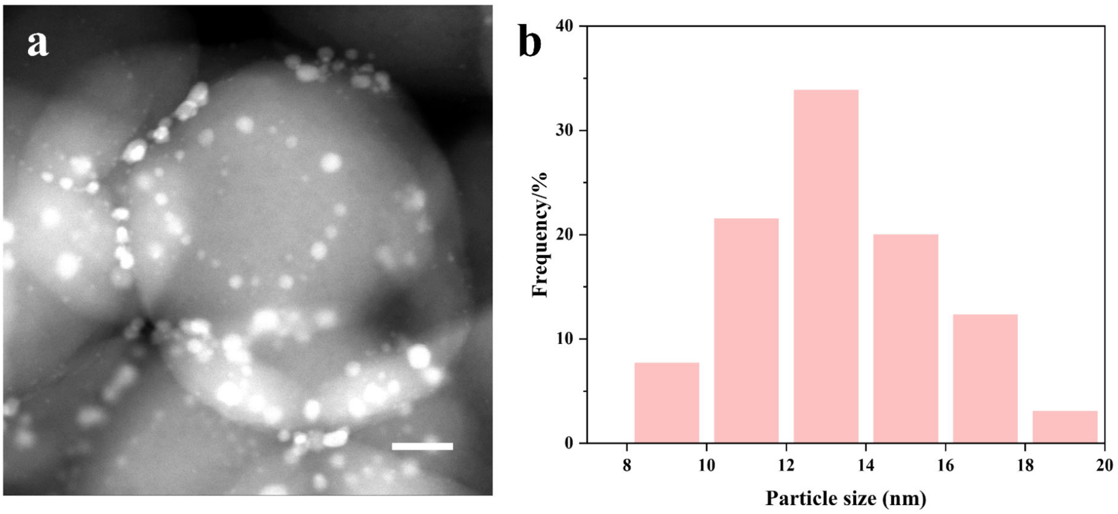

Figure S44. HAADF-STEM image (a) and particle size distribution (b) of Rh/MFI-calred sample. The scale bar corresponds to 50 nm. The average particle sizes of each sample were calculated by the following equation: $d_{TEM} = \Sigma n_i d_i^3 / \Sigma n_i d_i^2$.

3.4.3 Catalytic study

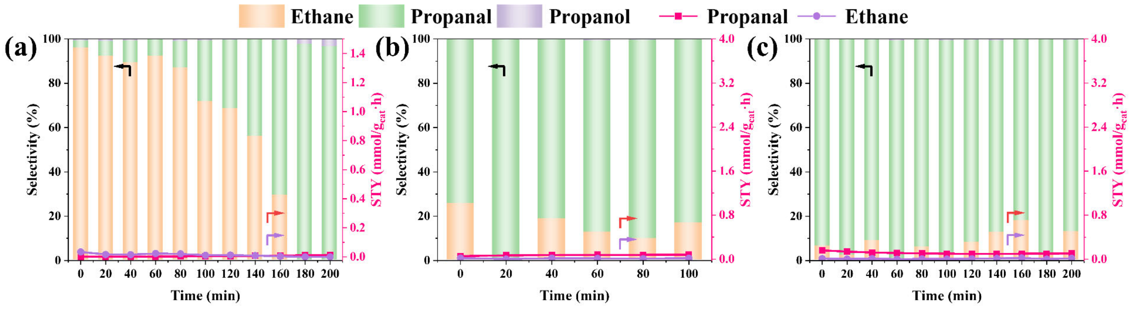

Figure S45. Catalytic performance at (a) 50 °C, (b) 70 °C and (c) 90 °C and 10 bar. Right axe: space time yield (STY in mmol/g_{cat}.h) to propanal (red line) and ethane (violet line); Left axe: selectivity to products (%): propanal (marked as green column), ethane (brown) and propanol (violet). *Reaction conditions*: 100 mg catalyst diluted in 300 mg SiC. 35 mL/min total flow. GHSV 8000 h^{-1} (Ethylene: CO: H_2: N_2= 1: 1: 1: 0.5).

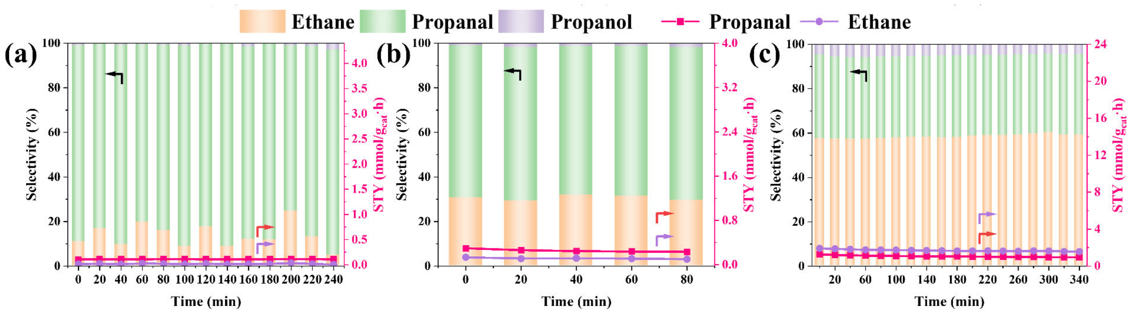

Figure S46. Catalytic performance at (a) 120 °C, (b) 150 °C and (c) 200 °C and 10 bar. Right axe: space time yield (STY in mmol/g_{cat}.h) to propanal (red line) and ethane (violet line); Left axe: selectivity to products (%): propanal (marked as green column), ethane (brown) and propanol (violet). *Reaction conditions*: 100 mg catalyst diluted in 300 mg SiC. 35 mL/min total flow. GHSV 8000 h^{-1} (Ethylene: CO: H_2: N_2= 1: 1: 1: 0.5).

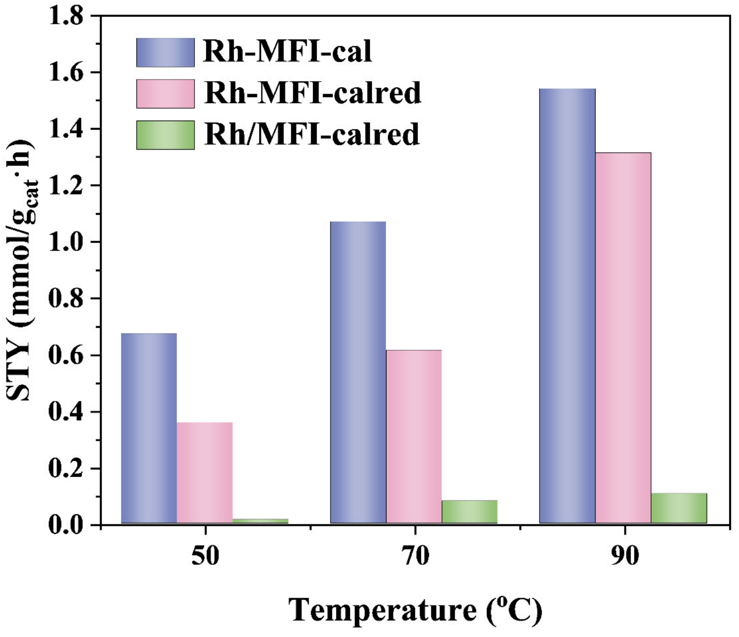

Figure S47. Propanal space time yield at 50, 70 and 90 °C over Rh-MFI-cal (violet), Rh-MFI-calred (pink) and impregnated Rh/MFI-calred (green). *Reaction conditions*: 100 mg catalyst diluted in 300 mg SiC. 35 mL/min total flow. GHSV 8000 h^{-1} (Ethylene: CO: H_2: N_2= 1: 1: 1: 0.5).

3.4.4 Operando study

Infrared (IR)-Mass spectrometry (MS)

The Rh impregnated MFI sample has been studied in the IR-MS set up. Prior to reaction, the sample has been in situ activated in H_2 at 250 °C for 2h. The MS profile of the evolution of the reaction products is shown in Figure S48. A very low activity is observed with low propanal (m/z = 58) (in red), ethane (m/z = 30) and propanol (m/z = 59) production, in line with the catalytic data. As can be seen the onset temperature of formation is around 162 °C.

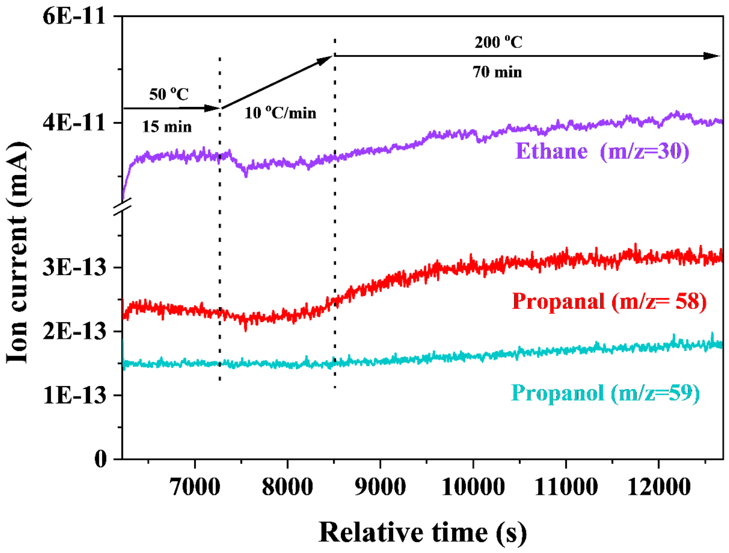

Figure S48. Evolution of reaction products based on the MS profile of the impregnated Rh/MFI-calred sample under IR reaction conditions at 50 °C and further increasing up to 200 °C (10 °C/min), and at a total pressure of 10 bar.

Next, the operando IR spectra is analysed (Figure S49). A weak band at 1700 cm^{-1} due to propanal is observed under operando conditions in line with the low activity. However, in contrast to the other samples, after depressurization of the catalytic cell no IR bands are observed. CO doesn't interact with Rh NP nor modify their particle size. Furthermore sample titration in CO at -65°C, after having been exposed to reaction conditions at 200 °C for 70 min, shows again no IR bands.

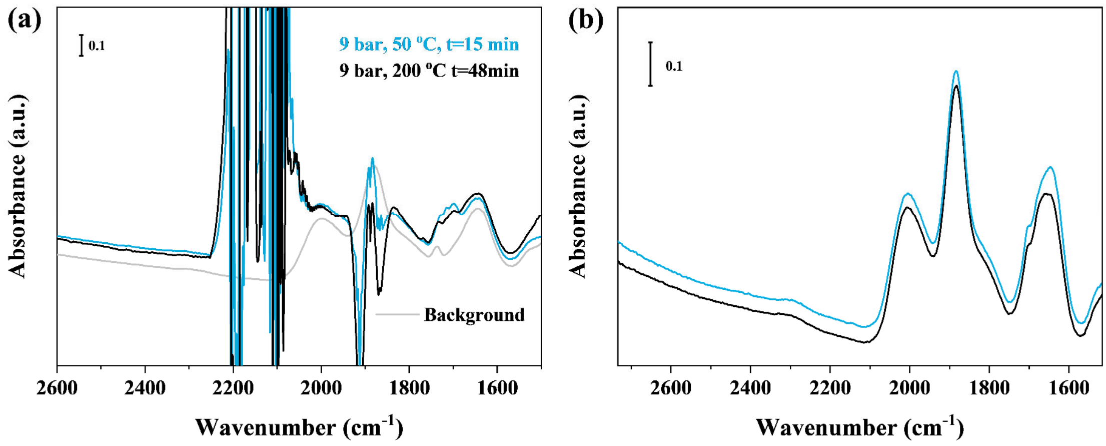

Figure S49. (a) IR spectra under operando conditions, 9 bar and 50 and 200 °C on the impregnated Rh/MFI-calred sample; (b) IR spectra of sample before reaction (black line), and after pressure release at 200 °C and CO titration at -65 °C (blue line).

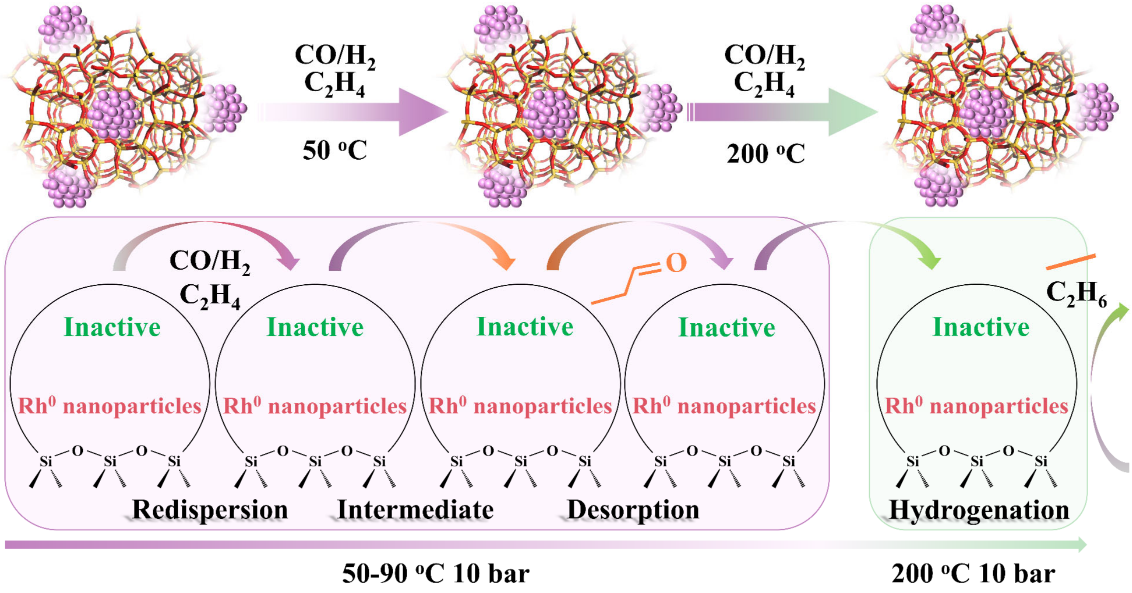

Figure S50. Behaviour of impregnated Rh/MFI-calred sample under reaction conditions containing Rh NP.

Summary of catalytic performance on Rh-MFI samples at different reaction conditions.

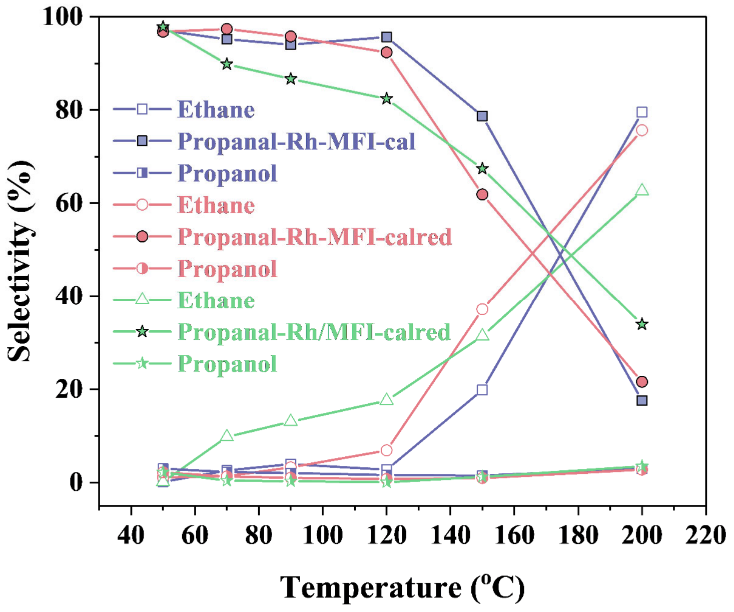

Figure S51. Variation with temperature of the selectivity to propanal (solid), ethane (hollow) and propanol (half solid) on Rh-MFI-cal (violet), Rh-MFI-calred (pink) and Rh/MFI-calred (green) samples. *Reaction conditions*: 100 mg catalyst diluted in 300 mg SiC. 35 mL/min total flow. GHSV 8000 h^{-1} (Ethylene: CO: H_2: N_2= 1: 1: 1: 0.5).

3.5 Rh-(O)-P-MFI sample

3.5.1 Synthesis

Synthesis of Rh-(O)-P-MFI was in a similar manner as Rh-MFI sample, with tetraorthoethylsilicate (TEOS) as silica source and a mixture of tetrapropylammonium hydroxide (TPAOH) and tetraethylphosphonium hydroxide (TEPOH) as organic templates. The rhodium precursor was a solution of rhodium (III) chloride hydrate and ethylenediamine (Rh-en). In a typical synthesis, the solution of rhodium precursor was prepared by dissolving 0.263 g $RhCl_3$ hydrate (38% Rh) in 90g milliQ water, when the solution became homogeneous, 10g ethylenediamine was added into the solution under stirring. The solution was kept stirring at room temperature for 16 h till a transparent light yellow solution was formed. Then, a mixture containing 9.5 g TPAOH (43.12%, exchanged from tetrapropylammonium bromide in house using anion exchange resin), 1.75 g TEPOH (9.37%) and 10 g H_2O was mixed in a plastic beaker, followed by adding of 17.36 g TEOS. Then, the beaker was sealed with parafilm and kept under stir for 2 h to allow full hydrolysis of TEOS. Afterward, the parafilm was removed and the solution was allowed to keep under stir for 16 h to evaporate ethanol generated from hydrolysis of TEOS. 5 g Rh-en solution was added into the zeolite synthesis gel and was transferred to a Teflon-lined autoclave and heated to 175 °C under agitation for 4 days for crystallization. When

the crystallization was finished, the solid product was collected by filtration and washed thoroughly with distilled water and dried in air at 100 °C.

The dried sample is calcined in a tubular oven in air (75 mL/min) with a heating rate of 1.5 °C/min to 550 °C and maintain 6 hours. The obtained sample was named Rh-(O)-P-MFI-cal. Then, it is reduced in H_2 with heating rate of 10 °C/min to 600 °C and maintain for 3h. The obtained sample was named Rh-(O)-P-MFI-calred.

3.5.2 Characterisation:

Electron Microscopy

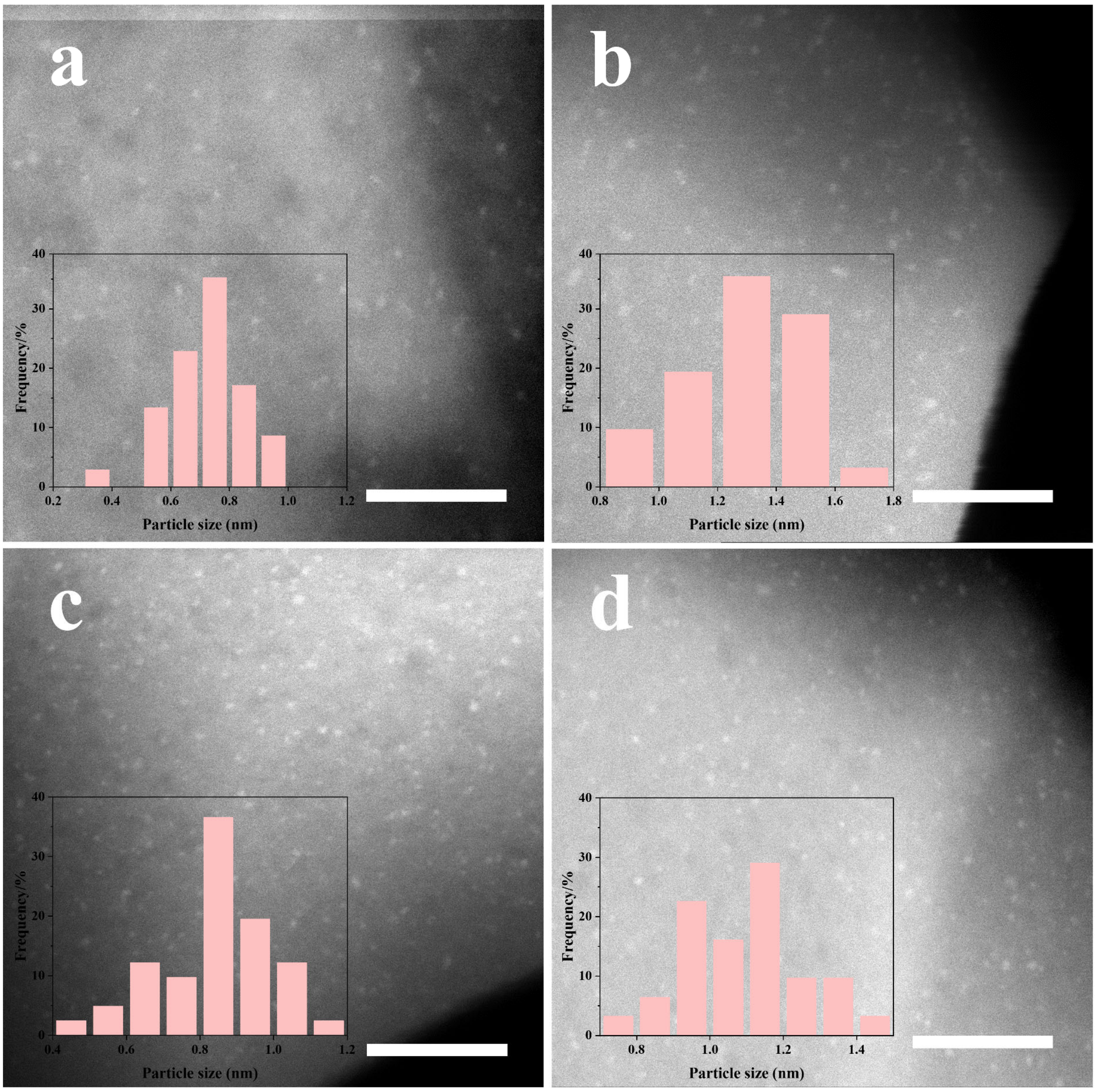

Figure S52. STEM of (a) Rh-MFI-cal, (b) Rh-MFI-calred, (c) Rh-(O)-P-MFI-cal and (d) Rh-(O)-P-MFI-calred samples. Scale bars correspond to 20 nm. Particle size distribution insert inside STEM Figures. The average particle sizes of each sample were calculated by the following equation: $d_{TEM} = \Sigma n_i d_i^3 / \Sigma n_i d_i^2$.

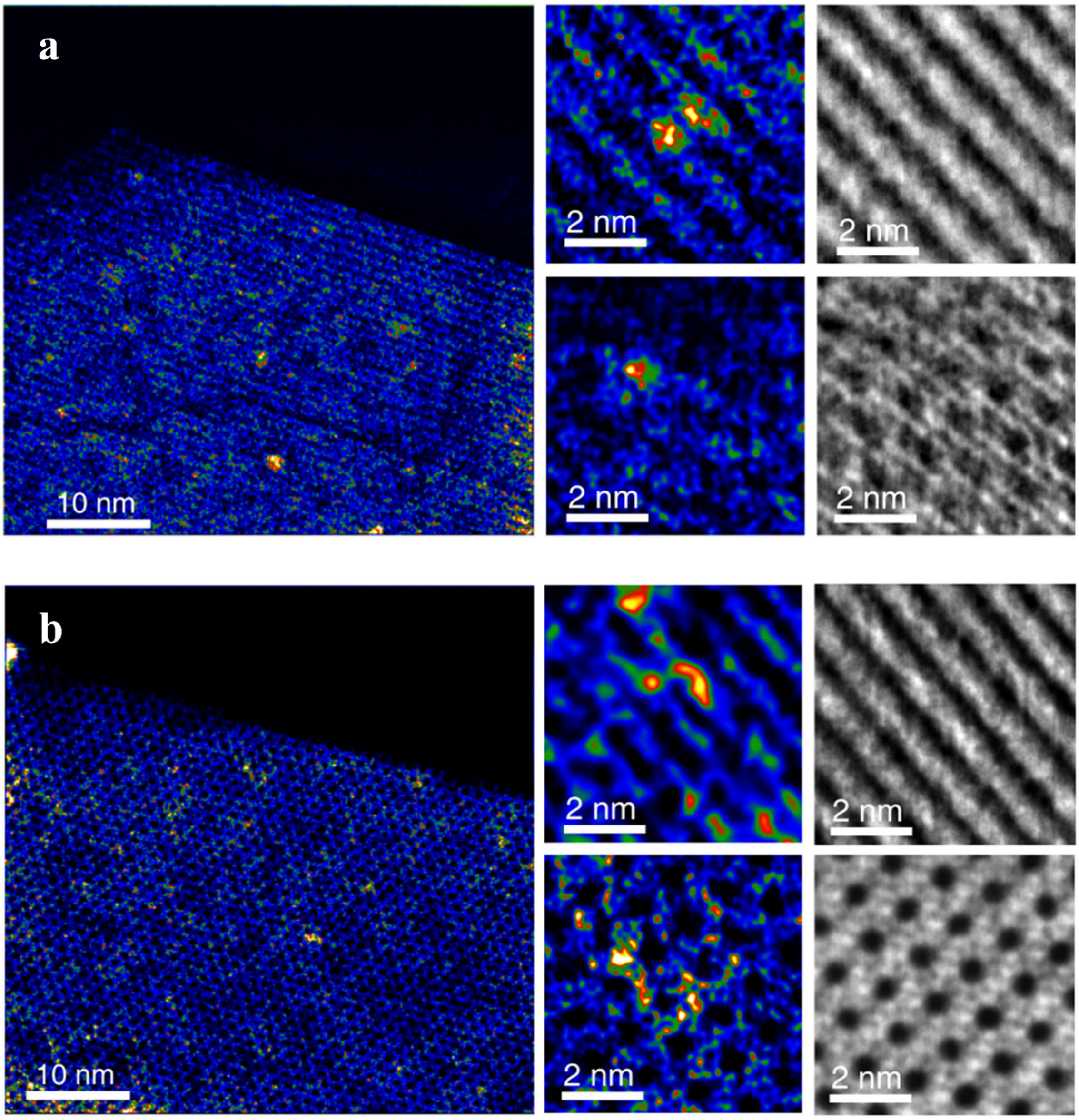

Figure S53. HAADF-STEM and iDPC images of (a) Rh-(O)-P-MFI-cal, (b) Rh-(O)-P-MFI-calred samples.

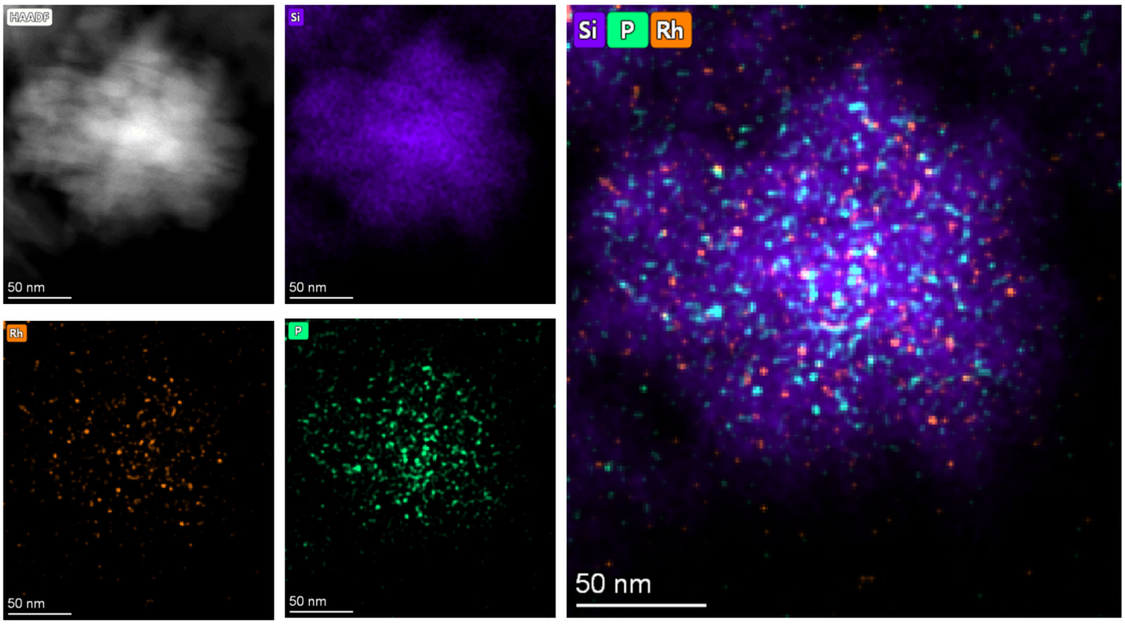

Figure S54. EDS elemental map of Rh-(O)-P-MFI-calred sample.

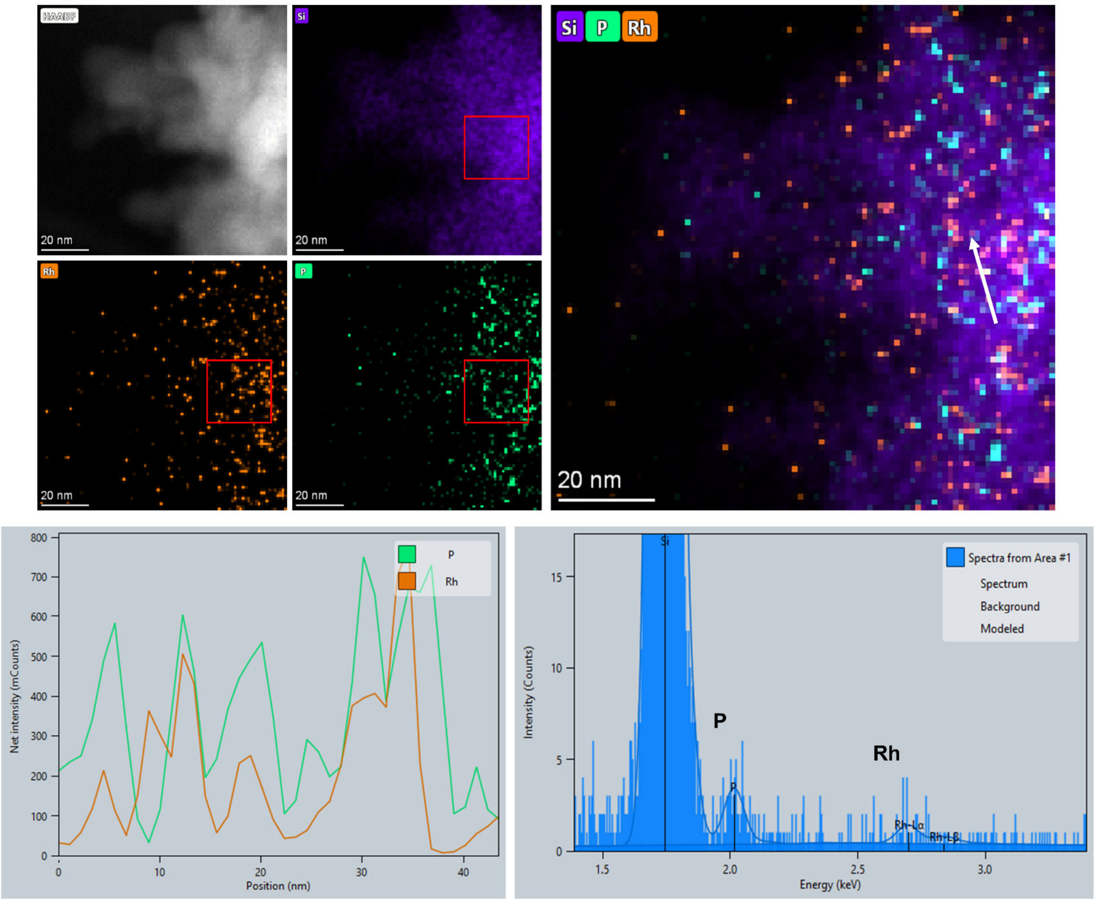

Figure S55. Line Profile extracted from the area marked with a red arrow, shows a good correlation between P and Rh signal.

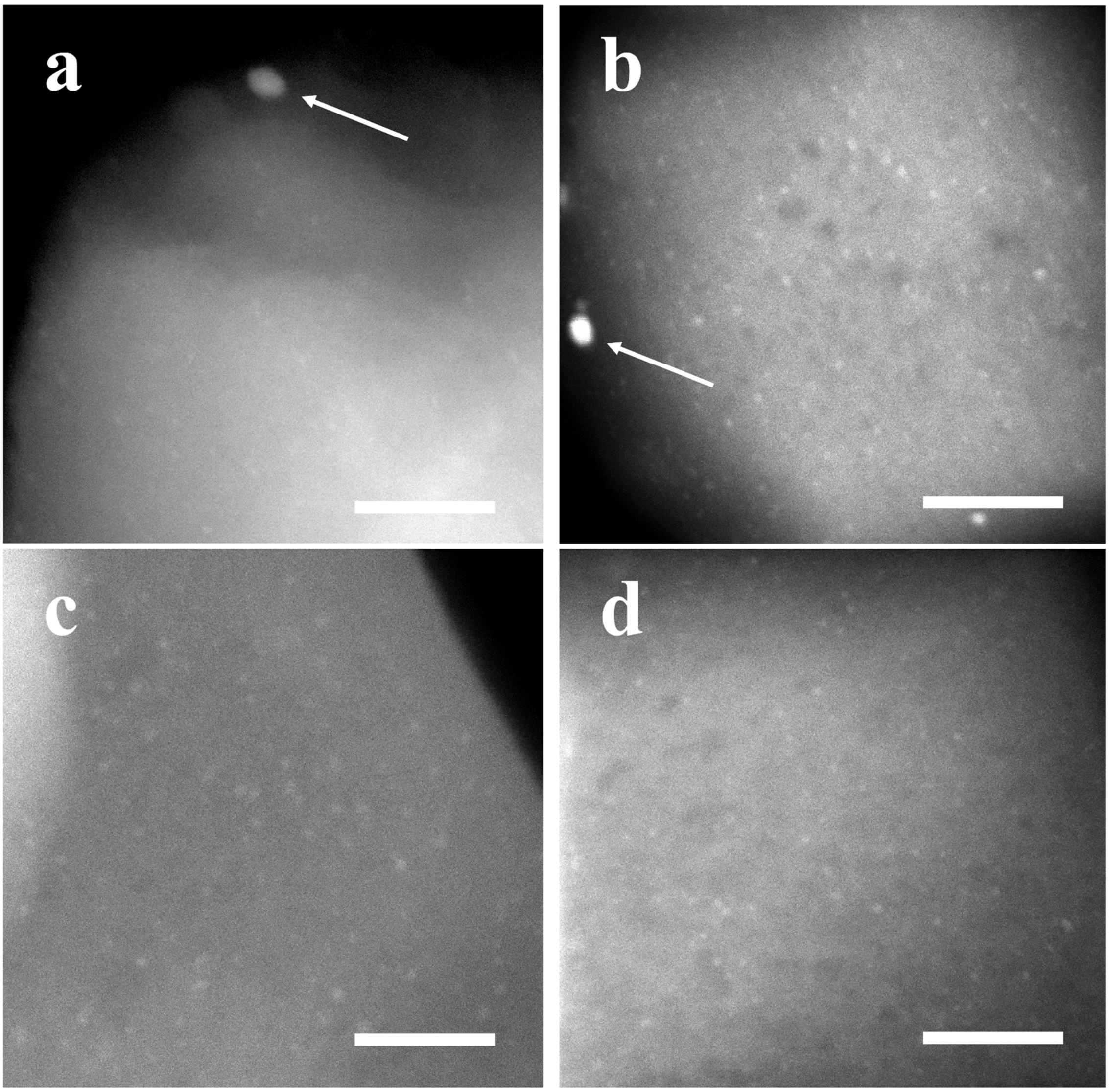

Figure S56. STEM of (a) Rh-MFI, (b) Rh-MFI-calred, (c) Rh-(O)-P-MFI-cal and (d) Rh-(O)-P-MFI-calred samples after hydroformylation reaction at 200 °C. Scale bars corresponds to 20 nm.

<u>^{31}PNMR</u>

^{31}P NMR spectra of Rh-(O)-P-MFI-calred sample (Figure S57), show signals at -5.8 and -16.2 ppm, which are typically assigned to phosphates with different coordinate environments (PO_4^{3-}, R_3P, etc.)[38, 39, 40]. Meanwhile, no phosphine was observed (+30 ppm to +100 ppm)[38, 39, 40]. In high chemical shift area (Figure S57, left), where the signal of phosphide (P^{3-}) usually appears, no signal was observed. This result indicates that in the calcined-reduced sample, P remained in

oxidized form, despite that the sample has been reduced at high temperature (600 °C). However, the low signal intensity due to low P amount limited further identification of P species by ^{31}P NMR.

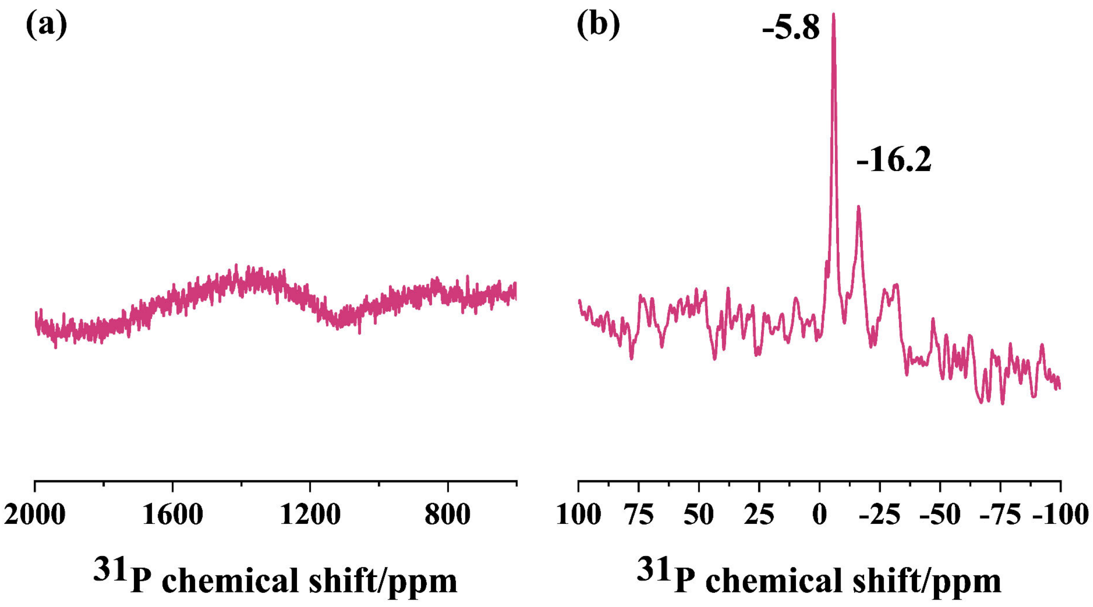

Figure S57. ^{31}P-NMR spectra of Rh-(O)-P-MFI-calred sample in (a) high chemical shift area and (b) low chemical shift area.

<u>X-ray adsorption at the P K-edge</u>

From the XAS results we observed edge position similar to Na_3PO_4 (Figure S58), even after the reduction, showing that P oxidation state is 5+.

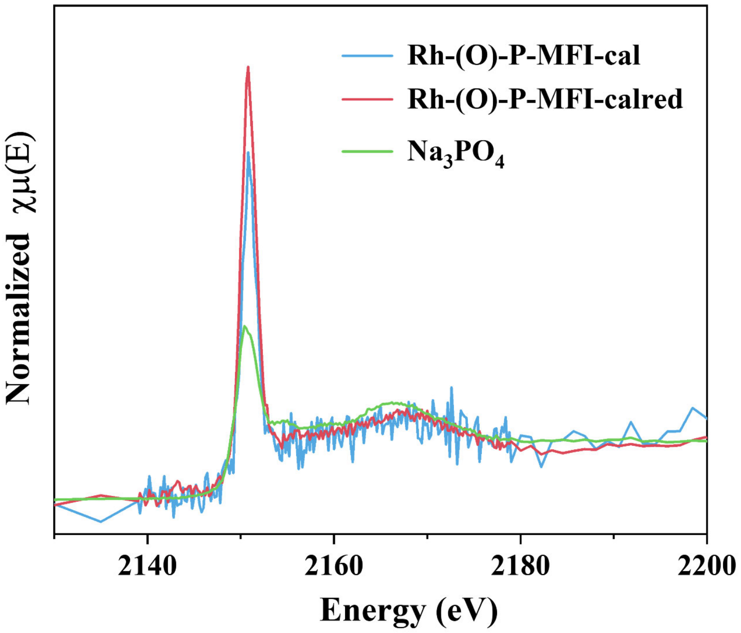

Figure S58. XANES spectra of Rh-P-MFI sample and the reference Na_3PO_4. Rh-(O)-P-MFI-cal was measured at room temperature, under He atmosphere. Rh-(O)-P-MFI-calred sample was measured at room temperature, under H_2 atmosphere after 600 °C reduction.

Due to the low P concentration in the sample, the signal-to-noise ratio didn't give EXAFS with good statistics to be analyzed and therefore the Rh: (O) P ratio in the stabilized catalyst is difficult to asses.

<u>IR-CO</u>

The IR-CO spectra at -65 °C are comparatively shown in Figure S59 for the un-doped (i.e. Rh-MFI-calred) (left) and the P-doped (i.e., Rh-(O)-P-MFI-calred) (right) samples. Rh^0 clusters (2064-2061 cm^{-1} for lineal CO and 1894-1892 and 1837-1823 cm^{-1} for bridge CO) are predominately observed together with some Rh^+ (2104-2107 and 2024-2021 cm^{-1}). No important changes are observed in the presence of P, thus we can say that the introduction of P did not cause observable differences among the samples

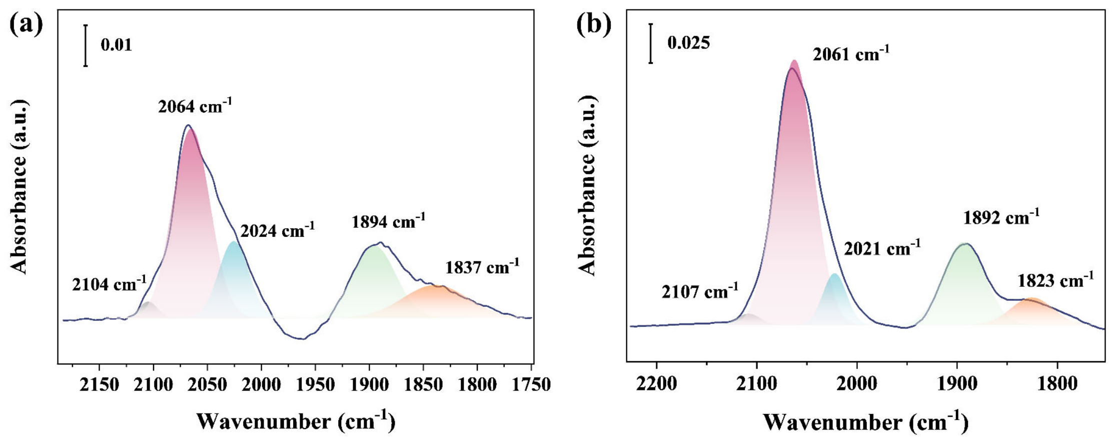

Figure S59. IR-CO spectra at -65°C of the Rh-MFI-calred (a), and Rh-(O)-P-MFI-calred (b) samples.

3.5.3 Catalytic study

The role of P on the catalytic behaviour of the samples is more evident in the Rh-MFI-calred sample, due to its dynamic behaviour under reaction conditions, where the initial Rh metal clusters suffers of oxidative disruption into single metal sites, which may be stabilized by adjacent P^{5+} ions as discussed later.

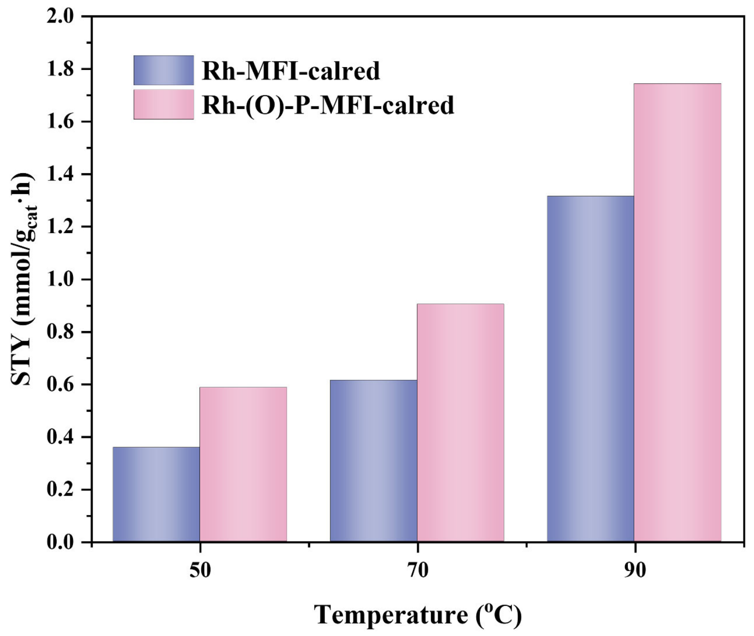

Figure S60. Role of P in the propanal formation on Rh-MFI-calred samples.

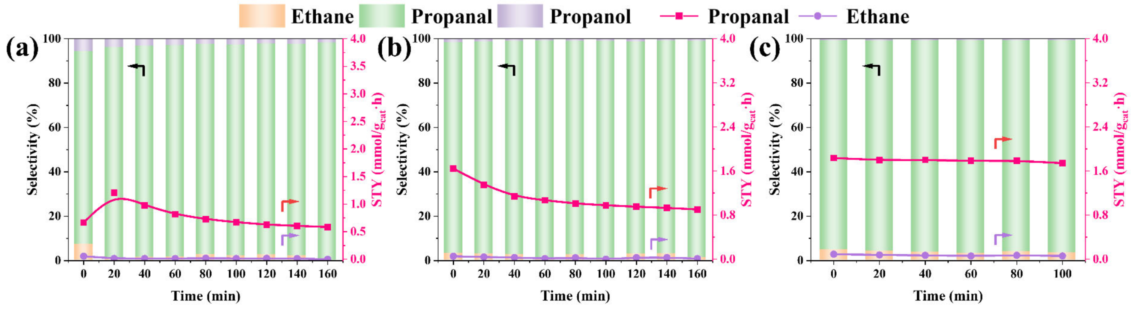

Figure S61. Catalytic performance of Rh-(O)-P-MFI-calred sample at (a) 50 °C, (b) 70 °C and (c) 90 °C and 10 bar. Right axe: space time yield (STY in mmol/g_{cat}.h) to propanal (red line) and ethane (violet line); Left axe: selectivity to products (%): propanal (marked as green column), ethane (brown) and propanol (violet). *Reaction conditions*: 100 mg catalyst diluted in 300 mg SiC. 35 mL/min total flow. GHSV 8000 h^{-1} (Ethylene: CO: H_2: N_2= 1: 1: 1: 0.5).

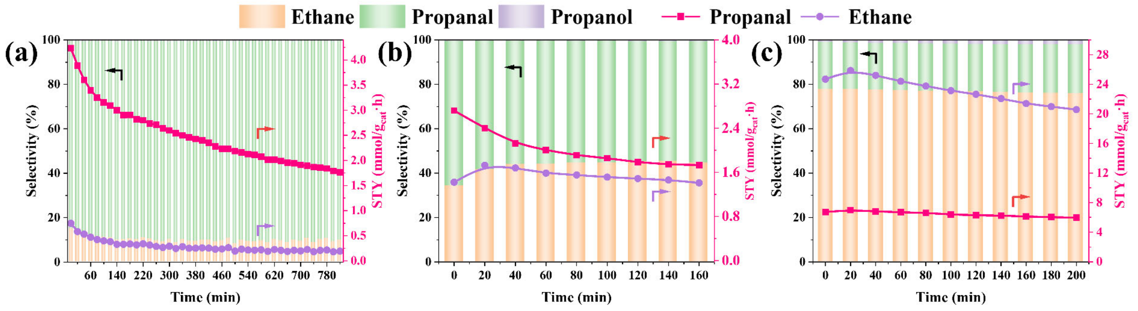

Figure S62. Catalytic performance of Rh-(O)-P-MFI-calred sample at (a) 120 °C, (b) 150 °C and (c) 200 °C and 10 bar. Right axe: space time yield (STY in mmol/g_{cat}.h) to propanal (red line) and ethane (violet line); Left axe: selectivity to products (%): propanal (marked as green column), ethane (brown) and propanol (violet). *Reaction conditions*: 100 mg catalyst diluted in 300 mg SiC. 35 mL/min total flow. GHSV 8000 h^{-1} (Ethylene: CO: H_2: N_2= 1: 1: 1: 0.5).

Table S9. Ethylene conversion and selectivity to propanal on the Rh-(O)-P-MFI-calred sample.

WHSV (mL/g·h)	GHSV (h^{-1})	T (°C)	Conversion(%)	Selectivity to propanal (%)	STY-Propanal (mmol/g_{cat}·h)	TOF（h^{-1}）
21000 [a]	8000	50	0.52	95.10	1.21	45.94
			0.24	98.32	0.59	22.33
21000 [a]	8000	70	0.71	95.35	1.64	62.63

			0.38	97.69	0.90	34.42
21000 [a]	8000	90	0.80	94.51	1.84	70.00
			0.75	95.89	1.74	66.43
21000 [a]	8000	120 [b]	2.06	84.54	4.23	161.32
			0.81	89.69	1.76	67.14
21000 [a]	8000	150	1.71	65.36	2.72	103.62
			1.30	54.75	1.73	65.98
21000 [a]	8000	200	13.66	21.00	6.99	266.34
			11.12	22.01	5.96	227.28

[a] Without colour are initial values, while those with a green background correspond to final values

[b] Reaction duration was 13h at 120 ºC, while at the others temperatures, 2-6h

Table S10. Apparent activation energies according to the Arrhenius plot based on reaction rates of ethylene hydroformylation to propanal and ethylene hydrogenation to ethane, in the temperature range 50-90°C for propanal and 50-120 ºC and 120-200 °C for ethane [a].

Sample	E_a Propanal (KJ/mol)	E_a Ethane (KJ/mol)	
		50-120 ºC	120-200 ºC
Rh-MFI-cal	19.5 ± 1.5	18.5 ± 0.8	131.4 ± 0.2
Rh-MFI-calred	31.6 ± 2.1	32.2 ± 0.1	95.5 ± 0.5
Rh-(O)-P-MFI-calred	26.5 ± 2.2	45.1 ± 1.5	89.2 ± 0.6
Rh/MFI-calred	61.3 ± 3.2	22.4 ± 0.5	87.8 ± 1.0

[a] The calculation of Ea are based on final STY values of different temperature.

3.5.4 Operando studies

X-ray adsorption spectroscopy

EXAFS analysis of Rh-(O)-P-MFI-calred under reduction and reacted at 50, 90 and 200 °C at 10 bar.

Table S11. Multi (k^1, k^2, k^3)-weighted fits carried out in r-space (1-3.2 Å) over a k-range of 3-12 $Å^{-1}$ using a Hannings window (dk 1), and $S_0^2 = 0.9$. Bond distances and disorder parameters (Δr_{eff} and σ^2) were allowed to float having initial values of 0.0 Å and 0.003 $Å^2$ respectively, with a universal E_0 and $\Delta E_0 = 0$ eV.

Rh-(O)-P-MFI-calred H_2 220min

	R_{FACTOR}	X^2_v	Var. No.	k-range ($Å^{-1}$)	r-range (Å)	ΔE_0 (eV)
	0.015	0.7	5	3-12	1-3.2	-5.7(0.7)
**PATHS**						
**Rh-C/N/O**	**N**		**r (Å)**		**σ^2 (x10^3 $Å^2$)**	
	0.8		1.99(0.04)		13.5(5.6)	
**Rh-Rh**	**N**		**r (Å)**		**σ^2 (x10^3 $Å^2$)**	
	5.5		2.66(0.01)		9.2(0.4)	

Rh-(O)-P-MFI-calred reaction feed 225min

	R_{FACTOR}	X^2_v	Var. No.	k-range ($Å^{-1}$)	r-range (Å)	ΔE_0 (eV)
	0.008	0.6	5	3-12	1-3.2	-4.8(0.5)
**PATHS**						
**Rh-C/N/O**	**N**		**r (Å)**		**σ^2 (x10^3 $Å^2$)**	
	0.8		1.98(0.03)		15.0(0.5)	
**Rh-Rh**	**N**		**r (Å)**		**σ^2 (x10^3 $Å^2$)**	
	5.5		2.68(0.00)		6.9(0.2)	

Rh-(O)-P-MFI-calred reaction feed 376min

	R_{FACTOR}	X^2_v	Var. No.	k-range ($Å^{-1}$)	r-range (Å)	ΔE_0 (eV)
	0.008	0.8	7	3-12	1-3.2	-2.3(1.0)
**PATHS**						

Rh-C/N/O	**N**	**r (Å)**	**σ^2 (x10^3 Å^2)**
	1.0	1.85(0.05)	13.0(2.0)
	**N**	**r (Å)**	**σ^2 (x10^3 Å^2)**
	1.8	2.00(0.01)	6.3(1.7)
**Rh-Rh**	**N**	**r (Å)**	**σ^2 (x10^3 Å^2)**
	2.8	2.71(0.01)	5.8(0.3)
**Rh-O**	**N**	**r (Å)**	**σ^2 (x10^3 Å^2)**
	1.0	3.00(0.05)	13.0(2.0)
**Rh-O-C**	**N**	**r (Å)**	**σ^2 (x10^3 Å^2)**
	0.8	3.00(0.05)	13.0(2.0)
**Rh-C-O-C**	**N**	**r (Å)**	**σ^2 (x10^3 Å^2)**
	0.8	3.00(0.05)	13.0(2.0)

Rh-(O)-P-MFI-calred reaction feed 490min

	R_{FACTOR}	X^2_ν	Var. No.	k-range (Å^{-1})	r-range (Å)	ΔE_0 (eV)
	0.012	1.5	7	3-13	1-3.2	-4.7(1.0)

PATHS

**Rh-C/N/O**	**N**	**r (Å)**	**σ^2 (x10^3 Å^2)**
	2.0	1.84(0.04)	11.9(5.0)
	**N**	**r (Å)**	**σ^2 (x10^3 Å^2)**
	1.5	2.03(0.03)	10.0(2.1)
**Rh-Rh**	**N**	**r (Å)**	**σ^2 (x10^3 Å^2)**
	4.5	2.69(0.00)	4.2(0.3)
**Rh-O**	**N**	**r (Å)**	**σ^2 (x10^3 Å^2)**
	1.0	2.99(0.04)	11.9(5.0)
**Rh-O-C**	**N**	**r (Å)**	**σ^2 (x10^3 Å^2)**
	2.0	2.99(0.04)	11.9(5.0)
**Rh-C-O-C**	**N**	**r (Å)**	**σ^2 (x10^3 Å^2)**
	1.0	2.99(0.04)	11.9(5.0)

Rh-(O)-P-MFI-calred H_2 (220 min)

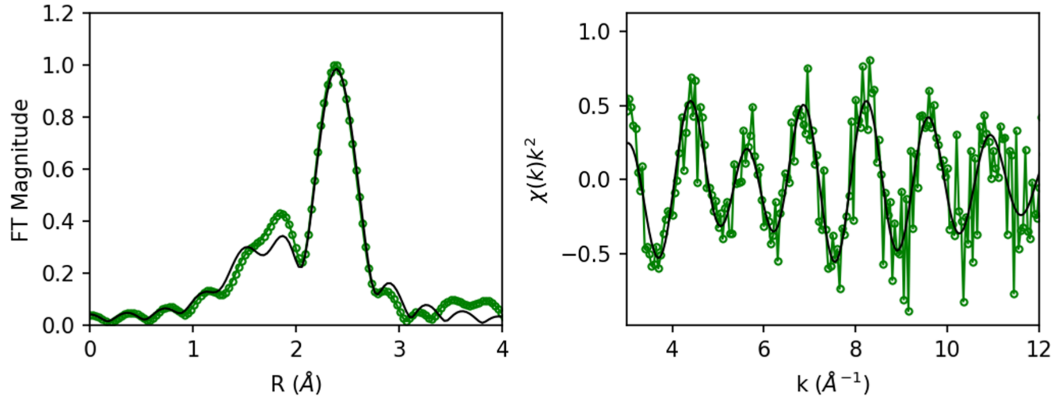

Rh-(O)-P-MFI-calred reaction feed (225 min)

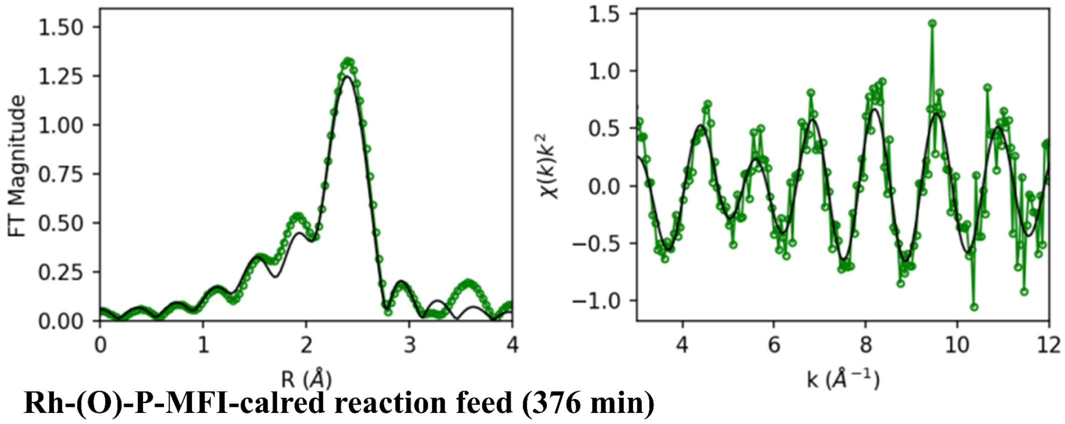

Rh-(O)-P-MFI-calred reaction feed (376 min)

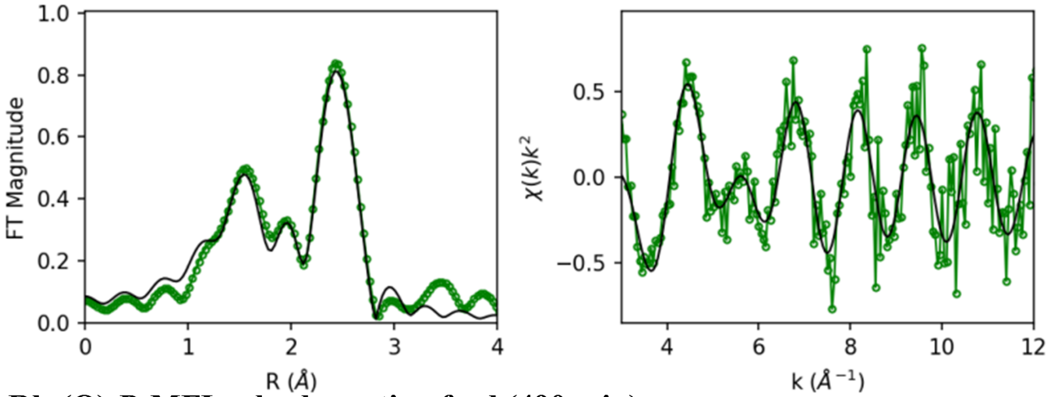

Rh-(O)-P-MFI-calred reaction feed (490 min)

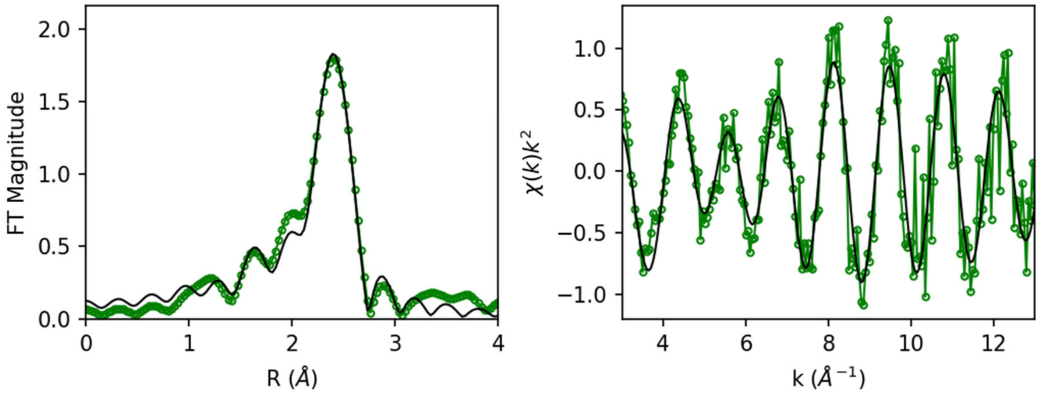

Figure S63. EXAFS fit of Rh-(O)-P-MFI-calred under reduction and reaction conditions at 50, 90 and 200 ºC at 10 bar presented in Table S11.

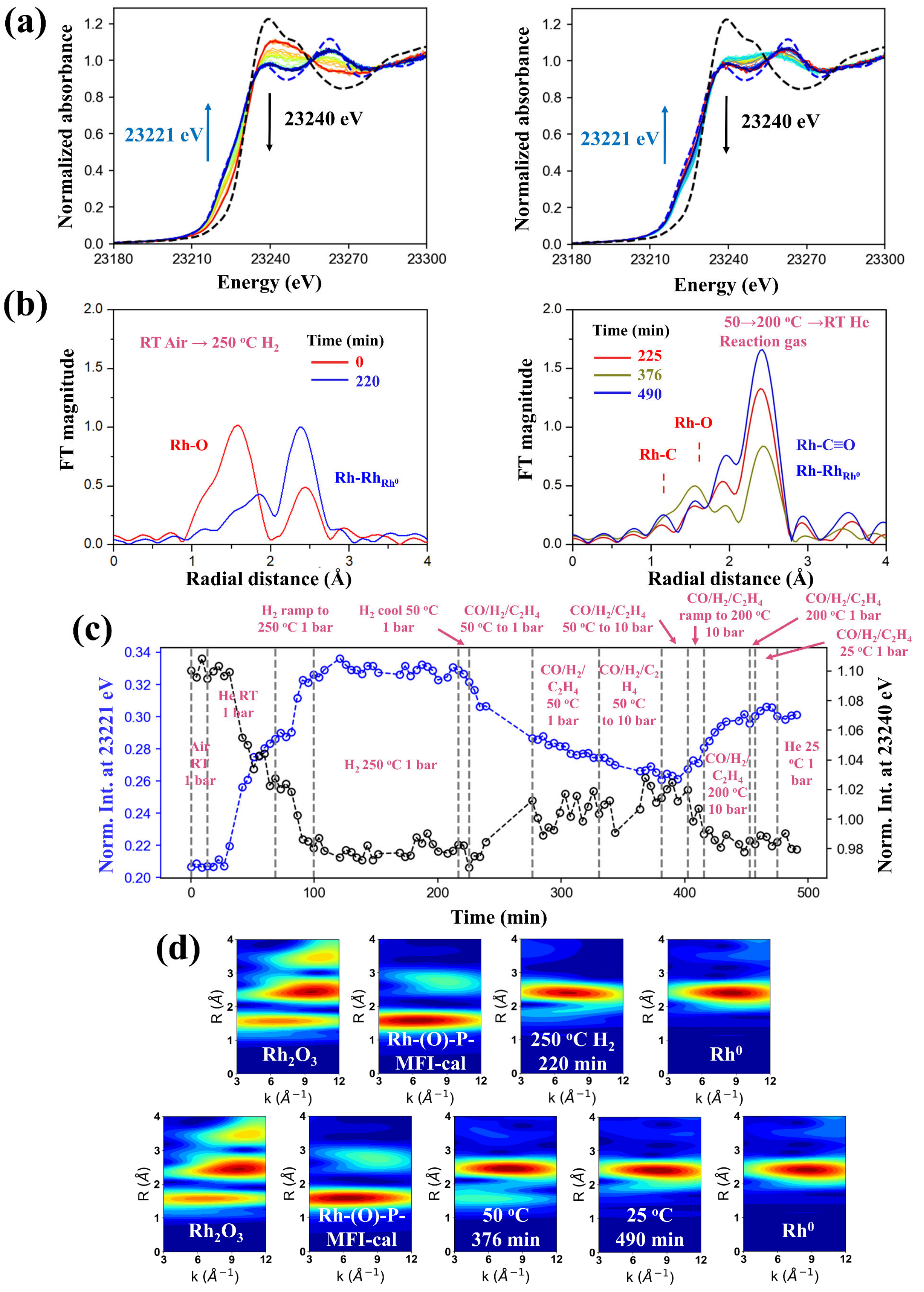

Figure S64. Effect of temperature on the XAS profile under reaction conditions: (a) Rh-(O)-P-MFI XANES spectra at the Rh K-edge (left) under H_2 flow at 250 °C, 1bar and 220 min; (right) under reaction feed at 50 °C, 10 bar over 151 min (red to green line) and further increasing

temperature to 200 °C over a final period of 114 min (green to blue line). Spectra, overlaid with Rh(0) (blue dashes) and to Rh_2O_3 (black dashes); (b) Fourier transformed EXAFS signal (k^2-weigthed, Hanning window, k-range 3-12.0 $Å^{-1}$) showing the first and last spectrum under H_2 (left) and at key points in the reaction feed respectively (right); (c) Evolution of the normalized spectral intensities at 23221 eV corresponding to Rh-CO/Rh(0) (blue) and 23240 eV corresponding to Rh_2O_3 (black). (d) Cauchy wavelet transforms of EXAFS signals shown in (b)

Similarly to the Rh-MFI, the Rh-(O)-P-MFI sample shows an initial Rh_2O_3 to Rh^0 conversion by hydrogenation and heating at 250 °C. In reaction conditions ($C_2^{=}/CO/H_2/N_2$(1/1/1/0.5 molar)) at 50 °C part of the Rh^0 is converted in Rh-CO phase, and differently by the Rh-MFI case, also in isolated Rh^{3+} sites. By increasing the temperature to 200 °C the Rh^0 phase seems to be partially restored while the Rh^{3+} site disappear.

IR- MS

The mas spectra profile of un-doped (left) and P doped (right) calcined reduced Rh-MFI samples in ethylene hydroformylation is plotted in Figure S65. Clearly propanal formation is promoted in the presence of P.

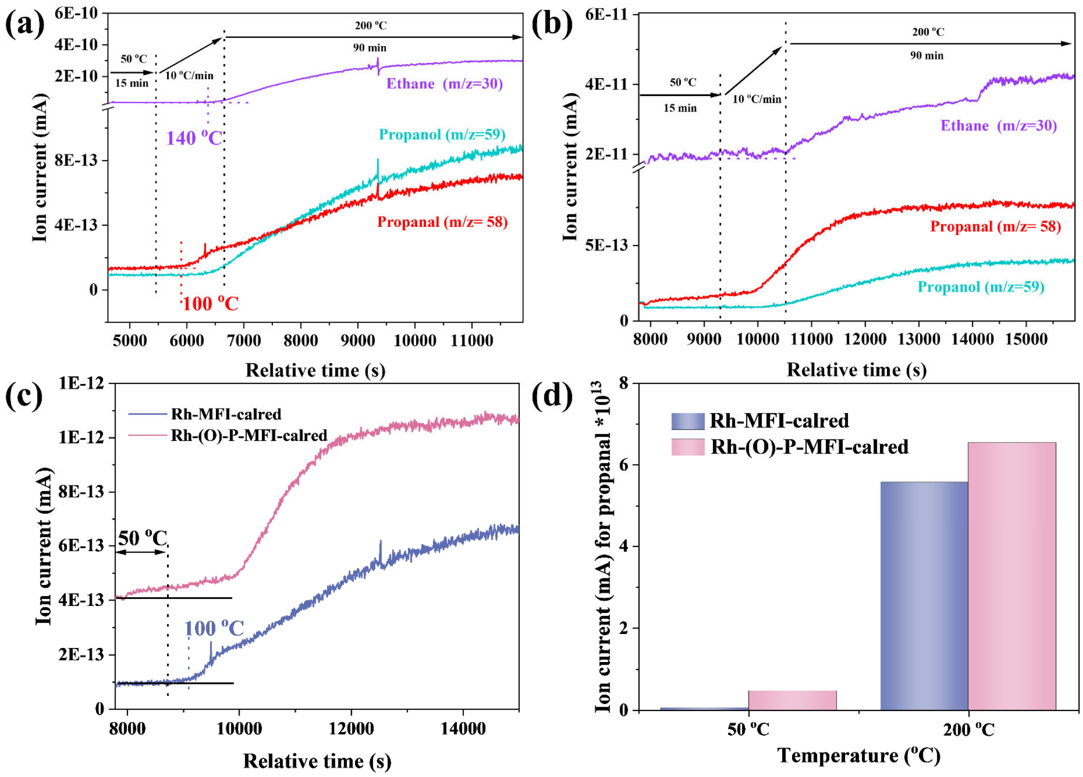

Figure S65. IR-MS profile of un-doped (a) and P doped (b) calcined reduced Rh-MFI samples. (c) Expanded plot of propanal formation on un-doped (in violet) and P doped (in pink) samples. (d) MS value of propanal formation on un-doped (in violet) and P doped (in pink) samples at 50 and 200 °C.

4. Kinetic studies in ethylene hydroformylation

Rh-MFI-cal and Rh-MFI-calred catalysts.

The effect of CO and H_2 partial pressures are studied on both samples operating under stable conditions (at 50 °C and 70 °C respectively) and 10 bar by varying the H_2/CO ratio, while the partial pressure of C_2H_4 remained constant. The measured rates were fitted by a power law kinetic model following the expression: $r_{Propanal}=k\cdot P_{H2}^{\alpha}\cdot P_{CO}^{\beta}\cdot P_{C2H4}^{\gamma}$, where P_{C2H4}^{γ} is constant, therefore the expression is rewritten as $r_{Propanal}=k'\cdot P_{H2}^{\alpha}\cdot P_{CO}^{\beta}$, where $k'= k\cdot P_{C2H4}^{\gamma}$, α and β correspond to the apparent reaction orders with respect to H_2 and CO partial pressures, respectively. The conversion of ethylene was kept under 1% so that the reaction operated under differential conditions, that is, in the kinetic regime. Thus, it is likely that the apparent orders here are representative of the direct rate of the kinetically relevant step of the reaction mechanism.

Figure S66 shows the effect of the partial pressure of CO and H_2 on the rates of propanal formation on the Rh-MFI-cal (violet line) and Rh-MFI-calred (red line) samples.

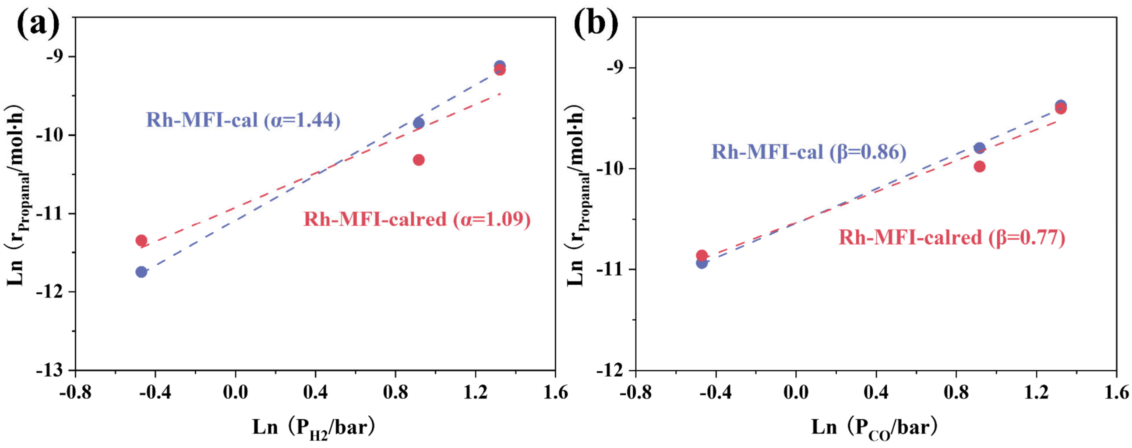

Figure S66. Reaction orders for Rh-MFI-cal (violet) and Rh-MFI-calred (red) catalysts at 50 °C and 70 °C, respectively. Rates for hydroformylation to propanal formation as a function of (a) H_2 partial pressure (P_{CO} = 2.5 bar, P_{C2H4} = 2.5 bar) and (b) CO partial pressure (P_{H2} = 2.5 bar, P_{C2H4} = 2.5 bar) for a total operating pressure of 10 bar.

In addition, the influence of the CO and H_2 partial pressure, and different ratios of $H_2/CO/C_2H_4$ on the formation rate to propanal is displayed in Table S12.

Table S12. Effect of the partial pressure of H_2 and CO, and the ratio $H_2/CO/C_2H_4$ on the formation rate of propanal (r, $mol_{Propanal}/g_{cat}\cdot h$) in the hydroformylation of ethylene on Rh-MFI cal and Rh-MFI calred catalysts. Reaction conditions: 50 °C (Rh-MFI-cal) and 70 °C (Rh-MFI-calred), 10 bar and gas mixture flow 80 mL/min.

Catalyst	$H_2/CO/C_2H_4$	Partial Presure (bar)					$r_{propanal}$ ($mol_{Propanal}/g_{cat}\cdot h$)
		P_{CO}	P_{H2}	P_{C2H4}	P_{Ar}	P_{N2}	
Rh-MFI-cal	1/4/4	0.625	2.500	2.500	3.125	1.250	$1.8x10^{-4}$
	1/1/1	2.500	2.500	2.500	1.250	1.250	$5.5x10^{-4}$
	1.5/1/1	3.750	2.500	2.500	0.000	1.250	$8.4x10^{-4}$
	4/1/4	2.500	0.625	2.500	3.125	1.250	$7.8x10^{-5}$
	1/1/1	2.500	2.500	2.500	1.250	1.250	$5.2x10^{-4}$
	1/1.5/1	2.500	3.750	2.500	0.000	1.250	$1.1x10^{-3}$
Rh-MFI-calred	1/4/4	0.625	2.500	2.500	3.125	1.250	$1.9x10^{-4}$
	1/1/1	2.500	2.500	2.500	1.250	1.250	$4.6x10^{-4}$
	1.5/1/1	3.750	2.500	2.500	0.000	1.250	$8.1x10^{-4}$
	4/1/4	2.500	0.625	2.500	3.125	1.250	$1.2x10^{-4}$
	1/1/1	2.500	2.500	2.500	1.250	1.250	$3.3x10^{-4}$
	1/1.5/1	2.500	3.750	2.500	0.000	1.250	$1.1x10^{-3}$

Figure S66 suggests that there is a positive effect of H_2 and CO partial pressure on propanal on both catalysts. In addition, it can be confirmed by means of the $H_2/CO/C_2H_4$ ratio that when there is a low ratio of either H_2 or CO, the rate to propanal is also low (Table S12). According to these results we can suggest that the reaction mechanism is probably the same, but it may be that the rate determining step (rds) changes (i.e., the number of species involved changes) for each active site (Rh^{3+} and Rh^{+}), and/or that these sites modify the adsorption energies of the intermediates involved in the propanal formation pathway, which causes that the apparent activation energy of the reaction changed between catalysts. In particular, the apparent orders of positive CO and with very similar values between both catalysts suggest that CO participates in the rds of the reaction mechanism on both active sites, with moderate CO adsorption (i.e., similar CO adsorption enthalpies) preventing deactivation by site poisoning. Meanwhile, the positive contribution of H_2 and the apparent order variation from 1.44 (Rh-MFI-cal) to 1.09 (Rh-MFI-calred) suggest that the RDS in both catalysts is not the same; in Rh-MFI-calred participates at least two H* while in Rh-MFI-cal involved at least three H* adsorbed. Furthermore, a different rds supports the observed differences in the apparent activation energies determined for each active site (Table S10).

Rh/SiO_2 impregnated sample

For comparative propose, the effect of the partial pressure of CO and H_2 has been also studied in calcined and calcined reduced Rh impregnated samples over amorphous SiO_2 (Rh/SiO_2) with 0.1wt% Rh loading

Rh/SiO_2 sample is prepared by the same method to impregnated Rh/MFI, except the support is amorphous Aerosil 200.

Operando IR-MS studies (Figure S67) shows the formation of Rh^{+} species in the Rh/SiO_2-cal sample and small Rh^{0} nanoparticles in the Rh/SiO_2-calred one. Notice that Rh^{3+} is not detected in any catalyst.

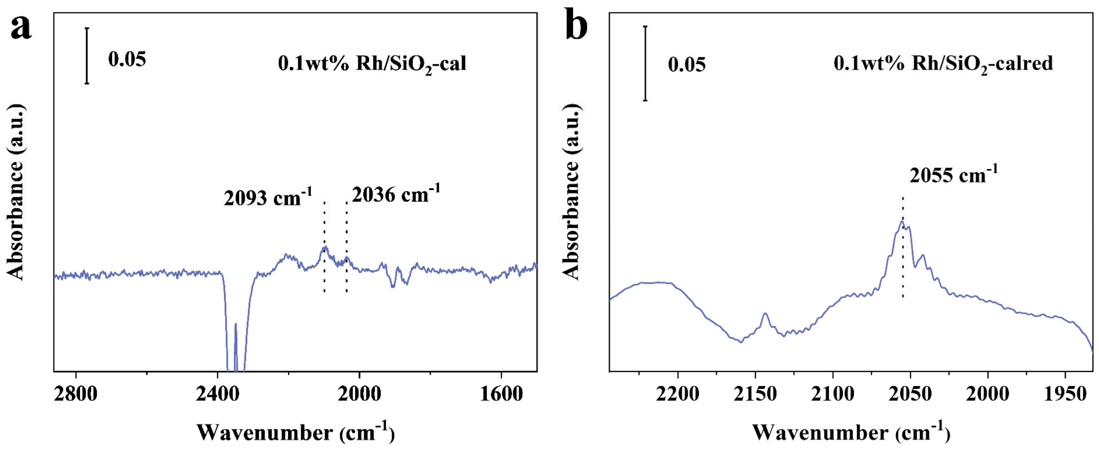

Figure S67. IR-CO of 0.1wt% Rh/SiO$_2$. (a) Rh/SiO$_2$-cal and (b) Rh/SiO$_2$-calred samples under operando ethylene hydroformylation reaction at 50 ºC in the IR catalytic cell.

Figure S66 shows the effect of the partial pressure of CO and H_2 on the rates of propanal formation on the Rh/SiO$_2$-cal (violet line) and Rh/SiO$_2$-calred (red line) samples.

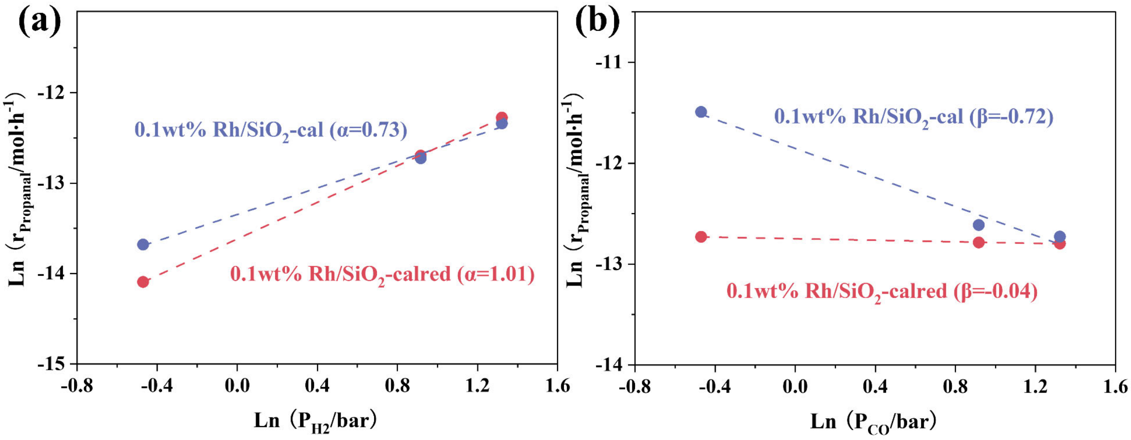

Figure S68. Reaction orders for 0.1wt% Rh/SiO$_2$-cal (violet) and 0.1wt% Rh/SiO$_2$-calred (red) catalysts at 70 °C. Rates for hydroformylation to propanal formation as a function of (a) H_2 partial pressure (P_{CO} = 2.5 bar, P_{C2H4} = 2.5 bar) and (b) CO partial pressure (P_{H2} = 2.5 bar, P_{C2H4} = 2.5 bar) for a total operating pressure of 10 bar.

In addition, the influence of the CO and H_2 partial pressure, and different ratios of H_2/CO/C_2H_4 on the formation rate and selectivity to propanal is displayed in Table S13.

Table S13. Effect of the partial pressure of H_2 and CO, and the ratio $CO/H_2/C_2H_4$ on the formation rate of propanal (r, $mol_{Propanal}/g_{cat}\cdot h$) in the hydroformylation of ethylene on 0.1wt% Rh/SiO_2-cal and 0.1wt% Rh/SiO_2-calred catalysts. Reaction conditions: 70 °C, 10 bar and gas mixture flow 80 mL/min.

Catalyst	$H_2/CO/C_2H_4$	Partial Presure (bar)					$r_{propanal}$
		P_{CO}	P_{H2}	P_{C2H4}	P_{Ar}	P_{N2}	$(mol_{Propanal}/g_{cat}\cdot h)$
**0.1wt% Rh/SiO_2-cal**	1/4/4	0.625	2.500	2.500	3.125	1.250	$1.0x10^{-4}$
	1/1/1	2.500	2.500	2.500	1.250	1.250	$3.3x10^{-5}$
	1.5/1/1	3.750	2.500	2.500	0.000	1.250	$3.0x10^{-5}$
	4/1/4	2.500	0.625	2.500	3.125	1.250	$1.1x10^{-5}$
	1/1/1	2.500	2.500	2.500	1.250	1.250	$2.9x10^{-5}$
	1/1.5/1	2.500	3.750	2.500	0.000	1.250	$4.3x10^{-5}$
**0.1wt% Rh/SiO_2-calred**	1/4/4	0.625	2.500	2.500	3.125	1.250	$2.9x10^{-5}$
	1/1/1	2.500	2.500	2.500	1.250	1.250	$2.7x10^{-5}$
	1.5/1/1	3.750	2.500	2.500	0.000	1.250	$2.7x10^{-5}$
	4/1/4	2.500	0.625	2.500	3.125	1.250	$7.5x10^{-6}$
	1/1/1	2.500	2.500	2.500	1.250	1.250	$3.0x10^{-5}$
	1/1.5/1	2.500	3.750	2.500	0.000	1.250	$4.6x10^{-5}$

The ca. 0 order on the 0.1wt% Rh/SiO_2-calred catalyst and the negative order for the 0.1 wt% Rh/SiO_2-cal catalyst suggest that active sites formed during reaction from calcined catalysts, i.e., Rh^+ easily adsorbs CO in a stable way ($Rh(I)(CO)_2$), which can obstruct ethylene adsorption at the same site, thus the site is poisoned by carbonyls. Therefore, we have observed in the case of the Rh/SiO_2 calcined catalyst that higher CO coverage has a negative effect on ethylene hydroformylation, as previously reported in the literature[11, 23, 24]. However, when the calcined catalyst is reduced (0.1wt% Rh/SiO_2-calred), these sites change on the surface, forming mostly Rh^0 nanoparticles that do not strongly interact with CO, showing almost zero order to CO. Meanwhile, the H_2 in both cases has a positive contribution. The apparent orders suggest that at least 1 or 2 adsorbed H* participate in the rds, as in the Rh-MFI-calred sample.

When we compare the results obtained on Rh-MFI and Rh/SiO_2, we can say that the local environment of Rh sites and their chemical properties has a strong influence on the catalytic and kinetic behaviour. Thus, the Lewis acid properties of metal cations can be tuned due to confinement effects, altering their coordination degree and electrophilicity, which in the case of zeolites is different compared to the same cation in other supports. This affect their interaction with reactants and intermediate species and in definitive have an effect on their catalytic performance.

5. Propylene hydroformylation with syngas

Table S14 shows the catalytic performance of Rh-MFI and Rh-(O)-P-MFI catalysts in the hydroformylation of propylene, where (l/b) represent the linear to branched molar ratio of aldehydes. Comparison with state-of-the-art phosphine free catalysts is also included.

Table S14. Catalytic performance of Rh-MFI-cal and Rh-(O)-P-MFI-calred catalysts in the gas phase hydroformylation of propylene. (l/b) represents the linear to branched molar ratio of aldehydes. State-of-the-art heterogeneous phosphine free catalyst are also included [a].

Catalyst	WHSV (mL/g·h)	GHSV (h^{-1})	T (ºC)	P (bar)	Conversion (%)	Selectivity to aldehydes (%)	TOF (h^{-1})	l/b	Reference
Rh-MFI-cal	28000	10500	50	1	0.06	3.9	0.38	2.23	This work
			90	1	0.07	49.4	5.26	3.56	This work
			120	1	0.18	67.6	18.31	4.17	This work
Rh-(O)-P-MFI-calred	28000	10500	50	1	0.06	11.3	0.76	2.08	This work
			90	1	0.10	43.0	4.65	2.37	This work
			120	1	0.21	54.3	12.18	2.42	This work
Rh/SiO_2	2800	-	140	1	0.20	42	0.10	2.33	41
Rh-Li/SiO_2	2800	-	140	1	0.85	59	0.64	2.70	41
Rh-K/SiO_2	2800	-	140	1	0.55	48	0.34	3.35	41
Rh-Rb/SiO_2	2800	-	140	1	0.34	46	0.21	4.00	41
Rh-Mo/SiO_2	3600	3600	145	1	0.65	39 [b]	88.1 [b]	-	20

Rh/SiO_2	3600	3600	160	1	0.05	40	0.66	6.50	42
Rh-V-SiO_2	3600	3600	160	1	0.39	3	0.24	1.5	42
$RhCo_3$/MCM-41	20000	-	160	1	< 2	47 [c]	~1.20	3.13	24

[a] All hydroformylation reactions were conducted under gas-solid conditions and H_2/CO=1:1. Batch reactor reaction and phosphine-based catalysts were not summarized in this table.

[b] Only n-butanol and iso-butanol are the products. Selectivity of aldehydes below 1%.

[c] Aldehydes and alcohols are included.

As shown in Table S14, at 120 ºC, the linear to branched molar ratio to aldehydes (l/b) is 4.17 in the Rh-MFI-cal sample with a total selectivity to aldehydes of 67.6%, whereas in the Rh-(O)-P-MFI sample the l/b is 2.42 and the selectivity to aldehydes of 54.3%. Both values are some higher compared to state-of-the-art phosphine free Rh based catalysts operating under atmospheric gas phase conditions (see Table S14). It is proposed that steric effects imposed by the zeolite, may restrict the adsorption configuration of propylene enhancing the regioselectivity to linear aldehyde, while the role of electronic effects cannot be discarded.

6. Physico-chemical properties of the catalysts

Table S15. Chemical composition physicochemical properties of different Rh-MFI-cal, Rh-MFI-calred and Rh-P-MFI-calred samples

Sample	Rh content (wt%) [a]	P content (wt%) [a]	C content (wt%) [b]	N content (wt%) [b]	S_{BET} (m^2/g) [c]	V_{micro} (cm^3/g) [c]
Rh-MFI-cal	0.23	-	0.03	0.16	350	0.17
Rh-MFI-calred	0.23	-	0.02	0.13	374	0.17
Rh/MFI-calred	0.31	-	0.03	0.15	369	0.16
Rh-(O)-P-MFI-calred	0.27	0.034	0.05	0.17	382	0.17

[a] Measured by ICP-AES

[b] Measured by elemental analysis

[c] Measured by N_2 adsorption/desorption

7. Appendix

Appendix 1 Selection of the adequate Experimental conditions for IR-CO experiments

IR-CO has been shown as a powerful tool for characterization of Rh species. Adsorption of CO on Rh ions (Rh^{n+}, n= 1-3), Rh clusters ($Rh_x(CO)_y$) and metal nanoparticles have been widely discussed and reported in the literature[43, 44, 45, 46, 47]. However, when using CO as probe molecule in IR studies, it is important to define the experimental conditions in which CO behaves inactive and the sample remains stable. That is even more important in cases where reconstruction of metal sites under CO atmosphere may take place, as in the case of Rh based catalysts. In fact the redispersion of Rh NP into smaller clusters or single sites have been widely demonstrated in the literature[31]. In this section the experimental IR conditions used in our work are described, where changes induced by the adsorption of CO used as probe molecule can be neglected.

In Figure S69, IR-CO experiments done at 25 °C versus -65 °C are compared. A re-dispersion of the Rh metal nanoparticle into single Rh (I) sites is observed at 25 °C in the presence of CO. This phenomena is absent when operating at -65 °C. This is very important when literature data are discussed specifically because most of the literature studies are done at ~30 °C.

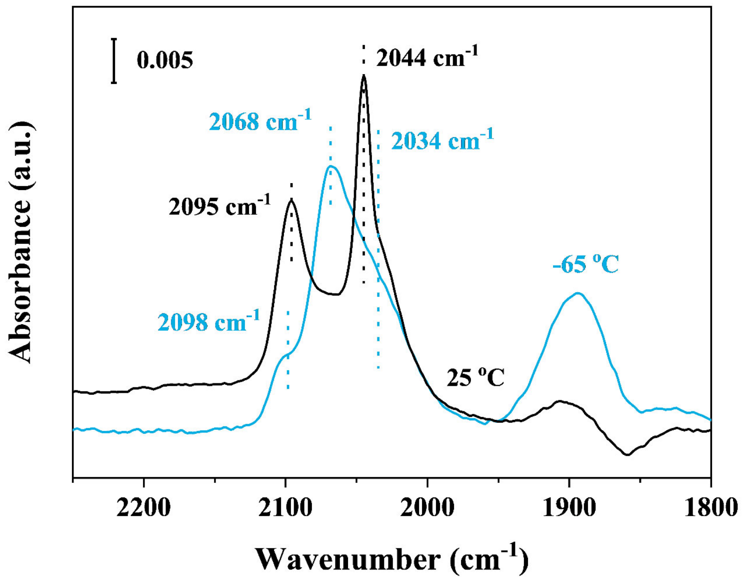

Figure S69. IR studies of CO adsorption at 25 °C (black line) and -65 °C (blue line) on Rh-MFI-calred sample.

Next, the role of the conditions used prior to CO adsorption experiments are shown by selecting 300 vs 80 °C as temperature for sample evacuation. As can be seen in Figure S70 the re-dispersion of metal species is also favoured when the evacuation takes place at high temperatures.

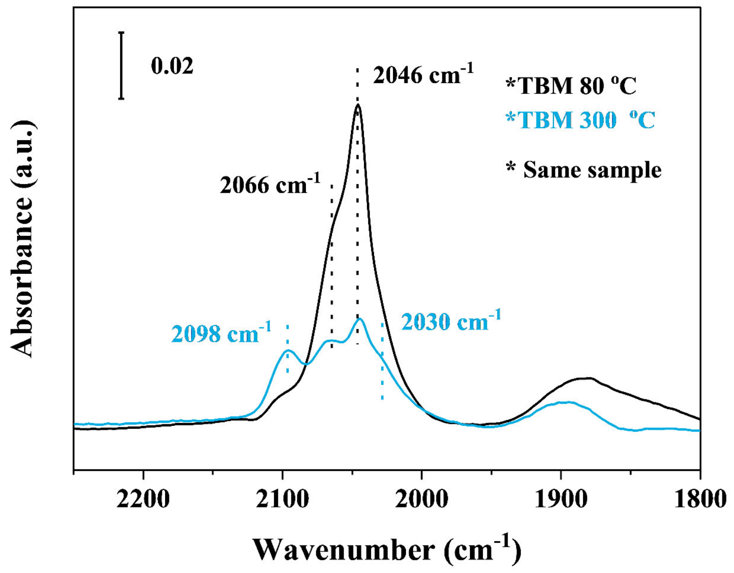

Figure S70. IR studies of CO adsorption at -65 °C (blue line) on Rh-MFI-calred sample after evacuation at 300 °C (blue line) and 80 °C (black line).

According to these results, the experimental set-up used in our work for identification of Rh species has been carefully selected as described in the experimental section

Appendix 2 Determination of Rh^{3+} by IR-CO experiments

Rh^{3+} easily reduced to Rh^{+} in presence of CO even when operating at -65 °C as shown in Figure S71, making quantitative determination of Rh^{3+} challenge.

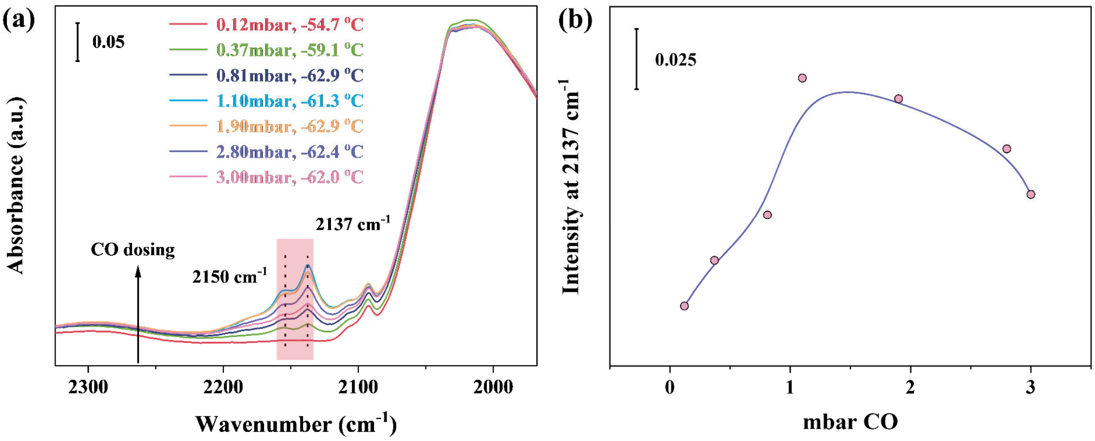

Figure S71. (a) Evolution of the IR band at 2137 cm^{-1} due to Rh^{3+} at increasing CO dosing and at a temperature of -65 °C, (b) evolution of peak area with CO dosing.

Supplementary References

1. Simonelli L, *et al.* CLÆSS: The hard X-ray absorption beamline of the ALBA CELLS synchrotron. *Cogent Phys.* **3**, 1231987 (2016).
2. Ravel B, Newville M. ATHENA, ARTEMIS, HEPHAESTUS: data analysis for X-ray absorption spectroscopy using IFEFFIT. *J. Synchrotron Radiat.* **12**, 537-541 (2005).
3. Newville M. EXAFS analysis using FEFF and FEFFIT. *J. Synchrotron Radiat.* **8**, 96-100 (2001).
4. Rehr JJ, Albers RC. Theoretical approaches to X-ray absorption fine structure. *Rev. Mod. Phys*. **72**, 621-654 (2000).
5. Wyckoff, R. W. G. Crystal Structures. Second Edition, *Interscience Publishers*, **1**, 7-83, New York (1963).
6. Jain A, et al. Commentary: The Materials Project: A materials genome approach to accelerating materials innovation. *APL Materials*. **1**, 011002 (2013).
7. Bevington, P. R., & Robinson, D. K. Data Reduction and Error Analysis for the Physical Sciences. Second edition, *McGraw-Hill*, New York (1992).
8. Newville M. Larch: An analysis package for XAFS and related spectroscopies. *J Phys Cond Ser*. **430**, 012007 (2013).

9. Sterbinsky GE, Heald SM. A simple method for mitigating error in the fixed-offset of a double-crystal monochromator. *J. Synchrotron Radiat.* **28**, 1737-1746 (2021).

10. Bolin TB, *et al.* In situ intermediate-energy X-ray catalysis research at the advanced photon source beamline 9-BM. *Catal. Today* **205**, 141-147 (2013).

11. Alvarado Rupflin L, *et al.* Platinum Group Metal Phosphides as Heterogeneous Catalysts for the Gas-Phase Hydroformylation of Small Olefins. *ACS Catal.* **7**, 3584-3590 (2017).

12. Huang N, *et al.* Insights into the Bimetallic Effects of a RhCo Catalyst for Ethene Hydroformylation: Experimental and DFT Investigations. *Ind. Eng. Chem. Res.* **59**, 18771-18780 (2020).

13. Ro I, *et al.* Synthesis of Heteroatom Rh–ReO_x Atomically Dispersed Species on Al_2O_3 and Their Tunable Catalytic Reactivity in Ethylene Hydroformylation. *ACS Catal.* **9**, 10899-10912 (2019).

14. Yamagishi T, *et al.* Selective formation of 1-propanol via ethylene hydroformylation over the catalyst originated from $RhVO_4$. *Catal. Commun.* **6**, 421-425 (2005).

15. Ichikawa M, *et al.* Selective hydroformylation of ethylene on rhodium-zinc-silica. An apparent example of site isolation of rhodium and Lewis acid-promoted carbonyl insertion. *J. Am. Chem. Soc.* **107**, 7216-7218 (1985).

16. Farpón MG, *et al.* Rhodium Single-Atom Catalyst Design through Oxide Support Modulation for Selective Gas-Phase Ethylene Hydroformylation. *Angew. Chem. Int. Ed.* **62**, e202214048 (2023).

17. Ro I, *et al.* Bifunctional hydroformylation on heterogeneous Rh-WO_x pair site catalysts. *Nature* **609**, 287-292 (2022).

18. Fusi A, *et al.* A molecular approach to heterogeneous catalysis. Ethylene hydroformylation catalysed by silica-supported $[Rh_{12}(CO)_{30}]^{2-}$ cluster anion: influence of the countercations Li^+, Na^+, K^+, Zn^{2+}. *J. Mol. Catal. A-Chem.* **107**, 255-261 (1996).

19. Hanaoka T, *et al.* Ethylene hydroformylation and carbon monoxide hydrogenation over modified and unmodified silica supported rhodium catalysts. *Catal. Today* **58**, 271-280 (2000).

20. Tomishige K, *et al.* Promoting Effect of Mo on Alcohol Formation in Hydroformylation of Propylene and Ethylene on Mo-Rh/SiO_2. *Catal. Lett.* **103**, 15-21 (2005).

21. Balakos MW, Chuang SSC. Dynamic and LHHW Kinetic Analysis of Heterogeneous Catalytic Hydroformylation. *J. Catal.* **151**, 266-278 (1995).

22. Kitamura-Bando K, *et al.* Direct observation of unusual CO insertion on a new SiO_2-attached Rh dimer catalyst by FTIR. *J. Chem. Soc., Chem. Commun*. 253-255 (1990).

23. Qi L, *et al.* Ethene Hydroformylation Catalyzed by Rhodium Dispersed with Zinc or Cobalt in Silanol Nests of Dealuminated Zeolite Beta. *J. Am. Chem. Soc.* **145**, 2911-2929 (2023).

24. Mao Z, *et al.* Comparison of Heterogeneous Hydroformylation of Ethylene and Propylene over $RhCo_3$/MCM-41 Catalysts. *ACS Catal.* **11**, 14575-14585 (2021).

25. Zakem G, Christopher P. Active Site Entropy of Atomically Dispersed Rh/Al_2O_3 Catalysts Dictates Activity for Ethylene Hydroformylation. *ACS Catal.* **13**, 5502-5515 (2023).

26. Xie Z, *et al.* Reactions of CO_2 and ethane enable CO bond insertion for production of C3 oxygenates. *Nat. Commun.* **11**, 1887 (2020).

27. Navidi N, Thybaut JW, Marin GB. Experimental investigation of ethylene hydroformylation to propanal on Rh and Co based catalysts. *Appl. Catal. A-Gen.* **469**, 357-366 (2014).

28. Liu J, *et al*. Promoting effect of Al on tethered ligand-modified Rh/SiO_2 catalysts for ethylene hydroformylation. *Appl. Catal. A-Gen.* **492**, 127-132 (2015).

29. Huang N, *et al*. Promotion of diphosphine ligands ($PPh_2(CH_2)_nPPh_2$, n = 1, 2, 3, 5, 6) for supported Rh/SiO_2 catalysts in heterogeneous ethene hydroformylation. *Mol. Catal.* **511**, 111736 (2021).

30. Weiß A, *et al*. Modification of nitrogen doped carbon for SILP catalyzed hydroformylation of ethylene. *Catal. Sci. Technol.* **7**, 5562-5571 (2017).

31. Jiang M, et al. Ultrastable 3V-PPh_3 polymers supported single Rh sites for fixed-bed hydroformylation of olefins. *J Mol Catal A-Chem*. **404-405**, 211-217 (2015).

32. Hülsey MJ*, et al.* In situ spectroscopy-guided engineering of rhodium single-atom catalysts for CO oxidation. *Nat. Commun.* **10**, 1330 (2019).

33. Liu B*, et al.* Heterogeneous hydroformylation of alkenes by Rh-based catalysts. *Chem* **8**, 2630-2658 (2022).

34. Hanf S*, et al.* Current State of the Art of the Solid Rh-Based Catalyzed Hydroformylation of Short-Chain Olefins. *Catalysts* **10**, 510 (2020).

35. Miessner H. Surface Chemistry in a Zeolite Matrix. Well-Defined Dinitrogen Complexes of Rhodium Supported on Dealuminated Y Zeolite. *J. Am. Chem. Soc.* **116**, 11522-11530 (1994).

36. Wovchko EA, Yates JT. Nitrogen Chemisorption on the Coordinatively Unsaturated Rh Site on Al_2O_3. *J. Am. Chem. Soc.* **118**, 10250-10256 (1996).

37. Vayssilov GN, Rösch N. A New Interpretation of the IR Bands of Supported Rh(I) Monocarbonyl Complexes. *J. Am. Chem. Soc.* **124**, 3783-3786 (2002).

38. Zheng A*, et al.* ^{31}P NMR Chemical Shifts of Phosphorus Probes as Reliable and Practical Acidity Scales for Solid and Liquid Catalysts. *Chem. Rev.* **117**, 12475-12531 (2017).

39. Zhao P, et al. Theoretical study on ^{31}P NMR chemical shifts of phosphorus-modified CHA zeolites. *Microporous Mesoporous Mater*. **294**, 109908 (2020).

40. Gao P, *et al.* Phosphorus coordinated Rh single-atom sites on nanodiamond as highly regioselective catalyst for hydroformylation of olefins. *Nat. Commun.* **12**, 4698 (2021).

41. Sordelli L, *et al.* Characterization and catalytic performances of alkali-metal promoted Rh/SiO_2 catalysts for propene hydroformylation. *J. Mol. Catal. A-Chem.* **204-205**, 509-518 (2003).

42. Yamagishi T, *et al.* Catalytic performance and characterization of $RhVO_4/SiO_2$ for hydroformylation and CO hydrogenation. *J. Mol. Catal. A-Chem.* **244**, 201-212 (2006).

43. Fang C-Y*, et al.* Synthesis of $Rh_6(CO)_{16}$ in Supercages of Zeolite HY: Reaction Network and Kinetics of Formation from Mononuclear Rhodium Precursors via $Rh_4(CO)_{12}$ Facilitated by the Water Gas Shift Half-Reaction. *J. Phys. Chem. C* **124**, 2513-2520 (2020).

44. Lang R, et al. Hydroformylation of Olefins by a Rhodium Single-Atom Catalyst with Activity Comparable to $RhCl(PPh_3)_3$. *Angew. Chem. Int. Ed.* **55**, 16054-16058 (2016).

45. Ivanova E, Hadjiivanov K. Polycarbonyls of Rh^+ formed after interaction of CO with Rh–MFI: an FTIR spectroscopic study. *Phys. Chem. Chem. Phys.* **5**, 655-661 (2003).

46. Matsubu JC, *et al.* Isolated Metal Active Site Concentration and Stability Control Catalytic CO_2 Reduction Selectivity. *J. Am. Chem. Soc.* **137**, 3076-3084 (2015).

47. Yan G, *et al.* Reaction product-driven restructuring and assisted stabilization of a highly dispersed Rh-on-ceria catalyst. *Nat. Catal.* **5**, 119-127 (2022).
